# First insight into the somatic mutation burden of neurofibromatosis type 2-associated grade I and grade II meningiomas: a case report comprehensive genomic study of two cranial meningiomas with vastly different clinical presentation

**DOI:** 10.1186/s12885-017-3127-6

**Published:** 2017-02-13

**Authors:** Ramita Dewan, Alexander Pemov, Amalia S. Dutra, Evgenia D. Pak, Nancy A. Edwards, Abhik Ray-Chaudhury, Nancy F. Hansen, Settara C. Chandrasekharappa, James C. Mullikin, Ashok R. Asthagiri, NISC Comparative Sequencing Program, John D. Heiss, Douglas R. Stewart, Anand V. Germanwala

**Affiliations:** 1 Surgical Neurology Branch, National Institute of Neurological Disorders and Stroke, National Institutes of Health, Bethesda, MD USA; 2 Clinical Genetics Branch, Division of Cancer Epidemiology and Genetics, National Cancer Institute, Rockville, MD USA; 3 Genetic Disease Research Branch, National Human Genome Research Institute, National Institutes of Health, Bethesda, MD USA; 4 Cancer Genetics and Comparative Genomics Branch, National Human Genome Research Institute, National Institutes of Health, Bethesda, MD USA; 5 NIH Intramural Sequencing Center, National Human Genome Research Institute, National Institutes of Health, Rockville, MD USA; 6 Department of Neurological Surgery, University of Virginia School of Medicine, Charlottesville, VA USA; 7 Department of Neurological Surgery, Loyola University Stritch School of Medicine, Maywood, IL USA; 8 Department of Otolaryngology, Edward Hines, Jr. VA Hospital, 2160 South First Avenue, Maywood, IL 60153 USA

**Keywords:** Whole exome sequencing, Single nucleotide polymorphism, Spectral karyotyping, *NF2* gene, Somatic mutation, Case report

## Abstract

**Background:**

Neurofibromatosis type 2 (NF2) is a rare autosomal dominant nervous system tumor predisposition disorder caused by constitutive inactivation of one of the two copies of *NF2*. Meningiomas affect about one half of NF2 patients, and are associated with a higher disease burden. Currently, the somatic mutation landscape in NF2-associated meningiomas remains largely unexamined.

**Case presentation:**

Here, we present an in-depth genomic study of benign and atypical meningiomas, both from a single NF2 patient. While the grade I tumor was asymptomatic, the grade II tumor exhibited an unusually high growth rate: expanding to 335 times its initial volume within one year. The genomes of both tumors were examined by whole-exome sequencing (WES) complemented with spectral karyotyping (SKY) and SNP-array copy-number analyses. To better understand the clonal composition of the atypical meningioma, the tumor was divided in four sections and each section was investigated independently. Both tumors had second copy inactivation of *NF2*, confirming the central role of the gene in meningioma formation. The genome of the benign tumor closely resembled that of a normal diploid cell and had only one other deleterious mutation (*EPHB3*). In contrast, the chromosomal architecture of the grade II tumor was highly re-arranged, yet uniform among all analyzed fragments, implying that this large and fast growing tumor was composed of relatively few clones. Besides multiple gains and losses, the grade II meningioma harbored numerous chromosomal translocations. WES analysis of the atypical tumor identified deleterious mutations in two genes: *ADAMTSL3* and *CAPN5* in all fragments, indicating that the mutations were present in the cell undergoing fast clonal expansion

**Conclusions:**

This is the first WES study of NF2-associated meningiomas. Besides second *NF2* copy inactivation, we found low somatic burden in both tumors and high level of genomic instability in the atypical meningioma. Genomic instability resulting in altered gene dosage and compromised structural integrity of multiple genes may be the primary reason of the high growth rate for the grade II tumor. Further study of *ADAMTSL3* and *CAPN5* may lead to elucidation of their molecular implications in meningioma pathogenesis.

**Electronic supplementary material:**

The online version of this article (doi:10.1186/s12885-017-3127-6) contains supplementary material, which is available to authorized users.

## Background

Neurofibromatosis type 2 (NF2) is an autosomal dominant tumor syndrome characterized by the growth of multiple neoplasms within the central nervous system. Although bilateral vestibular schwannomas are the hallmark of NF2, meningiomas are the second most frequent intracranial tumor, and occur in about 52% of NF2 patients [1, 2]. Benign meningiomas (WHO grade I) feature a 5-year tumor recurrence rate of 5% as compared to 50–80% for anaplastic meningiomas (grade III), highlighting the importance of elucidating the molecular mechanisms which contribute to tumor progression [3].

The most common genetic mutation in meningiomas is *NF2* inactivation, which is observed not only in NF2-associated tumors, but also in 47 to 72% of sporadic meningiomas, and is thus considered an integral step for meningioma tumor initiation [4–6]. Recent studies utilizing high throughput whole-exome and whole-genome sequencing have identified two distinct subtypes of sporadic meningiomas: tumors with or without an inactivated *NF2* gene [7, 8]. Sporadic meningiomas with disrupted *NF2* tend to display greater genomic instability (including several cases of chromothripsis) and higher grades than non-*NF2* meningiomas. Non-*NF2* tumors have been shown to contain recurrent oncogenic mutations in *AKT1*, *KLF4*, *TRAF7* and *SMO*, indicating the alternate involvement of the PI3K-AKT and Hedgehog signaling pathways.

NF2-associated meningiomas are rarer than their sporadic counterparts and far fewer studies have investigated the genetics underlying their initiation and progression. Two case series evaluated meningiomas from NF2 patients only for the allelic imbalances most commonly observed in sporadic meningiomas, and confirmed frequent somatic inactivation of the *NF2* gene, as well as losses of chromosome arms 1p, 6q, 9p, 10q, 14q and 18q [9, 10]. A more recent study used single nucleotide polymorphism array analysis to report increased chromosomal instability with increasing grade in NF2-associated meningiomas [11].

Here, we present an in-depth genomic study of grade I and grade II meningiomas that resided in close proximity in the brain of an NF2 patient. The tumors contained the same *NF2* germline mutation and similar somatic hits affecting the normal remaining copy of the gene, yet differed drastically in genomic architecture and growth rate. The tumors were investigated using whole-exome sequencing complemented with SKY and SNP-array copy-number analysis.

## Case presentation

### Materials and methods

#### Patient information

A 35-year-old woman was enrolled in the Institutional Review Board (IRB)-approved NF2 natural history study (NIH#08-N-0044) at the National Institute of Neurologic Disease and Stroke (NINDS). Prior MR imaging confirmed the NF2 Manchester diagnostic criteria of bilateral vestibular schwannoma in addition to numerous other significant findings: intracranial schwannomas involving cranial nerves V, VII, and VIII, intracranial meningiomas, cervical ependymomas, schwannomas along the cauda equina, and cervicothoracic meningiomas.

In preparation for surgery, the patient underwent frameless stereotactic navigation imaging on a 1.5 Tesla MRI scanner with and without gadolinium. 1-mm axial images were obtained with sagittal and coronal reconstruction. Image guidance registration was performed intraoperatively using facial registration.

#### Surgical resection

A single image-guided right frontal craniotomy was used to resect an anterior grade II meningioma, in four discrete sections, and a posterior grade I meningioma. The two meningiomas were separated by an intervening section of normal brain, and were resected through a single image-guided right frontal craniotomy. The anterior grade II meningioma was noted to be soft and was removed in four anatomically discrete sections with alternating steps of circumferential dissection and suction. The posterior grade I meningioma was noted to be firm and was removed en bloc.

#### Histopathology analysis

Tumor specimens were fixed in 10% buffered formalin immediately after removal, processed overnight, and subsequently embedded in paraffin. Five μm-thick sections were obtained from the paraffin blocks, and stained using the standard hematoxylin and eosin method.

#### DNA extraction

Frozen tumor tissue was processed with Proteinase K, and DNA extraction was completed using the phenol:chloroform procedure. Frozen tumor tissue was minced with a scalpel, washed once in PBS, pH 7.4, and incubated in a solution containing 100 mM TrisHCl, pH 8.0, 5 mM EDTA, 0.5% SDS and 200 μg/mL Proteinase K (Invitrogen, Grand Island, NY) at 55 °C for 2–3 h or at 37 °C overnight. DNA was extracted by the phenol:chloroform procedure and precipitated with ice cold isopropanol. DNA pellets were air dried, re-suspended in 10 mM TrisHCl, pH 7.4 and 0.1 mM EDTA, aliquoted and stored at −20 °C.

#### Whole-exome sequencing (WES) of tumor and normal DNA

Capture of the coding portion (exome) of genomic DNA and library preparation for next generation sequencing was done using Roche NimbleGen (Madison, WI) SeqCap EZ Exome + UTR library (64 Mb of coding exons and miRNA regions plus 32 Mb

untranslated regions (UTR)) according to the manufacturer’s instructions. As an input, 1 μg of tumor and matching normal genomic DNA was used. Sequencing was completed on the Illumina HiSeq 2500 system (Illumina, San Diego, CA, USA). Among the six exomes sequenced, the average breadth of coverage was 89% (range 88–90%), and the average depth of coverage was 66X (range 54X-78X).

Raw sequencing data was further processed using an analytical pipeline that included ELAND (Illumina, Inc.) for initial alignment to the reference human genome (GRCh37); Novoalign, v.2.08.02 [12] for local re-alignment; bam2mpg for genotype calling and calculation of the quality score Most Probable Genotype (MPG) [13] and ANNOVAR for functional annotation of genetic variants [14, 15]. The resulting data was formatted in VarSifter [16] format for further filtering.

Filtering consisted of removing all non-coding variants and nucleotides whose genotypes were identical in both the tumor and corresponding germline DNA, whose quality score (Most Probable Genotype, MPG) was less than 10 in either tumor or normal DNA, and whose ratio of quality score to depth of coverage was below 0.5 in germline DNA and below 0.4 in tumor DNA. All common variants (variants with minor allele frequency above 0.03 in ClinSeq and 1000 Genomes databases) were also removed. The resulting set was annotated with PolyPhen, SIFT and CADD tools to identify pathogenic mutations.

#### Sanger validation of mutations identified by WES

PCR primers were designed using Primer3 (v. 0.4.0) online software [17]. PCR amplification was conducted using a 20 μL reaction mixture containing 20–50 ng of genomic DNA, 1x reaction buffer, 1.5 mM MgCl2, 4 dNTPs at 250 μM each, 10 pmole each of forward and reverse primers, and 2 units of ThermoFisher Scientific Taq DNA polymerase (Waltham, MA). PCR products were analyzed on Agilent 2100 BioAnalyzer (Santa Clara, CA) and sent for Sanger sequencing to ACGT, Inc. (Wheeling, IL). Sequencing was done on ABI 3730 DNA Analyzer, the data was processed with GeneMapper v.3.7 software (ThermoFisher Scientific), and the phred quality score for sequenced nucleotides was visualized using CodonCode Aligner v.6.0.2 (CodonCode Corp., Centerville, MA). Sequencing reaction tracks were visualized using FinchTV v.1.5.0 (Geospiza, Inc., Seattle, WA) and CodonCode Aligner software.

#### Sanger sequencing of NF2

Sanger sequencing of *NF2* was conducted by Prevention Genetics (Marshfield, WI), a CLIA-certified DNA testing lab. PCR was used to amplify all *NF2* coding exons, as well as ~20 bp flanking intronic or other non-coding sequence. Sequencing was performed separately in both the forward and reverse directions and all differences from the reference sequence were reported.

#### SNP-array analysis

SNP genotyping was performed using HumanOmniExpressExome-8v1.2 Illumina BeadChip arrays (Illumina, San Diego, CA) per the manufacturer’s instructions. The arrays were read using the iScan platform (Illumina), and visualized with GenomeStudio v.2011.1 software (Illumina). The call rate for all the DNA samples was >99%. Genomic coordinates are per hg19.

#### Copy-number variation analysis

Copy-number variation (CNV) analysis of all tumor samples was performed using Nexus Copy Number software v.6.1 (BioDiscovery, Inc., Hawthorne, CA). “Allelic imbalance” refers to a locus with B-allele frequency classes other than 0, 0.5 or 1. Allele-Specific Copy number Analysis of Tumors (ASCAT) (v2.1) analysis of the data was performed as described by Van Loo and co-authors [18].

#### Spectral karyotyping (SKY)

Metaphase slide preparations were made from cultured meningioma primary cell cultures established from the grade II meningioma and hybridized with commercially available SKY probe set (Applied Spectral Imaging Inc., Carlsbad, CA) according to the manufacturer’s instructions. Mitotic arrest with colcemid (0.015 μg/mL, 2–4 h) (GIBCO, Gaithersburg, MD) was followed by hypotonic treatment (75 mM KCl, 20 min, 37 °C) and fixation in methanol–acetic acid mixture (3:1).

### Results

#### Clinical presentation

**Fig. 1:**
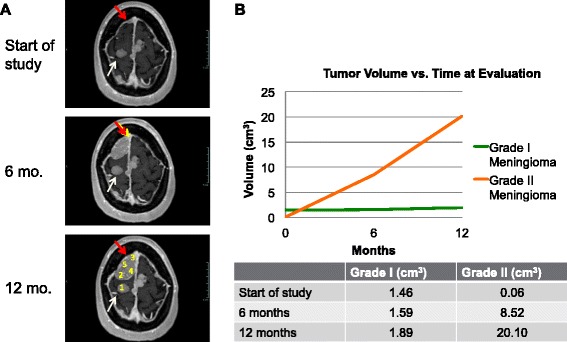
MRI and growth rate analysis of the grade I and grade II meningiomas. **a** MRI images of the patient’s tumors at the start of the study, and 6 and 12 month time points. The slowly and rapidly growing tumors are indicated with white and red arrows, respectively. Numbers 1 through 5 on the bottom image show the tumor samples taken for genomic analysis: 1- grade I (*slowly growing*) meningioma, and 2 through 5 - regions of the grade II (*rapidly growing*) meningioma. **b** Growth rate volumetric analysis of the tumors shown in **a**, with the grade II tumor displaying exponential growth

The patient, over 12 months of enrollment, noted progressively worsening right frontal headaches. The patient had a family history of NF2 and was deaf from bilateral vestibular schwannomas that were successfully treated in the past with radiosurgery. Her neurological exam was notable only for bilateral deafness. Over the 12-month period, the patient was noted to have significant radiographic progression of a right anterior frontal meningioma, increasing to 335 times its original volume, while other brain and spine tumors remained relatively stable (Fig. 1). Due to the tumor’s symptomatic radiographic progression, surgical resection was offered. Consent to remove an adjacent, stable posterior frontal tumor was also obtained in the setting that it was accessible for resection without posing additional risk. The patient had an unremarkable hospital course and post-operative imaging confirmed gross total resection of both lesions. At six weeks follow-up, the patient noted significant improvement in her headaches.

#### Histopathological analysis of the tumors

**Fig. 2:**
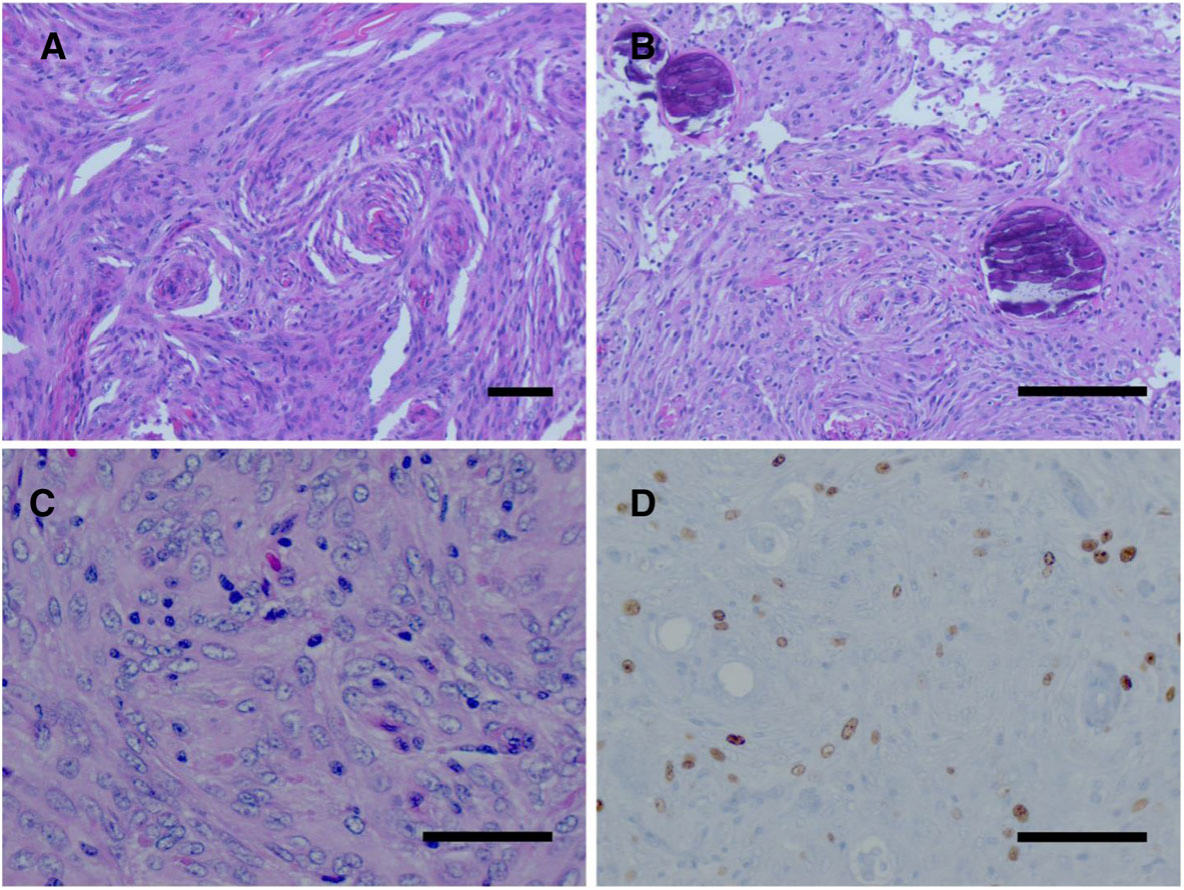
Histological appearance of benign and atypical meningioma. **a** Histological analysis of the posterior specimen revealed a grade I meningioma with typical whorl formations (bar 100 μm) and **b** psammoma bodies (bar 100 μm). **c** The anterior tumor revealed a grade II meningioma with increased cellularity, nuclear pleomorphism, and prominent nucleoli as indicated by H&E (bar 50 μm) and **d** an increased proliferation index by MIB-1 labeling (bar 50 μm)

Histological analysis of the anterior tumor revealed a grade II meningioma with increased cellularity, nuclear pleomorphism, cells with prominent nucleoli and areas of patternless growth. Increased proliferation index as evidenced by immunostain for MIB-1 antigen was also observed. Pathology of the posterior specimen revealed a grade I meningioma consisting of monomorphic cells having ovoid to elongated nuclei, multiple cellular whorls, scattered psammoma bodies and rare mitotic figures (Fig. 2).

#### Germline and somatic mutations in the NF2 gene

Sequencing of exons and small flanking intronic regions of the *NF2* gene from peripheral white blood cell DNA (germline) identified a constitutive mutation in the intronic region, two nucleotides upstream of the 5′-end of exon 13: c.1341-2A > C. This mutation likely disrupts the acceptor site of intron 12, thus affecting RNA splicing, and has been previously reported as pathogenic (Human Gene Mutation Database, CS115647) by Ellis and co-authors [19]. It has not been previously annotated in the ExAC (mean coverage 15x), 1000 Genomes, or ESP datasets [20].

**Fig. 3:**
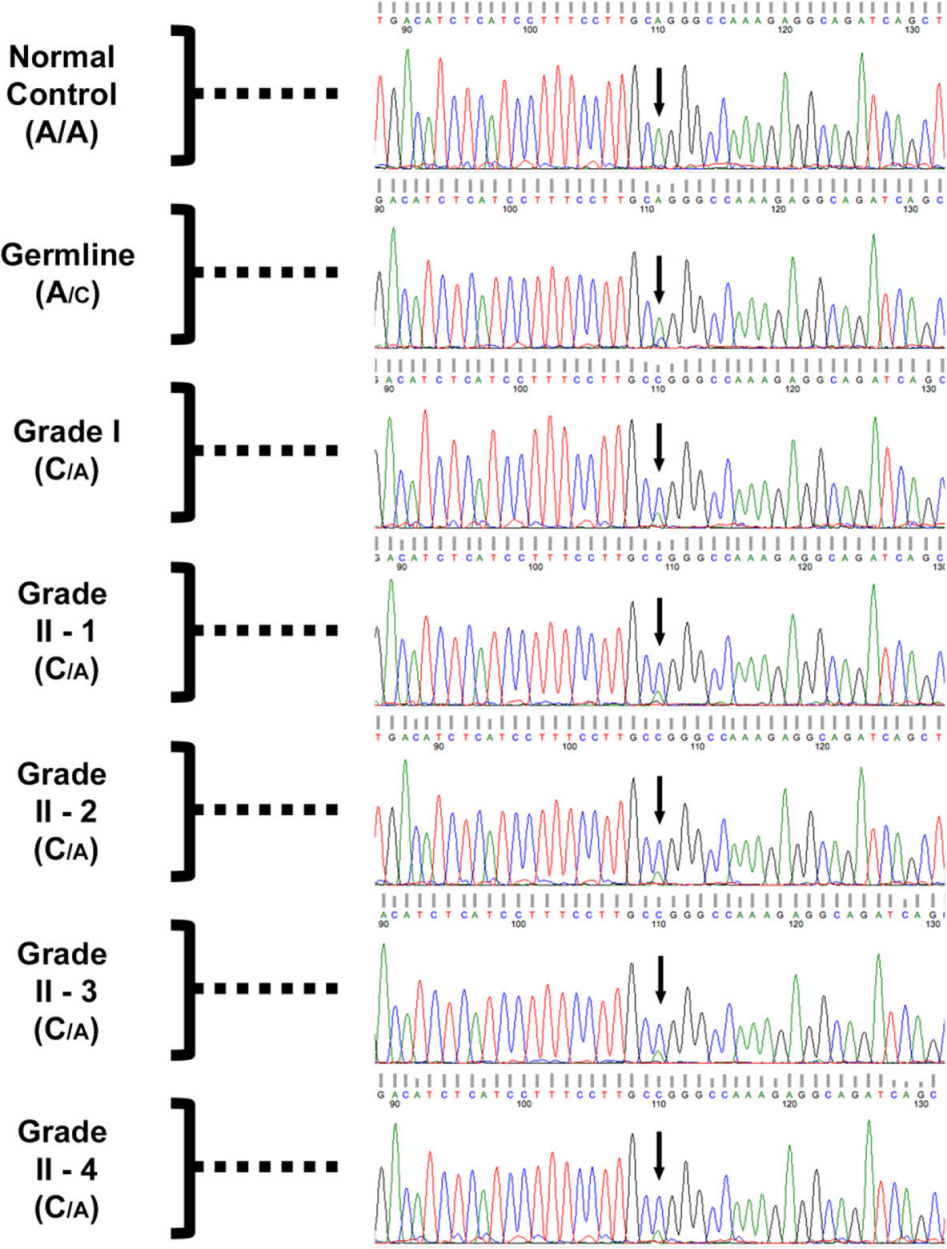
Sanger analysis of grade I and grade II tumors. Sequencing tracks of the genomic region surrounding the splice site mutation (Exon 13; c.1341-2A > C) upstream of exon 13 in the *NF2* gene in normal control DNA, and germline and tumor DNA of the NF2 patient. “Grade II-1” through “Grade II-4” labels denote the four fragments of the grade II meningioma analyzed in this study. *Arrows* point at the mutant nucleotide, which was heterozygous in germline and all tumor fragments. The relative font size for the alleles in each sample reflects the difference in signal strength for A and C nucleotides at the site of the mutation

The mutation was identified in the patient’s peripheral white blood cell DNA by a CLIA-certified genetic testing lab (Prevention Genetics, Marshfield, WI, data not shown). We performed secondary confirmation using PCR amplification followed by Sanger sequencing of the DNA fragment containing the mutant base. Both Prevention Genetics (PG) and our analyses of germline DNA revealed the presence of a mutant base C in the sequence (Fig. 3). Examination of phred quality scores for the mutant base and several surrounding nucleotides revealed lower values in the patient’s germline DNA compared to the normal control (Additional file 1). This was in agreement with visual evaluation of the chromatogram peaks: the presence of C signal in addition to A signal at this nucleotide position made an unambiguous call less certain, resulting in a lower quality score (Fig. 3 and Additional file 1).

We noticed that the mutant signal (C) was weaker than that of the reference allele (A) (Fig. 3, see “Germline” panel). A similar A-to-C signal ratio was observed in both the plus and minus strand DNA sequences in both PG and our analyses (data in Fig. 3 is shown for the plus strand only). A-to-C substitution in the sequence 5′-GG[A]GGGCC-3′ converts it to a GC-rich 8 nucleotide-long stretch 5′-GG[C]GGGCC-3′. Such sequences can be more difficult to analyze due to the secondary DNA structure, which may explain the decreased mutant base C signal.

**Fig. 4:**
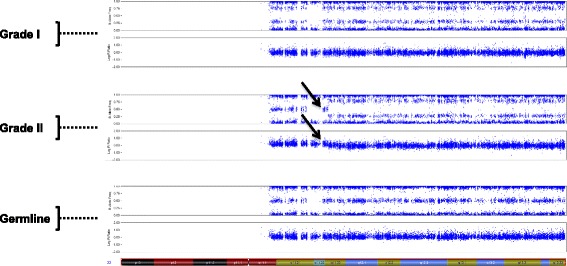
Somatic inactivation of *NF2* via chromosome 22 deletion in grade I and grade II meningiomas. SNP-array analysis of grade I (*top* panel) and grade II (*middle* panel) meningiomas and normal (germline) DNA (*bottom* panel). Each panel consists of two plots: B-allele frequency (*top*) and intensity (*bottom*). Cytoband map of chromosome 22 is shown on the bottom of the figure. The arrows show the start of the deletion region in the grade II meningioma. Data for only one of the four fragments of grade II tumor is shown, since the data for the remaining three is essentially the same

Copy number analysis of the tumors revealed that the grade I meningioma contained loss of entire chromosome 22, and all fragments of the grade II meningioma harbored loss of chromosome 22q (Fig. 4). Thus, loss of heterozygosity (LOH) was the likely mechanism of somatic *NF2* inactivation. PCR amplification followed by Sanger-sequencing of the region surrounding c.1341-2A > C substitution in all tumor samples revealed mostly homozygous mutant genotype (C/C), confirming loss of the remaining wild-type copy of the *NF2* gene. One can observe a weak reference allele A signal in the tumor sequencing chromatograms, due to the presence of 20–30% of non-tumor stromal cells that still contain the reference allele (Fig. 3, bottom four panels, “Grade I’, “Grade II-1”-“Grade II-4”, green peaks). These observations were also confirmed by lower phred scores of the mutant nucleotide in all tumor samples compared to normal DNA control (Additional file 1).

#### Copy-number SNP-array analysis

**Fig. 5:**
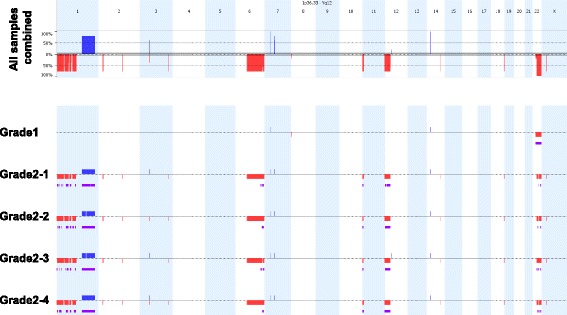
Genomic distribution of CNVs in grade I and four fragments of grade II meningiomas. Chromosomal deletions and duplications in individual samples (*lower* panel) and as aggregation of all samples (*upper* panel) are shown as red and *blue* bars, respectively. Regions of allelic imbalance are shown in *purple*. Individual chromosomes, 1 through 22 and X, are shown as alternating *light blue* and *white* columns. The height of the *red* and *blue* bars in the upper panel reflects the number of samples the CNV is detected in. The percent scale is shown on the left of the upper panel

We investigated copy-number variation (CNV) in the grade I and grade II meningiomas on Illumina SNP-arrays, followed by Nexus CNV software analysis (Fig. 5). Besides LOH of entire chromosome 22, the grade I tumor contained only a single 22 kilobase deletion on chromosome 8p23.2, in an intronic region of *CSMD1*. In contrast, we observed multiple gains and losses, ranging from a few kilobases to 100 megabases in eleven different chromosomes in the grade II tumor (Table 1 and Additional file 2). The deleted and amplified regions in the grade II tumor harbor more than 3000 genes (Additional file 3), 54 of which are known cancer genes (Additional file 4).

**Table 1:** Grade II meningioma chromosomal aberrations identified by SNP-array analysis

Chromosome	Mutation	Location	Previously reported?
1	Loss	p36.33 to p34.2	Yes
	Loss	p33 to p31.3	Yes
	Gain	p31.3 to p31.1	No
	Loss	p31.1	Yes
	Loss	p31.1 to p22.3	Yes
	Loss	p22.1 to p13.2	Yes
	Gain	q21.2 to q21.3	Yes
	Gain	q21.3 to q42.13	Yes
2	Loss	p23.2	Yes
	Loss	q22.1	Yes
3	Loss	p14.2	Yes
	Gain	p14.2	No
	Loss	q26.31	Yes
6	Loss	q12	Yes
	Loss	q13-q27	Yes
7	Gain	q11.21	No
11	Loss	p15.5 to p15.4	No
12	Loss	p13.33 to p11.21	Yes
14	Loss	q31.1	Yes
18	Loss	q23	Yes
22	Loss	q11.23-q13.33	Yes
X	Loss	p21.1	Yes

#### Spectral karyotyping (SKY) of grade II meningioma cells

SNP-array analysis, while providing data on copy-number variation in the genome, does not permit detection of structural chromosomal rearrangements such as inversions and translocations. To address this, we used primary cell cultures established from the grade II meningioma for SKY analysis. Viable cultures from the grade II-2, grade II-3 and grade II-4 tumor fragments contained cells of normal ploidy, and both unaffected cells (20–40%) and cells with multiple chromosomal translocations (Table 2). Within each culture, approximately half of the translocations were recurrent. In one culture (grade II-2), we observed highly abnormal cells with chromosomes that appeared “shattered” or broken into multiple fragments.

**Table 2:** Chromosomal translocations identified by SKY analysis in grade II meningioma fragments 2, 3 and 4

	Metaphase state			
	Normal	Abnormal		Shattered
Sample		Non-recurrent	Recurrent	
Grade II-2	40%	40%	0%	20%
		46, XX, t(10;18)		
		46, XX, t(1;8)		
		46, XX, t(5;14)		
Grade II-3	20%	30%	50%	0%
		46, XX, t(11;8)	46, XX, t(8;8), t(11;12)	
		46, XX, t(1;2), t(2;4), t(7;8), t(9;21)		
		46, XX, t(1;3;22), t(11;21), t(2;18)		
Grade II-4	20%	0%	80%	0%
			46, XX, t(3;13;14), t(17;21)	
			46, XX, t(3;13;14), t(17;21), inv(12)	

Percent of normal metaphases and metaphases containing chromosomal translocations (abnormal) is shown in the top row for each meningioma grade II primary tissue culture analyzed (Grade II-2, II-3 and II-4, note that culture from fragment II-1 could not be established). Abnormal metaphases were further divided into recurrent or non-recurrent. Fragment 2 of the tumor (Grade II-2) also contained 20% of cells with highly fragmented, shattered chromosomes

#### Whole-exome sequencing (WES) of tumors and Sanger verification of mutations

After WES data processing and filtering, we identified two potentially damaging somatic mutations in the grade I meningioma, and nine somatic mutations in the four fragments of the grade II meningioma (Additional file 5). Of the nine mutations in the grade II meningioma, two were found in all four fragments.

Of the two mutations in the grade I meningioma, one (*EPHB3*) mutation was verified by Sanger sequencing, and of the nine mutations in the grade II tumor, two (*CAPN5* and *ADAMTSL3*) were verified by Sanger sequencing. Importantly, the mutations in *CAPN5* and *ADAMTSL3* were detected by WES in all four fragments of grade II tumor and were verified by Sanger in all four fragments as well, suggesting that these mutations were likely present in the cell undergoing fast clonal expansion.

## Discussion

To our knowledge, this is the first whole-exome sequencing study of NF2-associated grade I and grade II meningiomas. Besides chromosome 22 loss, the genome of the grade I meningioma closely resembled that of a normal diploid cell, while the genome of the grade II tumor contained several chromosomal rearrangements previously observed in meningiomas, including losses in 1p, 2p, 2q, 3p, 3q, 6q, 12p, 14q, 18q, Xp, gain in 1q [21–25], and multiple translocations. Our observations confirm previous findings that inactivation of *NF2* is likely to be the primary step in NF2-associated meningioma formation [26]. In addition, we show that both benign and atypical tumors had a low somatic mutation burden. Although limited to a single patient, this data permits speculation that tumor progression to a higher grade likely occurs through multiple chromosomal gains, losses and translocations and to a lesser extent from the accumulation of point mutations and small indels.

Chromosomal translocations leading to the disruption of tumor suppressors or activation of proto-oncogenes are common in many neoplasms [27, 28]. Limited evidence suggests that chromosomal translocations may also be present in meningiomas [29] and systematic studies addressing this mechanism of tumorigenesis in meningiomas are emerging. We observed numerous chromosomal translocations (both balanced and unbalanced) as well as one case of highly irregular, shattered chromosomes. Interestingly, similar to the observation made by Brastianos et al. [7], close examination of SNP-array plots of chromosome 1 in the tumor revealed deletion of the 5′-half of the *NEGR1* gene (not shown). These findings suggest that structural aberrations might be more frequent than previously believed in *NF2*-driven familial and sporadic meningiomas, and could represent one of the mechanisms of genetic instability and routes of tumor progression to higher grades.

By analyzing the genomic architecture and somatic mutations in multiple fragments of the grade II tumor, we gained insight into the clonal evolution of this fast growing neoplasm. We observed not only a remarkably uniform pattern of chromosomal gains and losses, but also the consistent presence of the only two potentially pathogenic mutations, in *ADAMTSL3* and *CAPN5*, in all four fragments. These findings indicate that the aberrations were likely present in the initial cell undergoing fast clonal expansion, and that any of these aberrations/mutations could impact tumor progression and accelerate growth rate.

Germline mutations in *CAPN5* (Calpain 5), which encodes a calcium-dependent endopeptidase, have been associated with neovascular inflammatory vitreoretinopathy [30]. Though the role of the protein in neoplastic transformation is unclear, a recent study reported association of CAPN5 with promyelocytic leukemia nuclear bodies, which are involved in transcriptional regulation, cell differentiation, apoptosis, and cell senescence [31]. The protein encoded by *ADAMTSL3* (A Disintegrin And Metalloproteinase with TromboSpondin Like 3) is involved with extracellular matrix function and to cell–matrix interactions, and is frequently mutated and under-expressed in colorectal cancer [32]. The gene belongs to a large family of proteins associated with microfibrils in the extracellular matrix, thus mediating sequestration of the TGFB superfamily of proteins and affecting wide array of cellular functions such as adhesion, migration, proliferation and angiogenesis [33, 34].

The majority of meningiomas are benign and asymptomatic tumors that require little or no treatment [35, 36]. However, a subset of tumors becomes more clinically aggressive as they evolve toward atypical and anaplastic stages, causing increased morbidity and mortality. Remarkably, the tumors we investigated had the same *NF2* germline mutation, the same genetic background, similar chromosome 22 LOH and were residing within a few millimeters from one another in the patient’s brain, yet one remained as a slowly growing asymptomatic grade I meningioma and the other evolved into a fast growing grade II tumor. This observation underscores the importance of stochastic factors in meningioma progression, which are still poorly understood.

## Conclusions

We performed an in-depth genomic study of NF2-associated benign and atypical meningiomas. Both tumors had inactivated second copies of *NF2* and a low burden of somatic mutations. However, unlike the benign tumor, the atypical meningioma presented with widespread genomic aberrations, implying that chromosomal instability may be a key driving force in tumor progression. In addition, we identified two candidate driver genes, *CAPN5* and *ADAMTSL3,* which could contribute to the elevated growth rate of the grade II meningioma. Future efforts should be focused on understanding the mechanistic links between *NF2* deficiency and genomic instability.

## Additional files

Additional file 1: Phred scores for Sanger sequencing of the heterozygous mutant (red font) and surrounding homozygous wt nucleotides. Normal control sample is shown on the top (green fill). Phred scores for both forward and reverse sequencing reactions are shown. Note that a heterozygous nucleotide would usually affect (decrease) phred scores of a few adjacent wt homozygous nucleotides. (PDF 44 kb)
Additional file 2: Losses, gains and regions with allelic imbalance in the grade I meningioma and all four fragments of the grade II meningioma. (PDF 173 kb)
Additional file 3: Genes affected by gains and losses in the grade II meningioma. (PDF 884 kb)
Additional file 4: Cancer genes affected by losses or gains in the grade II meningioma. (PDF 62 kb)
Additional file 5: Mutations selected for Sanger verification. (PDF 115 kb)

## Abbreviations

*ADAMTSL3*: A Disintegrin And Metalloproteinase with TromboSpondin Like 3
*AKT1*: V-akt murine thymoma viral oncogene homolog 1
*BRD8*: Bromodomain containing 8
*CAPN5*: Calpain 5
CNV: Copy-number variation
*EPHB3*: EPH (ephrin) receptor B3
*KLF4*: Kruppel-like factor 4 (gut)
LOH: Loss of heterozygosity
NF2: Neurofibromatosis type 2
PI3K: Phosphatidylinositol-4,5-bisphosphate 3-kinase
SKY: Spectral karyotyping
*SMO*: Smoothened, frizzled class receptor
SNP: Single nucleotide polymorphism
TGFB: Transforming growth factor beta
*TRAF7*: TNF receptor associated factor 7
WES: Whole-exome sequencing
