# Supplementary material for: First insight into the somatic mutation burden of neurofibromatosis type 2-associated grade I and grade II meningiomas: a case report comprehensive genomic study of two cranial meningiomas with vastly different clinical presentation
Source: BMC Cancer. 2017 Feb 13;17:127. doi: 10.1186/s12885-017-3127-6 (PMC5307647; doi:10.1186/s12885-017-3127-6)
Supplement: Additional file 1: — Phred scores for Sanger sequencing of the heterozygous mutant (red font) and surrounding homozygous wt nucleotides. Normal control sample is shown on the top (green fill). Phred scores for both forward and reverse sequencing reactions are shown. Note that a heterozygous nucleotide would usually affect (decrease) phred scores of a few adjacent wt homozygous nucleotides. (PDF 44 kb) [file 12885_2017_3127_MOESM1_ESM.pdf]

**Additional File 1.** Phred scores for Sanger sequencing of the heterozygous mutant (red font) and surrounding homozygous wt nucleotides. Normal control sample is shown on the top (green fill). Phred scores for both forward and reverse sequencing reactions are shown. Note that a heterozygous nucleotide would usually affect (decrease) phred scores of a few adjacent wt homozygous nucleotides.

| Sample_ID/Nucleotide | C | T | T | G | C | A/C | G | G | G | C | C |
|---|---|---|---|---|---|---|---|---|---|---|---|
| Normal Control, Forward | 61 | 61 | 61 | 61 | 61 | 61 | 61 | 61 | 61 | 59 | 61 |
| Normal Control, Reverse | 61 | 59 | 61 | 61 | 61 | 61 | 61 | 61 | 61 | 61 | 61 |
| Germline, Forward | 61 | 61 | 59 | 61 | 52 | 39 | 44 | 59 | 59 | 61 | 61 |
| Germline, Reverse | 61 | 61 | 52 | 52 | 59 | 43 | 59 | 59 | 49 | 61 | 55 |
| Grade I tumor, Forward | 61 | 61 | 55 | 61 | 49 | 36 | 45 | 59 | 61 | 59 | 61 |
| Grade I tumor, Reverse | 61 | 61 | 19 | 23 | 31 | 13 | 39 | 39 | 24 | 61 | 59 |
| Grade II-1 tumor, Forward | 61 | 61 | 59 | 61 | 52 | 45 | 49 | 59 | 61 | 61 | 61 |
| Grade II-1 tumor, Reverse | 61 | 61 | 28 | 27 | 37 | 23 | 43 | 43 | 28 | 61 | 55 |
| Grade II-2 tumor, Forward | 61 | 59 | 59 | 61 | 52 | 49 | 52 | 59 | 61 | 61 | 61 |
| Grade II-2 tumor, Reverse | 61 | 61 | 28 | 27 | 36 | 24 | 35 | 45 | 34 | 59 | 37 |
| Grade II-3 tumor, Forward | 61 | 61 | 59 | 61 | 52 | 49 | 52 | 59 | 61 | 61 | 61 |
| Grade II-3 tumor, Reverse | 59 | 61 | 24 | 22 | 33 | 23 | 43 | 43 | 24 | 61 | 61 |
| Grade II-4 tumor, Forward | 59 | 59 | 61 | 61 | 59 | 52 | 52 | 59 | 59 | 44 | 59 |
| Grade II-4 tumor, Reverse | 52 | 52 | 28 | 27 | 38 | 28 | 43 | 43 | 28 | 36 | 59 |
