# Supplementary material for: First insight into the somatic mutation burden of neurofibromatosis type 2-associated grade I and grade II meningiomas: a case report comprehensive genomic study of two cranial meningiomas with vastly different clinical presentation
Source: BMC Cancer. 2017 Feb 13;17:127. doi: 10.1186/s12885-017-3127-6 (PMC5307647; doi:10.1186/s12885-017-3127-6)
Supplement: Additional file 2: — Losses, gains and regions with allelic imbalance in the grade I meningioma and all four fragments of the grade II meningioma. (PDF 173 kb) [file 12885_2017_3127_MOESM2_ESM.pdf]

Additional File 2. Losses, gains and regions with allelic imbalance in the grade I meningioma and all four fragments of the grade II meningioma

Region	Genomic Event	Sample Name	Participation
chr1:0-2,342,520	Allelic Imbalance	Grade1	Absent
chr1:0-2,342,520	Allelic Imbalance	Grade2-1	Absent
chr1:0-2,342,520	Allelic Imbalance	Grade2-2	Absent
chr1:0-2,342,520	Allelic Imbalance	Grade2-4	Absent
chr1:0-2,342,520	Allelic Imbalance	Grade2-3	Complete
chr1:1,239,637-1,296,372	Loss	Grade1	Absent
chr1:1,239,637-1,296,372	Loss	Grade2-1	Absent
chr1:1,239,637-1,296,372	Loss	Grade2-3	Absent
chr1:1,239,637-1,296,372	Loss	Grade2-4	Absent
chr1:1,239,637-1,296,372	Loss	Grade2-2	Complete
chr1:1,709,577-6,110,409	Loss	Grade1	Absent
chr1:1,709,577-6,110,409	Loss	Grade2-1	Partial
chr1:1,709,577-6,110,409	Loss	Grade2-2	Partial
chr1:1,709,577-6,110,409	Loss	Grade2-3	Partial
chr1:1,709,577-6,110,409	Loss	Grade2-4	Partial
chr1:3,372,743-6,110,409	Allelic Imbalance	Grade1	Absent
chr1:3,372,743-6,110,409	Allelic Imbalance	Grade2-1	Partial
chr1:3,372,743-6,110,409	Allelic Imbalance	Grade2-2	Partial
chr1:3,372,743-6,110,409	Allelic Imbalance	Grade2-3	Partial
chr1:3,372,743-6,110,409	Allelic Imbalance	Grade2-4	Partial
chr1:6,313,247-8,172,796	Allelic Imbalance	Grade1	Absent
chr1:6,313,247-8,172,796	Allelic Imbalance	Grade2-3	Absent
chr1:6,313,247-8,172,796	Allelic Imbalance	Grade2-4	Complete
chr1:6,313,247-8,172,796	Allelic Imbalance	Grade2-1	Partial
chr1:6,313,247-8,172,796	Allelic Imbalance	Grade2-2	Partial
chr1:6,313,247-8,181,652	Loss	Grade1	Absent
chr1:6,313,247-8,181,652	Loss	Grade2-1	Partial
chr1:6,313,247-8,181,652	Loss	Grade2-2	Partial
chr1:6,313,247-8,181,652	Loss	Grade2-3	Partial
chr1:6,313,247-8,181,652	Loss	Grade2-4	Partial
chr1:8,276,853-8,996,085	Loss	Grade1	Absent
chr1:8,276,853-8,996,085	Loss	Grade2-3	Absent
chr1:8,276,853-8,996,085	Loss	Grade2-1	Partial
chr1:8,276,853-8,996,085	Loss	Grade2-2	Partial
chr1:8,276,853-8,996,085	Loss	Grade2-4	Partial
chr1:9,063,095-9,135,973	Loss	Grade1	Absent
chr1:9,063,095-9,135,973	Loss	Grade2-2	Complete
chr1:9,063,095-9,135,973	Loss	Grade2-1	Partial
chr1:9,063,095-9,135,973	Loss	Grade2-3	Partial
chr1:9,063,095-9,135,973	Loss	Grade2-4	Partial
chr1:9,610,456-10,447,333	Loss	Grade1	Absent
chr1:9,610,456-10,447,333	Loss	Grade2-1	Partial

chr1:9,610,456-10,447,333	Loss	Grade2-2	Partial
chr1:9,610,456-10,447,333	Loss	Grade2-3	Partial
chr1:9,610,456-10,447,333	Loss	Grade2-4	Partial
chr1:10,976,898-11,506,265	Loss	Grade1	Absent
chr1:10,976,898-11,506,265	Loss	Grade2-1	Partial
chr1:10,976,898-11,506,265	Loss	Grade2-2	Partial
chr1:10,976,898-11,506,265	Loss	Grade2-3	Partial
chr1:10,976,898-11,506,265	Loss	Grade2-4	Partial
chr1:11,606,557-11,836,963	Loss	Grade1	Absent
chr1:11,606,557-11,836,963	Loss	Grade2-1	Partial
chr1:11,606,557-11,836,963	Loss	Grade2-2	Partial
chr1:11,606,557-11,836,963	Loss	Grade2-3	Partial
chr1:11,606,557-11,836,963	Loss	Grade2-4	Partial
chr1:12,164,074-13,830,696	Loss	Grade1	Absent
chr1:12,164,074-13,830,696	Loss	Grade2-1	Partial
chr1:12,164,074-13,830,696	Loss	Grade2-2	Partial
chr1:12,164,074-13,830,696	Loss	Grade2-3	Partial
chr1:12,164,074-13,830,696	Loss	Grade2-4	Partial
chr1:13,874,932-18,435,797	Allelic Imbalance	Grade1	Absent
chr1:13,874,932-18,435,797	Allelic Imbalance	Grade2-1	Partial
chr1:13,874,932-18,435,797	Allelic Imbalance	Grade2-2	Partial
chr1:13,874,932-18,435,797	Allelic Imbalance	Grade2-3	Partial
chr1:13,874,932-18,435,797	Allelic Imbalance	Grade2-4	Partial
chr1:13,874,932-18,438,295	Loss	Grade1	Absent
chr1:13,874,932-18,438,295	Loss	Grade2-1	Partial
chr1:13,874,932-18,438,295	Loss	Grade2-2	Partial
chr1:13,874,932-18,438,295	Loss	Grade2-3	Partial
chr1:13,874,932-18,438,295	Loss	Grade2-4	Partial
chr1:18,511,560-18,528,437	Loss	Grade1	Absent
chr1:18,511,560-18,528,437	Loss	Grade2-1	Absent
chr1:18,511,560-18,528,437	Loss	Grade2-3	Absent
chr1:18,511,560-18,528,437	Loss	Grade2-4	Absent
chr1:18,511,560-18,528,437	Loss	Grade2-2	Complete
chr1:18,583,459-20,882,863	Allelic Imbalance	Grade1	Absent
chr1:18,583,459-20,882,863	Allelic Imbalance	Grade2-1	Absent
chr1:18,583,459-20,882,863	Allelic Imbalance	Grade2-2	Absent
chr1:18,583,459-20,882,863	Allelic Imbalance	Grade2-3	Absent
chr1:18,583,459-20,882,863	Allelic Imbalance	Grade2-4	Complete
chr1:18,583,459-26,612,467	Loss	Grade1	Absent
chr1:18,583,459-26,612,467	Loss	Grade2-1	Partial
chr1:18,583,459-26,612,467	Loss	Grade2-2	Partial
chr1:18,583,459-26,612,467	Loss	Grade2-3	Partial
chr1:18,583,459-26,612,467	Loss	Grade2-4	Partial
chr1:20,924,563-26,612,467	Allelic Imbalance	Grade1	Absent
chr1:20,924,563-26,612,467	Allelic Imbalance	Grade2-2	Absent
chr1:20,924,563-26,612,467	Allelic Imbalance	Grade2-3	Absent
chr1:20,924,563-26,612,467	Allelic Imbalance	Grade2-4	Complete

chr1:20,924,563-26,612,467	Allelic Imbalance	Grade2-1	Partial
chr1:26,899,284-28,826,479	Loss	Grade1	Absent
chr1:26,899,284-28,826,479	Loss	Grade2-1	Absent
chr1:26,899,284-28,826,479	Loss	Grade2-4	Complete
chr1:26,899,284-28,826,479	Loss	Grade2-2	Partial
chr1:26,899,284-28,826,479	Loss	Grade2-3	Partial
chr1:29,743,519-31,258,543	Allelic Imbalance	Grade1	Absent
chr1:29,743,519-31,258,543	Allelic Imbalance	Grade2-4	Complete
chr1:29,743,519-31,258,543	Allelic Imbalance	Grade2-1	Partial
chr1:29,743,519-31,258,543	Allelic Imbalance	Grade2-2	Partial
chr1:29,743,519-31,258,543	Allelic Imbalance	Grade2-3	Partial
chr1:29,743,519-31,258,543	Loss	Grade1	Absent
chr1:29,743,519-31,258,543	Loss	Grade2-4	Complete
chr1:29,743,519-31,258,543	Loss	Grade2-1	Partial
chr1:29,743,519-31,258,543	Loss	Grade2-2	Partial
chr1:29,743,519-31,258,543	Loss	Grade2-3	Partial
chr1:31,316,754-36,734,509	Loss	Grade1	Absent
chr1:31,316,754-36,734,509	Loss	Grade2-4	Complete
chr1:31,316,754-36,734,509	Loss	Grade2-1	Partial
chr1:31,316,754-36,734,509	Loss	Grade2-2	Partial
chr1:31,316,754-36,734,509	Loss	Grade2-3	Partial
chr1:36,939,814-39,427,875	Allelic Imbalance	Grade1	Absent
chr1:36,939,814-39,427,875	Allelic Imbalance	Grade2-2	Absent
chr1:36,939,814-39,427,875	Allelic Imbalance	Grade2-3	Absent
chr1:36,939,814-39,427,875	Allelic Imbalance	Grade2-4	Absent
chr1:36,939,814-39,427,875	Allelic Imbalance	Grade2-1	Complete
chr1:36,939,814-41,087,251	Loss	Grade1	Absent
chr1:36,939,814-41,087,251	Loss	Grade2-1	Partial
chr1:36,939,814-41,087,251	Loss	Grade2-2	Partial
chr1:36,939,814-41,087,251	Loss	Grade2-3	Partial
chr1:36,939,814-41,087,251	Loss	Grade2-4	Partial
chr1:46,739,615-54,375,459	Loss	Grade1	Absent
chr1:46,739,615-54,375,459	Loss	Grade2-4	Complete
chr1:46,739,615-54,375,459	Loss	Grade2-1	Partial
chr1:46,739,615-54,375,459	Loss	Grade2-2	Partial
chr1:46,739,615-54,375,459	Loss	Grade2-3	Partial
chr1:52,398,520-54,305,759	Allelic Imbalance	Grade1	Absent
chr1:52,398,520-54,305,759	Allelic Imbalance	Grade2-1	Absent
chr1:52,398,520-54,305,759	Allelic Imbalance	Grade2-2	Absent
chr1:52,398,520-54,305,759	Allelic Imbalance	Grade2-4	Absent
chr1:52,398,520-54,305,759	Allelic Imbalance	Grade2-3	Complete
chr1:54,471,319-68,478,313	Allelic Imbalance	Grade1	Absent
chr1:54,471,319-68,478,313	Allelic Imbalance	Grade2-1	Partial
chr1:54,471,319-68,478,313	Allelic Imbalance	Grade2-2	Partial
chr1:54,471,319-68,478,313	Allelic Imbalance	Grade2-3	Partial
chr1:54,471,319-68,478,313	Allelic Imbalance	Grade2-4	Partial
chr1:54,471,319-68,499,957	Loss	Grade1	Absent

chr1:54,471,319-68,499,957	Loss	Grade2-4	Complete
chr1:54,471,319-68,499,957	Loss	Grade2-1	Partial
chr1:54,471,319-68,499,957	Loss	Grade2-2	Partial
chr1:54,471,319-68,499,957	Loss	Grade2-3	Partial
chr1:68,475,798-71,023,273	Allelic Loss	Grade1	Absent
chr1:68,475,798-71,023,273	Allelic Loss	Grade2-1	Complete
chr1:68,475,798-71,023,273	Allelic Loss	Grade2-2	Partial
chr1:68,475,798-71,023,273	Allelic Loss	Grade2-3	Partial
chr1:68,475,798-71,023,273	Allelic Loss	Grade2-4	Partial
chr1:71,005,838-71,511,289	Loss	Grade1	Absent
chr1:71,005,838-71,511,289	Loss	Grade2-2	Complete
chr1:71,005,838-71,511,289	Loss	Grade2-3	Complete
chr1:71,005,838-71,511,289	Loss	Grade2-4	Complete
chr1:71,005,838-71,511,289	Loss	Grade2-1	Partial
chr1:71,684,307-72,019,324	Loss	Grade1	Absent
chr1:71,684,307-72,019,324	Loss	Grade2-1	Complete
chr1:71,684,307-72,019,324	Loss	Grade2-2	Complete
chr1:71,684,307-72,019,324	Loss	Grade2-3	Complete
chr1:71,684,307-72,019,324	Loss	Grade2-4	Partial
chr1:76,113,826-76,336,750	Loss	Grade1	Absent
chr1:76,113,826-76,336,750	Loss	Grade2-1	Complete
chr1:76,113,826-76,336,750	Loss	Grade2-2	Complete
chr1:76,113,826-76,336,750	Loss	Grade2-3	Complete
chr1:76,113,826-76,336,750	Loss	Grade2-4	Complete
chr1:78,719,046-86,495,830	Allelic Imbalance	Grade1	Absent
chr1:78,719,046-86,495,830	Allelic Imbalance	Grade2-2	Complete
chr1:78,719,046-86,495,830	Allelic Imbalance	Grade2-1	Partial
chr1:78,719,046-86,495,830	Allelic Imbalance	Grade2-3	Partial
chr1:78,719,046-86,495,830	Allelic Imbalance	Grade2-4	Partial
chr1:78,719,046-86,495,830	Loss	Grade1	Absent
chr1:78,719,046-86,495,830	Loss	Grade2-2	Complete
chr1:78,719,046-86,495,830	Loss	Grade2-1	Partial
chr1:78,719,046-86,495,830	Loss	Grade2-3	Partial
chr1:78,719,046-86,495,830	Loss	Grade2-4	Partial
chr1:93,732,250-103,694,48	Allelic Imbalance	Grade1	Absent
chr1:93,732,250-103,694,48	Allelic Imbalance	Grade2-1	Absent
chr1:93,732,250-103,694,48	Allelic Imbalance	Grade2-3	Absent
chr1:93,732,250-103,694,48	Allelic Imbalance	Grade2-4	Absent
chr1:93,732,250-103,694,48	Allelic Imbalance	Grade2-2	Complete
chr1:93,732,250-103,694,48	Loss	Grade1	Absent
chr1:93,732,250-103,694,48	Loss	Grade2-2	Complete
chr1:93,732,250-103,694,48	Loss	Grade2-1	Partial
chr1:93,732,250-103,694,48	Loss	Grade2-3	Partial
chr1:93,732,250-103,694,48	Loss	Grade2-4	Partial
chr1:103,763,946-115,397,9	Allelic Imbalance	Grade1	Absent
chr1:103,763,946-115,397,9	Allelic Imbalance	Grade2-4	Complete
chr1:103,763,946-115,397,9	Allelic Imbalance	Grade2-1	Partial

chr1:103,763,946-115,397,9	Allelic Imbalance	Grade2-2	Partial
chr1:103,763,946-115,397,9	Allelic Imbalance	Grade2-3	Partial
chr1:103,763,946-115,401,9	Loss	Grade1	Absent
chr1:103,763,946-115,401,9	Loss	Grade2-1	Partial
chr1:103,763,946-115,401,9	Loss	Grade2-2	Partial
chr1:103,763,946-115,401,9	Loss	Grade2-3	Partial
chr1:103,763,946-115,401,9	Loss	Grade2-4	Partial
chr1:149,756,665-152,217,8	Allelic Imbalance	Grade1	Absent
chr1:149,756,665-152,217,8	Allelic Imbalance	Grade2-2	Complete
chr1:149,756,665-152,217,8	Allelic Imbalance	Grade2-1	Partial
chr1:149,756,665-152,217,8	Allelic Imbalance	Grade2-3	Partial
chr1:149,756,665-152,217,8	Allelic Imbalance	Grade2-4	Partial
chr1:149,756,665-152,217,8	Gain	Grade1	Absent
chr1:149,756,665-152,217,8	Gain	Grade2-2	Complete
chr1:149,756,665-152,217,8	Gain	Grade2-1	Partial
chr1:149,756,665-152,217,8	Gain	Grade2-3	Partial
chr1:149,756,665-152,217,8	Gain	Grade2-4	Partial
chr1:153,693,339-155,203,1	Allelic Imbalance	Grade1	Absent
chr1:153,693,339-155,203,1	Allelic Imbalance	Grade2-2	Complete
chr1:153,693,339-155,203,1	Allelic Imbalance	Grade2-1	Partial
chr1:153,693,339-155,203,1	Allelic Imbalance	Grade2-3	Partial
chr1:153,693,339-155,203,1	Allelic Imbalance	Grade2-4	Partial
chr1:153,693,339-155,203,1	Gain	Grade1	Absent
chr1:153,693,339-155,203,1	Gain	Grade2-2	Complete
chr1:153,693,339-155,203,1	Gain	Grade2-1	Partial
chr1:153,693,339-155,203,1	Gain	Grade2-3	Partial
chr1:153,693,339-155,203,1	Gain	Grade2-4	Partial
chr1:155,209,741-157,960,2	Gain	Grade1	Absent
chr1:155,209,741-157,960,2	Gain	Grade2-2	Complete
chr1:155,209,741-157,960,2	Gain	Grade2-1	Partial
chr1:155,209,741-157,960,2	Gain	Grade2-3	Partial
chr1:155,209,741-157,960,2	Gain	Grade2-4	Partial
chr1:155,209,741-158,735,7	Allelic Imbalance	Grade1	Absent
chr1:155,209,741-158,735,7	Allelic Imbalance	Grade2-1	Partial
chr1:155,209,741-158,735,7	Allelic Imbalance	Grade2-2	Partial
chr1:155,209,741-158,735,7	Allelic Imbalance	Grade2-3	Partial
chr1:155,209,741-158,735,7	Allelic Imbalance	Grade2-4	Partial
chr1:158,086,703-158,735,7	Gain	Grade1	Absent
chr1:158,086,703-158,735,7	Gain	Grade2-1	Partial
chr1:158,086,703-158,735,7	Gain	Grade2-2	Partial
chr1:158,086,703-158,735,7	Gain	Grade2-3	Partial
chr1:158,086,703-158,735,7	Gain	Grade2-4	Partial
chr1:158,747,432-164,879,3	Allelic Imbalance	Grade1	Absent
chr1:158,747,432-164,879,3	Allelic Imbalance	Grade2-2	Complete
chr1:158,747,432-164,879,3	Allelic Imbalance	Grade2-1	Partial
chr1:158,747,432-164,879,3	Allelic Imbalance	Grade2-3	Partial
chr1:158,747,432-164,879,3	Allelic Imbalance	Grade2-4	Partial

chr1:158,747,432-164,879,3	Gain	Grade1	Absent
chr1:158,747,432-164,879,3	Gain	Grade2-2	Complete
chr1:158,747,432-164,879,3	Gain	Grade2-1	Partial
chr1:158,747,432-164,879,3	Gain	Grade2-3	Partial
chr1:158,747,432-164,879,3	Gain	Grade2-4	Partial
chr1:165,034,548-165,341,3	Gain	Grade1	Absent
chr1:165,034,548-165,341,3	Gain	Grade2-2	Complete
chr1:165,034,548-165,341,3	Gain	Grade2-1	Partial
chr1:165,034,548-165,341,3	Gain	Grade2-3	Partial
chr1:165,034,548-165,341,3	Gain	Grade2-4	Partial
chr1:165,034,548-165,778,5	Allelic Imbalance	Grade1	Absent
chr1:165,034,548-165,778,5	Allelic Imbalance	Grade2-1	Complete
chr1:165,034,548-165,778,5	Allelic Imbalance	Grade2-3	Complete
chr1:165,034,548-165,778,5	Allelic Imbalance	Grade2-4	Complete
chr1:165,034,548-165,778,5	Allelic Imbalance	Grade2-2	Partial
chr1:165,560,963-165,778,5	Gain	Grade1	Absent
chr1:165,560,963-165,778,5	Gain	Grade2-1	Partial
chr1:165,560,963-165,778,5	Gain	Grade2-2	Partial
chr1:165,560,963-165,778,5	Gain	Grade2-3	Partial
chr1:165,560,963-165,778,5	Gain	Grade2-4	Partial
chr1:165,922,580-167,016,2	Gain	Grade1	Absent
chr1:165,922,580-167,016,2	Gain	Grade2-1	Partial
chr1:165,922,580-167,016,2	Gain	Grade2-2	Partial
chr1:165,922,580-167,016,2	Gain	Grade2-3	Partial
chr1:165,922,580-167,016,2	Gain	Grade2-4	Partial
chr1:167,157,499-175,090,2	Gain	Grade1	Absent
chr1:167,157,499-175,090,2	Gain	Grade2-1	Partial
chr1:167,157,499-175,090,2	Gain	Grade2-2	Partial
chr1:167,157,499-175,090,2	Gain	Grade2-3	Partial
chr1:167,157,499-175,090,2	Gain	Grade2-4	Partial
chr1:175,855,987-177,706,7	Gain	Grade1	Absent
chr1:175,855,987-177,706,7	Gain	Grade2-4	Complete
chr1:175,855,987-177,706,7	Gain	Grade2-1	Partial
chr1:175,855,987-177,706,7	Gain	Grade2-2	Partial
chr1:175,855,987-177,706,7	Gain	Grade2-3	Partial
chr1:177,813,426-180,097,1	Gain	Grade1	Absent
chr1:177,813,426-180,097,1	Gain	Grade2-1	Partial
chr1:177,813,426-180,097,1	Gain	Grade2-2	Partial
chr1:177,813,426-180,097,1	Gain	Grade2-3	Partial
chr1:177,813,426-180,097,1	Gain	Grade2-4	Partial
chr1:180,167,839-180,901,4	Gain	Grade1	Absent
chr1:180,167,839-180,901,4	Gain	Grade2-4	Complete
chr1:180,167,839-180,901,4	Gain	Grade2-1	Partial
chr1:180,167,839-180,901,4	Gain	Grade2-2	Partial
chr1:180,167,839-180,901,4	Gain	Grade2-3	Partial
chr1:181,111,459-181,405,3	Gain	Grade1	Absent
chr1:181,111,459-181,405,3	Gain	Grade2-1	Absent

chr1:181,111,459-181,405,3	Gain	Grade2-2	Partial
chr1:181,111,459-181,405,3	Gain	Grade2-3	Partial
chr1:181,111,459-181,405,3	Gain	Grade2-4	Partial
chr1:181,481,984-181,712,3	Gain	Grade1	Absent
chr1:181,481,984-181,712,3	Gain	Grade2-1	Partial
chr1:181,481,984-181,712,3	Gain	Grade2-2	Partial
chr1:181,481,984-181,712,3	Gain	Grade2-3	Partial
chr1:181,481,984-181,712,3	Gain	Grade2-4	Partial
chr1:181,842,663-204,999,5	Gain	Grade1	Absent
chr1:181,842,663-204,999,5	Gain	Grade2-1	Partial
chr1:181,842,663-204,999,5	Gain	Grade2-2	Partial
chr1:181,842,663-204,999,5	Gain	Grade2-3	Partial
chr1:181,842,663-204,999,5	Gain	Grade2-4	Partial
chr1:165,922,580-226,032,6	Allelic Imbalance	Grade1	Absent
chr1:165,922,580-226,032,6	Allelic Imbalance	Grade2-1	Partial
chr1:165,922,580-226,032,6	Allelic Imbalance	Grade2-2	Partial
chr1:165,922,580-226,032,6	Allelic Imbalance	Grade2-3	Partial
chr1:165,922,580-226,032,6	Allelic Imbalance	Grade2-4	Partial
chr1:205,053,666-205,763,5	Gain	Grade1	Absent
chr1:205,053,666-205,763,5	Gain	Grade2-1	Complete
chr1:205,053,666-205,763,5	Gain	Grade2-3	Complete
chr1:205,053,666-205,763,5	Gain	Grade2-2	Partial
chr1:205,053,666-205,763,5	Gain	Grade2-4	Partial
chr1:205,898,895-206,233,0	Gain	Grade1	Absent
chr1:205,898,895-206,233,0	Gain	Grade2-1	Absent
chr1:205,898,895-206,233,0	Gain	Grade2-3	Absent
chr1:205,898,895-206,233,0	Gain	Grade2-2	Complete
chr1:205,898,895-206,233,0	Gain	Grade2-4	Partial
chr1:206,616,227-207,038,0	Gain	Grade1	Absent
chr1:206,616,227-207,038,0	Gain	Grade2-3	Absent
chr1:206,616,227-207,038,0	Gain	Grade2-4	Complete
chr1:206,616,227-207,038,0	Gain	Grade2-1	Partial
chr1:206,616,227-207,038,0	Gain	Grade2-2	Partial
chr1:207,195,651-210,724,6	Gain	Grade1	Absent
chr1:207,195,651-210,724,6	Gain	Grade2-1	Partial
chr1:207,195,651-210,724,6	Gain	Grade2-2	Partial
chr1:207,195,651-210,724,6	Gain	Grade2-3	Partial
chr1:207,195,651-210,724,6	Gain	Grade2-4	Partial
chr1:210,785,771-213,488,1	Gain	Grade1	Absent
chr1:210,785,771-213,488,1	Gain	Grade2-1	Partial
chr1:210,785,771-213,488,1	Gain	Grade2-2	Partial
chr1:210,785,771-213,488,1	Gain	Grade2-3	Partial
chr1:210,785,771-213,488,1	Gain	Grade2-4	Partial
chr1:213,627,727-213,941,9	Gain	Grade1	Absent
chr1:213,627,727-213,941,9	Gain	Grade2-4	Complete
chr1:213,627,727-213,941,9	Gain	Grade2-1	Partial
chr1:213,627,727-213,941,9	Gain	Grade2-2	Partial

chr1:213,627,727-213,941,9	Gain	Grade2-3	Partial
chr1:214,084,263-220,868,2	Gain	Grade1	Absent
chr1:214,084,263-220,868,2	Gain	Grade2-4	Complete
chr1:214,084,263-220,868,2	Gain	Grade2-1	Partial
chr1:214,084,263-220,868,2	Gain	Grade2-2	Partial
chr1:214,084,263-220,868,2	Gain	Grade2-3	Partial
chr1:221,236,220-224,863,8	Gain	Grade1	Absent
chr1:221,236,220-224,863,8	Gain	Grade2-1	Partial
chr1:221,236,220-224,863,8	Gain	Grade2-2	Partial
chr1:221,236,220-224,863,8	Gain	Grade2-3	Partial
chr1:221,236,220-224,863,8	Gain	Grade2-4	Partial
chr1:224,914,179-226,032,6	Gain	Grade1	Absent
chr1:224,914,179-226,032,6	Gain	Grade2-1	Partial
chr1:224,914,179-226,032,6	Gain	Grade2-2	Partial
chr1:224,914,179-226,032,6	Gain	Grade2-3	Partial
chr1:224,914,179-226,032,6	Gain	Grade2-4	Partial
chr1:226,073,063-227,336,9	Allelic Imbalance	Grade1	Absent
chr1:226,073,063-227,336,9	Allelic Imbalance	Grade2-4	Complete
chr1:226,073,063-227,336,9	Allelic Imbalance	Grade2-1	Partial
chr1:226,073,063-227,336,9	Allelic Imbalance	Grade2-2	Partial
chr1:226,073,063-227,336,9	Allelic Imbalance	Grade2-3	Partial
chr1:226,073,063-227,336,9	Gain	Grade1	Absent
chr1:226,073,063-227,336,9	Gain	Grade2-4	Complete
chr1:226,073,063-227,336,9	Gain	Grade2-1	Partial
chr1:226,073,063-227,336,9	Gain	Grade2-2	Partial
chr1:226,073,063-227,336,9	Gain	Grade2-3	Partial
chr2:25,286,655-27,427,390	Loss	Grade1	Absent
chr2:25,286,655-27,427,390	Loss	Grade2-1	Partial
chr2:25,286,655-27,427,390	Loss	Grade2-2	Partial
chr2:25,286,655-27,427,390	Loss	Grade2-3	Partial
chr2:25,286,655-27,427,390	Loss	Grade2-4	Partial
chr2:141,344,553-141,948,8	Loss	Grade1	Absent
chr2:141,344,553-141,948,8	Loss	Grade2-3	Complete
chr2:141,344,553-141,948,8	Loss	Grade2-4	Complete
chr2:141,344,553-141,948,8	Loss	Grade2-1	Partial
chr2:141,344,553-141,948,8	Loss	Grade2-2	Partial
chr2:142,066,896-142,114,0	Loss	Grade1	Absent
chr2:142,066,896-142,114,0	Loss	Grade2-1	Absent
chr2:142,066,896-142,114,0	Loss	Grade2-3	Absent
chr2:142,066,896-142,114,0	Loss	Grade2-4	Complete
chr2:142,066,896-142,114,0	Loss	Grade2-2	Partial
chr3:60,533,831-60,552,706	Loss	Grade1	Absent
chr3:60,533,831-60,552,706	Loss	Grade2-1	Absent
chr3:60,533,831-60,552,706	Loss	Grade2-3	Absent
chr3:60,533,831-60,552,706	Loss	Grade2-2	Complete
chr3:60,533,831-60,552,706	Loss	Grade2-4	Partial
chr3:60,729,820-60,775,714	Gain	Grade1	Absent

chr3:60,729,820-60,775,714	Gain	Grade2-2	Absent
chr3:60,729,820-60,775,714	Gain	Grade2-1	Partial
chr3:60,729,820-60,775,714	Gain	Grade2-3	Partial
chr3:60,729,820-60,775,714	Gain	Grade2-4	Partial
chr3:174,219,266-174,359,1	Loss	Grade1	Absent
chr3:174,219,266-174,359,1	Loss	Grade2-4	Complete
chr3:174,219,266-174,359,1	Loss	Grade2-1	Partial
chr3:174,219,266-174,359,1	Loss	Grade2-2	Partial
chr3:174,219,266-174,359,1	Loss	Grade2-3	Partial
chr6:63,926,951-64,934,998	Loss	Grade1	Absent
chr6:63,926,951-64,934,998	Loss	Grade2-1	Complete
chr6:63,926,951-64,934,998	Loss	Grade2-2	Complete
chr6:63,926,951-64,934,998	Loss	Grade2-3	Complete
chr6:63,926,951-64,934,998	Loss	Grade2-4	Complete
chr6:70,142,826-134,466,51	Loss	Grade1	Absent
chr6:70,142,826-134,466,51	Loss	Grade2-2	Complete
chr6:70,142,826-134,466,51	Loss	Grade2-1	Partial
chr6:70,142,826-134,466,51	Loss	Grade2-3	Partial
chr6:70,142,826-134,466,51	Loss	Grade2-4	Partial
chr6:134,521,112-167,155,0	Loss	Grade1	Absent
chr6:134,521,112-167,155,0	Loss	Grade2-1	Partial
chr6:134,521,112-167,155,0	Loss	Grade2-2	Partial
chr6:134,521,112-167,155,0	Loss	Grade2-3	Partial
chr6:134,521,112-167,155,0	Loss	Grade2-4	Partial
chr6:151,674,459-157,877,9	Allelic Imbalance	Grade1	Absent
chr6:151,674,459-157,877,9	Allelic Imbalance	Grade2-2	Absent
chr6:151,674,459-157,877,9	Allelic Imbalance	Grade2-4	Absent
chr6:151,674,459-157,877,9	Allelic Imbalance	Grade2-3	Complete
chr6:151,674,459-157,877,9	Allelic Imbalance	Grade2-1	Partial
chr6:158,102,055-167,155,0	Allelic Imbalance	Grade1	Absent
chr6:158,102,055-167,155,0	Allelic Imbalance	Grade2-2	Complete
chr6:158,102,055-167,155,0	Allelic Imbalance	Grade2-1	Partial
chr6:158,102,055-167,155,0	Allelic Imbalance	Grade2-3	Partial
chr6:158,102,055-167,155,0	Allelic Imbalance	Grade2-4	Partial
chr6:167,190,221-170,445,4	Loss	Grade1	Absent
chr6:167,190,221-170,445,4	Loss	Grade2-1	Partial
chr6:167,190,221-170,445,4	Loss	Grade2-2	Partial
chr6:167,190,221-170,445,4	Loss	Grade2-3	Partial
chr6:167,190,221-170,445,4	Loss	Grade2-4	Partial
chr6:167,197,802-170,445,4	Allelic Imbalance	Grade1	Absent
chr6:167,197,802-170,445,4	Allelic Imbalance	Grade2-1	Partial
chr6:167,197,802-170,445,4	Allelic Imbalance	Grade2-2	Partial
chr6:167,197,802-170,445,4	Allelic Imbalance	Grade2-3	Partial
chr6:167,197,802-170,445,4	Allelic Imbalance	Grade2-4	Partial
chr6:170,499,358-171,115,0	Loss	Grade1	Absent
chr6:170,499,358-171,115,0	Loss	Grade2-3	Complete
chr6:170,499,358-171,115,0	Loss	Grade2-1	Partial

chr6:170,499,358-171,115,0	Loss	Grade2-2	Partial
chr6:170,499,358-171,115,0	Loss	Grade2-4	Partial
chr7:38,294,904-38,384,590	Gain	Grade2-4	Complete
chr7:38,294,904-38,384,590	Gain	Grade1	Partial
chr7:38,294,904-38,384,590	Gain	Grade2-1	Partial
chr7:38,294,904-38,384,590	Gain	Grade2-2	Partial
chr7:38,294,904-38,384,590	Gain	Grade2-3	Partial
chr7:61,074,194-62,368,143	Gain	Grade1	Absent
chr7:61,074,194-62,368,143	Gain	Grade2-4	Complete
chr7:61,074,194-62,368,143	Gain	Grade2-1	Partial
chr7:61,074,194-62,368,143	Gain	Grade2-2	Partial
chr7:61,074,194-62,368,143	Gain	Grade2-3	Partial
chr8:3,679,475-3,701,441	Loss	Grade2-1	Absent
chr8:3,679,475-3,701,441	Loss	Grade2-2	Absent
chr8:3,679,475-3,701,441	Loss	Grade2-3	Absent
chr8:3,679,475-3,701,441	Loss	Grade2-4	Absent
chr8:3,679,475-3,701,441	Loss	Grade1	Complete
chr11:326,567-939,530	Loss	Grade1	Absent
chr11:326,567-939,530	Loss	Grade2-1	Partial
chr11:326,567-939,530	Loss	Grade2-2	Partial
chr11:326,567-939,530	Loss	Grade2-3	Partial
chr11:326,567-939,530	Loss	Grade2-4	Partial
chr11:1,033,174-1,254,645	Loss	Grade1	Absent
chr11:1,033,174-1,254,645	Loss	Grade2-1	Absent
chr11:1,033,174-1,254,645	Loss	Grade2-2	Absent
chr11:1,033,174-1,254,645	Loss	Grade2-3	Absent
chr11:1,033,174-1,254,645	Loss	Grade2-4	Complete
chr11:1,413,671-1,644,910	Loss	Grade1	Absent
chr11:1,413,671-1,644,910	Loss	Grade2-1	Complete
chr11:1,413,671-1,644,910	Loss	Grade2-2	Complete
chr11:1,413,671-1,644,910	Loss	Grade2-3	Complete
chr11:1,413,671-1,644,910	Loss	Grade2-4	Complete
chr11:1,699,159-3,033,703	Allelic Imbalance	Grade1	Absent
chr11:1,699,159-3,033,703	Allelic Imbalance	Grade2-4	Complete
chr11:1,699,159-3,033,703	Allelic Imbalance	Grade2-1	Partial
chr11:1,699,159-3,033,703	Allelic Imbalance	Grade2-2	Partial
chr11:1,699,159-3,033,703	Allelic Imbalance	Grade2-3	Partial
chr11:1,699,159-3,033,703	Loss	Grade1	Absent
chr11:1,699,159-3,033,703	Loss	Grade2-4	Complete
chr11:1,699,159-3,033,703	Loss	Grade2-1	Partial
chr11:1,699,159-3,033,703	Loss	Grade2-2	Partial
chr11:1,699,159-3,033,703	Loss	Grade2-3	Partial
chr11:3,116,008-9,536,682	Loss	Grade1	Absent
chr11:3,116,008-9,536,682	Loss	Grade2-1	Partial
chr11:3,116,008-9,536,682	Loss	Grade2-2	Partial
chr11:3,116,008-9,536,682	Loss	Grade2-3	Partial
chr11:3,116,008-9,536,682	Loss	Grade2-4	Partial

chr11:3,116,352-9,536,682	Allelic Imbalance	Grade1	Absent
chr11:3,116,352-9,536,682	Allelic Imbalance	Grade2-4	Complete
chr11:3,116,352-9,536,682	Allelic Imbalance	Grade2-1	Partial
chr11:3,116,352-9,536,682	Allelic Imbalance	Grade2-2	Partial
chr11:3,116,352-9,536,682	Allelic Imbalance	Grade2-3	Partial
chr12:0-6,132,603	Loss	Grade1	Absent
chr12:0-6,132,603	Loss	Grade2-1	Partial
chr12:0-6,132,603	Loss	Grade2-2	Partial
chr12:0-6,132,603	Loss	Grade2-3	Partial
chr12:0-6,132,603	Loss	Grade2-4	Partial
chr12:373,761-32,583,652	Allelic Imbalance	Grade1	Absent
chr12:373,761-32,583,652	Allelic Imbalance	Grade2-1	Partial
chr12:373,761-32,583,652	Allelic Imbalance	Grade2-2	Partial
chr12:373,761-32,583,652	Allelic Imbalance	Grade2-3	Partial
chr12:373,761-32,583,652	Allelic Imbalance	Grade2-4	Partial
chr12:7,054,129-32,583,652	Loss	Grade1	Absent
chr12:7,054,129-32,583,652	Loss	Grade2-1	Partial
chr12:7,054,129-32,583,652	Loss	Grade2-2	Partial
chr12:7,054,129-32,583,652	Loss	Grade2-3	Partial
chr12:7,054,129-32,583,652	Loss	Grade2-4	Partial
chr12:37,876,400-38,211,89	Gain	Grade1	Absent
chr12:37,876,400-38,211,89	Gain	Grade2-1	Absent
chr12:37,876,400-38,211,89	Gain	Grade2-2	Absent
chr12:37,876,400-38,211,89	Gain	Grade2-4	Absent
chr12:37,876,400-38,211,89	Gain	Grade2-3	Complete
chr14:22,499,539-22,968,45	Gain	Grade1	Complete
chr14:22,499,539-22,968,45	Gain	Grade2-1	Complete
chr14:22,499,539-22,968,45	Gain	Grade2-2	Complete
chr14:22,499,539-22,968,45	Gain	Grade2-4	Complete
chr14:22,499,539-22,968,45	Gain	Grade2-3	Partial
chr14:82,307,322-82,497,14	Loss	Grade1	Absent
chr14:82,307,322-82,497,14	Loss	Grade2-2	Complete
chr14:82,307,322-82,497,14	Loss	Grade2-1	Partial
chr14:82,307,322-82,497,14	Loss	Grade2-3	Partial
chr14:82,307,322-82,497,14	Loss	Grade2-4	Partial
chr18:76,945,094-77,138,05	Loss	Grade1	Absent
chr18:76,945,094-77,138,05	Loss	Grade2-1	Partial
chr18:76,945,094-77,138,05	Loss	Grade2-2	Partial
chr18:76,945,094-77,138,05	Loss	Grade2-3	Partial
chr18:76,945,094-77,138,05	Loss	Grade2-4	Partial
chr18:77,805,777-78,077,24	Loss	Grade1	Absent
chr18:77,805,777-78,077,24	Loss	Grade2-2	Complete
chr18:77,805,777-78,077,24	Loss	Grade2-4	Complete
chr18:77,805,777-78,077,24	Loss	Grade2-1	Partial
chr18:77,805,777-78,077,24	Loss	Grade2-3	Partial
chr22:16,197,021-17,437,07	Loss	Grade2-1	Absent
chr22:16,197,021-17,437,07	Loss	Grade2-2	Absent

chr22:16,197,021-17,437,07	Loss	Grade2-3	Absent
chr22:16,197,021-17,437,07	Loss	Grade2-4	Absent
chr22:16,197,021-17,437,07	Loss	Grade1	Complete
chr22:17,437,078-19,426,86	Allelic Imbalance	Grade2-1	Absent
chr22:17,437,078-19,426,86	Allelic Imbalance	Grade2-2	Absent
chr22:17,437,078-19,426,86	Allelic Imbalance	Grade2-3	Absent
chr22:17,437,078-19,426,86	Allelic Imbalance	Grade2-4	Absent
chr22:17,437,078-19,426,86	Allelic Imbalance	Grade1	Complete
chr22:19,426,861-19,585,25	Loss	Grade2-1	Absent
chr22:19,426,861-19,585,25	Loss	Grade2-2	Absent
chr22:19,426,861-19,585,25	Loss	Grade2-3	Absent
chr22:19,426,861-19,585,25	Loss	Grade2-4	Absent
chr22:19,426,861-19,585,25	Loss	Grade1	Complete
chr22:19,585,254-19,870,48	Allelic Imbalance	Grade2-1	Absent
chr22:19,585,254-19,870,48	Allelic Imbalance	Grade2-2	Absent
chr22:19,585,254-19,870,48	Allelic Imbalance	Grade2-3	Absent
chr22:19,585,254-19,870,48	Allelic Imbalance	Grade2-4	Absent
chr22:19,585,254-19,870,48	Allelic Imbalance	Grade1	Complete
chr22:19,870,489-19,949,05	Loss	Grade2-1	Absent
chr22:19,870,489-19,949,05	Loss	Grade2-2	Absent
chr22:19,870,489-19,949,05	Loss	Grade2-3	Absent
chr22:19,870,489-19,949,05	Loss	Grade2-4	Absent
chr22:19,870,489-19,949,05	Loss	Grade1	Complete
chr22:19,974,324-20,009,90	Loss	Grade2-1	Absent
chr22:19,974,324-20,009,90	Loss	Grade2-2	Absent
chr22:19,974,324-20,009,90	Loss	Grade2-3	Absent
chr22:19,974,324-20,009,90	Loss	Grade2-4	Absent
chr22:19,974,324-20,009,90	Loss	Grade1	Complete
chr22:20,852,369-20,950,40	Loss	Grade2-1	Absent
chr22:20,852,369-20,950,40	Loss	Grade2-2	Absent
chr22:20,852,369-20,950,40	Loss	Grade2-3	Absent
chr22:20,852,369-20,950,40	Loss	Grade2-4	Absent
chr22:20,852,369-20,950,40	Loss	Grade1	Complete
chr22:21,421,264-21,975,12	Loss	Grade2-1	Absent
chr22:21,421,264-21,975,12	Loss	Grade2-2	Absent
chr22:21,421,264-21,975,12	Loss	Grade2-3	Absent
chr22:21,421,264-21,975,12	Loss	Grade2-4	Absent
chr22:21,421,264-21,975,12	Loss	Grade1	Complete
chr22:22,244,707-23,633,54	Allelic Imbalance	Grade2-1	Absent
chr22:22,244,707-23,633,54	Allelic Imbalance	Grade2-2	Absent
chr22:22,244,707-23,633,54	Allelic Imbalance	Grade2-3	Absent
chr22:22,244,707-23,633,54	Allelic Imbalance	Grade2-4	Absent
chr22:22,244,707-23,633,54	Allelic Imbalance	Grade1	Complete
chr22:22,244,707-23,633,54	Loss	Grade2-1	Absent
chr22:22,244,707-23,633,54	Loss	Grade2-2	Absent
chr22:22,244,707-23,633,54	Loss	Grade2-3	Absent
chr22:22,244,707-23,633,54	Loss	Grade2-4	Absent

chr22:22,244,707-23,633,54	Loss	Grade1	Complete
chr22:23,701,151-25,377,77	Loss	Grade1	Partial
chr22:23,701,151-25,377,77	Loss	Grade2-1	Partial
chr22:23,701,151-25,377,77	Loss	Grade2-2	Partial
chr22:23,701,151-25,377,77	Loss	Grade2-3	Partial
chr22:23,701,151-25,377,77	Loss	Grade2-4	Partial
chr22:23,928,706-28,100,48	Allelic Imbalance	Grade2-1	Absent
chr22:23,928,706-28,100,48	Allelic Imbalance	Grade1	Partial
chr22:23,928,706-28,100,48	Allelic Imbalance	Grade2-2	Partial
chr22:23,928,706-28,100,48	Allelic Imbalance	Grade2-3	Partial
chr22:23,928,706-28,100,48	Allelic Imbalance	Grade2-4	Partial
chr22:25,585,854-28,100,48	Loss	Grade1	Partial
chr22:25,585,854-28,100,48	Loss	Grade2-1	Partial
chr22:25,585,854-28,100,48	Loss	Grade2-2	Partial
chr22:25,585,854-28,100,48	Loss	Grade2-3	Partial
chr22:25,585,854-28,100,48	Loss	Grade2-4	Partial
chr22:28,129,263-29,690,56	Loss	Grade1	Partial
chr22:28,129,263-29,690,56	Loss	Grade2-1	Partial
chr22:28,129,263-29,690,56	Loss	Grade2-2	Partial
chr22:28,129,263-29,690,56	Loss	Grade2-3	Partial
chr22:28,129,263-29,690,56	Loss	Grade2-4	Partial
chr22:29,790,127-30,689,33	Loss	Grade1	Partial
chr22:29,790,127-30,689,33	Loss	Grade2-1	Partial
chr22:29,790,127-30,689,33	Loss	Grade2-2	Partial
chr22:29,790,127-30,689,33	Loss	Grade2-3	Partial
chr22:29,790,127-30,689,33	Loss	Grade2-4	Partial
chr22:30,163,739-30,957,12	Allelic Imbalance	Grade2-1	Absent
chr22:30,163,739-30,957,12	Allelic Imbalance	Grade2-2	Absent
chr22:30,163,739-30,957,12	Allelic Imbalance	Grade2-3	Absent
chr22:30,163,739-30,957,12	Allelic Imbalance	Grade2-4	Absent
chr22:30,163,739-30,957,12	Allelic Imbalance	Grade1	Complete
chr22:30,763,296-32,011,22	Loss	Grade2-4	Complete
chr22:30,763,296-32,011,22	Loss	Grade1	Partial
chr22:30,763,296-32,011,22	Loss	Grade2-1	Partial
chr22:30,763,296-32,011,22	Loss	Grade2-2	Partial
chr22:30,763,296-32,011,22	Loss	Grade2-3	Partial
chr22:32,119,208-36,653,10	Loss	Grade2-4	Complete
chr22:32,119,208-36,653,10	Loss	Grade1	Partial
chr22:32,119,208-36,653,10	Loss	Grade2-1	Partial
chr22:32,119,208-36,653,10	Loss	Grade2-2	Partial
chr22:32,119,208-36,653,10	Loss	Grade2-3	Partial
chr22:31,770,014-40,029,00	Allelic Imbalance	Grade1	Partial
chr22:31,770,014-40,029,00	Allelic Imbalance	Grade2-1	Partial
chr22:31,770,014-40,029,00	Allelic Imbalance	Grade2-2	Partial
chr22:31,770,014-40,029,00	Allelic Imbalance	Grade2-3	Partial
chr22:31,770,014-40,029,00	Allelic Imbalance	Grade2-4	Partial
chr22:37,255,386-37,958,74	Loss	Grade2-4	Complete

chr22:37,255,386-37,958,74	Loss	Grade1	Partial
chr22:37,255,386-37,958,74	Loss	Grade2-1	Partial
chr22:37,255,386-37,958,74	Loss	Grade2-2	Partial
chr22:37,255,386-37,958,74	Loss	Grade2-3	Partial
chr22:38,699,380-39,430,00	Loss	Grade1	Absent
chr22:38,699,380-39,430,00	Loss	Grade2-1	Partial
chr22:38,699,380-39,430,00	Loss	Grade2-2	Partial
chr22:38,699,380-39,430,00	Loss	Grade2-3	Partial
chr22:38,699,380-39,430,00	Loss	Grade2-4	Partial
chr22:39,778,859-41,345,59	Loss	Grade2-4	Complete
chr22:39,778,859-41,345,59	Loss	Grade1	Partial
chr22:39,778,859-41,345,59	Loss	Grade2-1	Partial
chr22:39,778,859-41,345,59	Loss	Grade2-2	Partial
chr22:39,778,859-41,345,59	Loss	Grade2-3	Partial
chr22:40,931,121-42,689,98	Allelic Imbalance	Grade2-1	Absent
chr22:40,931,121-42,689,98	Allelic Imbalance	Grade2-2	Absent
chr22:40,931,121-42,689,98	Allelic Imbalance	Grade2-3	Absent
chr22:40,931,121-42,689,98	Allelic Imbalance	Grade2-4	Absent
chr22:40,931,121-42,689,98	Allelic Imbalance	Grade1	Complete
chr22:41,792,686-43,459,82	Loss	Grade1	Partial
chr22:41,792,686-43,459,82	Loss	Grade2-1	Partial
chr22:41,792,686-43,459,82	Loss	Grade2-2	Partial
chr22:41,792,686-43,459,82	Loss	Grade2-3	Partial
chr22:41,792,686-43,459,82	Loss	Grade2-4	Partial
chr22:42,819,631-44,270,19	Allelic Imbalance	Grade2-1	Absent
chr22:42,819,631-44,270,19	Allelic Imbalance	Grade2-2	Absent
chr22:42,819,631-44,270,19	Allelic Imbalance	Grade2-3	Absent
chr22:42,819,631-44,270,19	Allelic Imbalance	Grade2-4	Absent
chr22:42,819,631-44,270,19	Allelic Imbalance	Grade1	Complete
chr22:43,768,475-44,340,06	Loss	Grade2-4	Complete
chr22:43,768,475-44,340,06	Loss	Grade1	Partial
chr22:43,768,475-44,340,06	Loss	Grade2-1	Partial
chr22:43,768,475-44,340,06	Loss	Grade2-2	Partial
chr22:43,768,475-44,340,06	Loss	Grade2-3	Partial
chr22:44,423,373-44,494,44	Loss	Grade1	Absent
chr22:44,423,373-44,494,44	Loss	Grade2-1	Absent
chr22:44,423,373-44,494,44	Loss	Grade2-2	Absent
chr22:44,423,373-44,494,44	Loss	Grade2-3	Absent
chr22:44,423,373-44,494,44	Loss	Grade2-4	Complete
chr22:44,525,653-44,576,39	Loss	Grade1	Absent
chr22:44,525,653-44,576,39	Loss	Grade2-4	Absent
chr22:44,525,653-44,576,39	Loss	Grade2-2	Complete
chr22:44,525,653-44,576,39	Loss	Grade2-1	Partial
chr22:44,525,653-44,576,39	Loss	Grade2-3	Partial
chr22:44,649,410-45,586,88	Loss	Grade1	Partial
chr22:44,649,410-45,586,88	Loss	Grade2-1	Partial
chr22:44,649,410-45,586,88	Loss	Grade2-2	Partial

chr22:44,649,410-45,586,88	Loss	Grade2-3	Partial
chr22:44,649,410-45,586,88	Loss	Grade2-4	Partial
chr22:44,324,176-46,044,68	Allelic Imbalance	Grade1	Partial
chr22:44,324,176-46,044,68	Allelic Imbalance	Grade2-1	Partial
chr22:44,324,176-46,044,68	Allelic Imbalance	Grade2-2	Partial
chr22:44,324,176-46,044,68	Allelic Imbalance	Grade2-3	Partial
chr22:44,324,176-46,044,68	Allelic Imbalance	Grade2-4	Partial
chr22:45,821,913-45,896,05	Loss	Grade1	Absent
chr22:45,821,913-45,896,05	Loss	Grade2-2	Absent
chr22:45,821,913-45,896,05	Loss	Grade2-3	Absent
chr22:45,821,913-45,896,05	Loss	Grade2-4	Absent
chr22:45,821,913-45,896,05	Loss	Grade2-1	Complete
chr22:45,985,993-46,269,99	Loss	Grade1	Partial
chr22:45,985,993-46,269,99	Loss	Grade2-1	Partial
chr22:45,985,993-46,269,99	Loss	Grade2-2	Partial
chr22:45,985,993-46,269,99	Loss	Grade2-3	Partial
chr22:45,985,993-46,269,99	Loss	Grade2-4	Partial
chr22:46,355,329-46,908,39	Loss	Grade1	Partial
chr22:46,355,329-46,908,39	Loss	Grade2-1	Partial
chr22:46,355,329-46,908,39	Loss	Grade2-2	Partial
chr22:46,355,329-46,908,39	Loss	Grade2-3	Partial
chr22:46,355,329-46,908,39	Loss	Grade2-4	Partial
chr22:46,989,960-47,116,36	Loss	Grade1	Absent
chr22:46,989,960-47,116,36	Loss	Grade2-1	Absent
chr22:46,989,960-47,116,36	Loss	Grade2-4	Absent
chr22:46,989,960-47,116,36	Loss	Grade2-2	Complete
chr22:46,989,960-47,116,36	Loss	Grade2-3	Partial
chr22:47,344,809-50,084,58	Loss	Grade1	Partial
chr22:47,344,809-50,084,58	Loss	Grade2-1	Partial
chr22:47,344,809-50,084,58	Loss	Grade2-2	Partial
chr22:47,344,809-50,084,58	Loss	Grade2-3	Partial
chr22:47,344,809-50,084,58	Loss	Grade2-4	Partial
chr22:46,647,940-51,025,41	Allelic Imbalance	Grade1	Partial
chr22:46,647,940-51,025,41	Allelic Imbalance	Grade2-1	Partial
chr22:46,647,940-51,025,41	Allelic Imbalance	Grade2-2	Partial
chr22:46,647,940-51,025,41	Allelic Imbalance	Grade2-3	Partial
chr22:46,647,940-51,025,41	Allelic Imbalance	Grade2-4	Partial
chr22:50,982,436-51,065,07	Loss	Grade2-2	Absent
chr22:50,982,436-51,065,07	Loss	Grade2-3	Absent
chr22:50,982,436-51,065,07	Loss	Grade2-4	Complete
chr22:50,982,436-51,065,07	Loss	Grade1	Partial
chr22:50,982,436-51,065,07	Loss	Grade2-1	Partial
chr22:51,143,227-51,304,56	Loss	Grade1	Absent
chr22:51,143,227-51,304,56	Loss	Grade2-2	Absent
chr22:51,143,227-51,304,56	Loss	Grade2-3	Absent
chr22:51,143,227-51,304,56	Loss	Grade2-4	Absent
chr22:51,143,227-51,304,56	Loss	Grade2-1	Complete

chrX:32,737,942-33,582,89[illegible]	Loss	Grade1	Absent
chrX:32,737,942-33,582,89[illegible]	Loss	Grade2-1	Complete
chrX:32,737,942-33,582,89[illegible]	Loss	Grade2-3	Complete
chrX:32,737,942-33,582,89[illegible]	Loss	Grade2-2	Partial
chrX:32,737,942-33,582,89[illegible]	Loss	Grade2-4	Partial
