# Supplementary material for: First insight into the somatic mutation burden of neurofibromatosis type 2-associated grade I and grade II meningiomas: a case report comprehensive genomic study of two cranial meningiomas with vastly different clinical presentation
Source: BMC Cancer. 2017 Feb 13;17:127. doi: 10.1186/s12885-017-3127-6 (PMC5307647; doi:10.1186/s12885-017-3127-6)
Supplement: Additional file 3: — Genes affected by gains and losses in the grade II meningioma. (PDF 884 kb) [file 12885_2017_3127_MOESM3_ESM.pdf]

Additional File 3. Genes, affected by gains and losses in the grade II meningioma.

Gene Symbol	Chromosome	Start	End	Length	Gene Name
PEX10	chr1	2336240	2344010	7771	peroxisomal biogenesis factor 10
PLCH2	chr1	2407753	2436969	29217	phospholipase C, eta 2
ACTRT2	chr1	2938045	2939467	1423	actin-related protein T2
FLJ42875	chr1	2976180	2980350	4171	
LINC00982	chr1	2976180	2984289	8110	long intergenic non-protein coding RNA 982
FLJ42875	chr1	2980635	2984289	3655	
MIR4251	chr1	3044538	3044599	62	microRNA 4251
PRDM16	chr1	2985741	3355185	369445	PR domain containing 16
ARHGEF16	chr1	3371146	3397677	26532	Rho guanine nucleotide exchange factor (GEF) 16
MEGF6	chr1	3404505	3528059	123555	multiple EGF-like-domains 6
C1orf174	chr1	3805696	3816857	11162	chromosome 1 open reading frame 174
LINC01134	chr1	3816967	3832011	15045	long intergenic non-protein coding RNA 1134
LOC100133612	chr1	3816967	3833879	16913	long intergenic non-protein coding RNA 1134
LINC01346	chr1	4000671	4012643	11973	long intergenic non-protein coding RNA 1346
LOC728716	chr1	4000671	4012643	11973	long intergenic non-protein coding RNA 1346
LOC284661	chr1	4472110	4484744	12635	uncharacterized LOC284661
AJAP1	chr1	4715104	4843851	128748	adherens junctions associated protein 1
BC037321	chr1	4847557	4852183	4627	
MIR4417	chr1	5624130	5624203	74	microRNA 4417
AK125078	chr1	5621768	5728315	106548	
GPR153	chr1	6307405	6321035	13631	G protein-coupled receptor 153
ACOT7	chr1	6324331	6453826	129496	acyl-CoA thioesterase 7
HES2	chr1	6472497	6484730	12234	hes family bHLH transcription factor 2
MIR4252	chr1	6489893	6489956	64	microRNA 4252
ESPN	chr1	6484847	6521004	36158	espin
TNFRSF25	chr1	6521213	6526255	5043	tumor necrosis factor receptor superfamily, member 25
PLEKHG5	chr1	6526151	6580121	53971	pleckstrin homology domain containing, family G (with RhoGef domain) member 5
NOL9	chr1	6581406	6614658	33253	nucleolar protein 9
TAS1R1	chr1	6615337	6639817	24481	taste receptor, type 1, member 1
ZBTB48	chr1	6640050	6649340	9291	zinc finger and BTB domain containing 48
KLHL21	chr1	6650778	6662929	12152	kelch-like family member 21
CAMTA1	chr1	6845383	7829766	984384	calmodulin binding transcription activator 1
VAMP3	chr1	7831328	7841492	10165	vesicle-associated membrane protein 3
PER3	chr1	7844379	7905240	60862	period circadian clock 3
UTS2	chr1	7903142	7973294	70153	urotensin 2
TNFRSF9	chr1	7975930	8003225	27296	tumor necrosis factor receptor superfamily, member 9
TRNA_Pseudo	chr1	7990338	7990408	71	
SLC2A7	chr1	9063358	9086404	23047	intestinal facilitative glucose transporter 7
SLC2A5	chr1	9097004	9148510	51507	solute carrier family 2 (facilitated glucose/fructose transporter), member 5
SLC25A33	chr1	9599527	9642831	43305	solute carrier family 25 (pyrimidine nucleotide carrier), member 33
TMEM201	chr1	9648931	9674935	26005	transmembrane protein 201
C1orf200	chr1	9712667	9714644	1978	chromosome 1 open reading frame 200
PIK3CD	chr1	9711789	9789172	77384	phosphatidylinositol-4,5-bisphosphate 3-kinase, catalytic subunit delta
C1orf127	chr1	11006529	11042094	35566	chromosome 1 open reading frame 127
TARDBP	chr1	11072678	11085549	12872	TAR DNA binding protein
MASP2	chr1	11086579	11107296	20718	mannan-binding lectin serine peptidase 2
SRM	chr1	11114648	11120091	5444	spermidine synthase
EXOSC10	chr1	11126675	11159938	33264	exosome component 10
MTOR-AS1	chr1	11203954	11209595	5642	MTOR antisense RNA 1
MTOR	chr1	11166587	11322608	156022	mechanistic target of rapamycin (serine/threonine kinase)
ANGPTL7	chr1	11249345	11256038	6694	angiopoietin-like 7
UBIAD1	chr1	11333254	11348491	15238	UbiA prenyltransferase domain containing 1
LOC101929181	chr1	11669587	11673411	3825	uncharacterized LOC101929181
FBXO2	chr1	11708417	11714888	6472	F-box protein 2
FBXO44	chr1	11714431	11723384	8954	F-box protein 44
FBXO6	chr1	11724149	11734409	10261	F-box protein 6
AADACL4	chr1	12704565	12727097	22533	arylacetamide deacetylase-like 4
AADACL3	chr1	12776117	12788726	12610	arylacetamide deacetylase-like 3
C1orf158	chr1	12806133	12821102	14970	chromosome 1 open reading frame 158
PRAMEF12	chr1	12834990	12838048	3059	PRAME family member 12
PRAMEF1	chr1	12851545	12856777	5233	PRAME family member 1
PRAMEF11	chr1	12884617	12891264	6648	PRAME family member 11
LOC649330	chr1	12907229	12908235	1007	heterogeneous nuclear ribonucleoprotein C-like
HNRNPCL1	chr1	12907260	12908578	1319	heterogeneous nuclear ribonucleoprotein C-like 1
PRAMEF2	chr1	12916940	12921764	4825	PRAME family member 2
PRAMEF4	chr1	12939032	12946025	6994	PRAME family member 4
LOC101929983	chr1	12998504	13002353	3850	PRAME family member 9/15-like
PRAMEF9	chr1	12998504	13002353	3850	PRAME family member 9
LOC391003	chr1	13035498	13039011	3514	PRAME family member-like
PRAMEF5	chr1	12998301	13117751	119451	PRAME family member 5
LOC645354	chr1	13161985	13167193	5209	PRAME family member 1 pseudogene
LOC649324	chr1	13161985	13167193	5209	PRAME family member 1 pseudogene
HNRNPCL2	chr1	13182959	13184326	1368	heterogeneous nuclear ribonucleoprotein C-like 2
LOC440563	chr1	13182959	13184326	1368	heterogeneous nuclear ribonucleoprotein C-like 2
PRAMEF3	chr1	13328195	13331692	3498	
PRAMEF22	chr1	13328832	13331671	2840	PRAME family member 22
PRAMEF6	chr1	13359832	13368936	9105	PRAME family member 6

PRAMEF5	chr1	13359818	13369057	9240	PRAME family member 5
PRAMEF8	chr1	13386646	13390748	4103	PRAME family member 8
PRAMEF10	chr1	13409127	13414502	5376	PRAME family member 10
LOC645382	chr1	13409134	13414502	5369	PRAME family member 10-like
PRAMEF13	chr1	13447413	13452656	5244	PRAME family member 14
PRAMEF14	chr1	13447413	13452656	5244	PRAME family member 14
LOC400736	chr1	13447413	13452656	5244	PRAME family member-like
PRAMEF19	chr1	13474052	13477569	3518	PRAME family member 19
PRAMEF16	chr1	13495253	13498259	3007	PRAME family member 16
PRAMEF17	chr1	13495257	13498257	3001	PRAME family member 17
PRAMEF20	chr1	13516065	13526943	10879	PRAME family member 20
PRAMEF7	chr1	13607699	13611533	3835	PRAME family member 7
PRAMEF10	chr1	13629937	13635299	5363	PRAME family member 10
PRAMEF15	chr1	13641972	13648987	7016	PRAME family member 15
PRAMEF19	chr1	13694888	13698405	3518	PRAME family member 19
LRRC38	chr1	13801444	13840242	38799	leucine rich repeat containing 38
PRDM2	chr1	14026734	14151574	124841	PR domain containing 2, with ZNF domain
AK124197	chr1	14146461	14150513	4053	
KAZN	chr1	14925212	15444544	519333	kazrin, periplakin interacting protein
TMEM51-AS1	chr1	15438310	15478960	40651	TMEM51 antisense RNA 1
C1orf195	chr1	15490691	15498120	7430	chromosome 1 open reading frame 195
TMEM51	chr1	15479027	15546974	67948	transmembrane protein 51
FHAD1	chr1	15573767	15726778	153012	forkhead-associated (FHA) phosphopeptide binding domain 1
AK055853	chr1	15653175	15670372	17198	
EFHD2	chr1	15736390	15756839	20450	EF-hand domain family, member D2
CTRC	chr1	15764937	15773153	8217	chymotrypsin C (caldecrin)
CELA2A	chr1	15783222	15798586	15365	chymotrypsin-like elastase family, member 2A
CELA2B	chr1	15802595	15817895	15301	chymotrypsin-like elastase family, member 2B
CASP9	chr1	15817323	15851285	33963	caspase 9, apoptosis-related cysteine peptidase
PLEKHM2	chr1	16010826	16061264	50439	pleckstrin homology domain containing, family M (with RUN domain) member 2
SLC25A34	chr1	16062808	16067884	5077	solute carrier family 25, member 34
TMEM82	chr1	16068986	16074292	5307	transmembrane protein 82
FBLIM1	chr1	16083153	16113084	29932	filamin binding LIM protein 1
UQCRHL	chr1	16133656	16134194	539	ubiquinol-cytochrome c reductase hinge protein-like
FLJ37453	chr1	16160709	16174642	13934	uncharacterized LOC729614
SPEN	chr1	16174358	16266950	92593	spen family transcriptional repressor
ZBTB17	chr1	16268363	16302627	34265	zinc finger and BTB domain containing 17
DQ576383	chr1	16317618	16317647	30	
C1orf64	chr1	16330730	16333184	2455	chromosome 1 open reading frame 64
HSPB7	chr1	16340522	16346089	5568	heat shock 27kDa protein family, member 7 (cardiovascular)
CLCNKA	chr1	16345369	16360545	15177	chloride channel, voltage-sensitive Ka
CLCNKB	chr1	16355620	16383821	28202	chloride channel, voltage-sensitive Kb
FAM131C	chr1	16384263	16400127	15865	family with sequence similarity 131, member C
EPHA2	chr1	16450831	16482582	31752	EPH receptor A2
ARHGEF19	chr1	16524598	16539104	14507	Rho guanine nucleotide exchange factor (GEF) 19
RSG1	chr1	16558181	16563659	5479	REM2 and RAB-like small GTPase 1
FBXO42	chr1	16576558	16678948	102391	F-box protein 42
SZRD1	chr1	16693524	16724643	31120	SUZ RNA binding domain containing 1
AL832937	chr1	16721826	16724639	2814	
SPATA21	chr1	16722175	16763919	41745	spermatogenesis associated 21
NECAP2	chr1	16767166	16786584	19419	NECAP endocytosis associated 2
CROCCP3	chr1	16793930	16819196	25267	ciliary rootlet coiled-coil, rootletin pseudogene 3
TRNA_Asn	chr1	16847079	16847153	75	
BC036435	chr1	16860349	16862144	1796	
AX747988	chr1	16862254	16864669	2416	
TRNA_Gly	chr1	16872433	16872504	72	
MIR3675	chr1	16875408	16875482	75	microRNA 3675
NBPF1	chr1	16888921	16940100	51180	neuroblastoma breakpoint family, member 1
BC015342	chr1	16955701	16956958	1258	
CROCCP2	chr1	16944750	16971178	26429	ciliary rootlet coiled-coil, rootletin pseudogene 2
FLJ00313	chr1	16956164	16971178	15015	
MST1P2	chr1	16972068	16976915	4848	macrophage stimulating 1 (hepatocyte growth factor-like) pseudogene 2
FAM231A	chr1	16999497	17000007	511	family with sequence similarity 231, member A
TRNA_Val	chr1	17006500	17006573	74	
ESPNP	chr1	17017712	17046652	28941	espin pseudogene
TRNA_Pseudo	chr1	17052060	17052133	74	
TRNA_Gly	chr1	17053779	17053850	72	
MST1L	chr1	17081128	17090975	9848	macrophage stimulating 1-like
CROCC	chr1	17066767	17299474	232708	ciliary rootlet coiled-coil, rootletin
AK125737	chr1	17197439	17200574	3136	
TRNA_Asn	chr1	17201957	17202031	75	
BC070363	chr1	17215040	17216161	1122	
DL489931	chr1	17222645	17222720	76	
MFAP2	chr1	17300998	17308081	7084	microfibrillar-associated protein 2
ATP13A2	chr1	17312452	17338467	26016	ATPase type 13A2
PADI1	chr1	17531620	17572501	40882	peptidyl arginine deiminase, type I
Mir_584	chr1	17581660	17581778	119	
PADI3	chr1	17575592	17610727	35136	peptidyl arginine deiminase, type III
PADI4	chr1	17634689	17690495	55807	peptidyl arginine deiminase, type IV
PADI6	chr1	17698690	17728195	29506	peptidylarginine deiminase type 6

ARHGEF10L	chr1	17846826	18024370	177545	Rho guanine nucleotide exchange factor (GEF) 10-like
ARHGEF10L	chr1	17914910	17966476	51567	Rho guanine nucleotide exchange factor (GEF) 10-like
ACTL8	chr1	18081807	18153558	71752	actin-like 8
IGSF21	chr1	18434239	18704977	270739	immunoglobin superfamily, member 21
PAX7	chr1	18957499	19075360	117862	paired box 7
TAS1R2	chr1	19166092	19186155	20064	taste receptor, type 1, member 2
MIR4695	chr1	19209695	19209769	75	microRNA 4695
ALDH4A1	chr1	19197923	19229293	31371	aldehyde dehydrogenase 4 family, member A1
IFFO2	chr1	19230773	19282826	52054	intermediate filament family orphan 2
RPS14P3	chr1	19934300	19935138	839	ribosomal protein S14 pseudogene 3
MINOS1	chr1	19923470	19956315	32846	mitochondrial inner membrane organizing system 1
MINOS1-NBL1	chr1	19923470	19984949	61480	MINOS1-NBL1 readthrough
NBL1	chr1	19969722	19984949	15228	neuroblastoma 1, DAN family BMP antagonist
HTR6	chr1	19991779	20007459	15681	5-hydroxytryptamine (serotonin) receptor 6, G protein-coupled
TMCO4	chr1	20008705	20126758	118054	transmembrane and coiled-coil domains 4
RNF186	chr1	20140521	20141771	1251	ring finger protein 186
OTUD3	chr1	20208887	20239437	30551	OTU deubiquitinase 3
PLA2G2E	chr1	20246799	20250110	3312	phospholipase A2, group IIE
PLA2G2A	chr1	20301923	20306932	5010	phospholipase A2, group IIA (platelets, synovial fluid)
PLA2G5	chr1	20354671	20418394	63724	phospholipase A2, group V
PLA2G2D	chr1	20439142	20446059	6918	phospholipase A2, group IID
PLA2G2F	chr1	20465822	20476879	11058	phospholipase A2, group IIF
PLA2G2C	chr1	20490483	20501687	11205	phospholipase A2, group IIC
UBXN10-AS1	chr1	20510735	20512979	2245	UBXN10 antisense RNA 1
UBXN10	chr1	20512577	20519942	7366	UBX domain protein 10
VWA5B1	chr1	20617411	20681387	63977	von Willebrand factor A domain containing 5B1
LINC01141	chr1	20686293	20755287	68995	long intergenic non-protein coding RNA 1141
LOC339505	chr1	20686293	20755287	68995	long intergenic non-protein coding RNA 1141
CAMK2N1	chr1	20808883	20812728	3846	calcium/calmodulin-dependent protein kinase II inhibitor 1
MUL1	chr1	20825940	20834674	8735	mitochondrial E3 ubiquitin protein ligase 1
CDA	chr1	20915443	20945400	29958	cytidine deaminase
MIR6084	chr1	20960171	20960281	111	microRNA 6084
PINK1	chr1	20959947	20978004	18058	PTEN induced putative kinase 1
PINK1-AS	chr1	20969149	20978686	9538	PINK1 antisense RNA
DDOST	chr1	20978259	20988037	9779	dolichyl-diphosphooligosaccharide--protein glycosyltransferase subunit (non-catalytic)
KIF17	chr1	20990506	21044510	54005	kinesin family member 17
SH2D5	chr1	21046224	21059330	13107	SH2 domain containing 5
HP1BP3	chr1	21069170	21113799	44630	heterochromatin protein 1, binding protein 3
EIF4G3	chr1	21132784	21503381	370598	eukaryotic translation initiation factor 4 gamma, 3
AX747766	chr1	21602542	21604868	2327	
ECE1	chr1	21543739	21672034	128296	endothelin converting enzyme 1
LOC100506801	chr1	21619782	21626362	6581	uncharacterized LOC100506801
AK098438	chr1	21749600	21754300	4701	
BC127868	chr1	21761832	21762609	778	
NBPF3	chr1	21766582	21811393	44812	neuroblastoma breakpoint family, member 3
ALPL	chr1	21835857	21904905	69049	alkaline phosphatase, liver/bone/kidney
RAP1GAP	chr1	21922707	21995856	73150	RAP1 GTPase activating protein
USP48	chr1	22004791	22109688	104898	ubiquitin specific peptidase 48
LDLRAD2	chr1	22138757	22151714	12958	low density lipoprotein receptor class A domain containing 2
HSPG2	chr1	22148724	22263790	115067	heparan sulfate proteoglycan 2
CELA3A	chr1	22328148	22339035	10888	chymotrypsin-like elastase family, member 3A
LOC101928043	chr1	22350486	22352541	2056	uncharacterized LOC101928043
LINC00339	chr1	22351683	22357717	6035	long intergenic non-protein coding RNA 339
CDC42	chr1	22379119	22419436	40318	cell division cycle 42
WNT4	chr1	22443797	22470385	26589	wingless-type MMTV integration site family, member 4
AX747207	chr1	25227540	25230414	2875	
MIR6731	chr1	25245835	25245907	73	microRNA 6731
RUNX3	chr1	25226001	25291612	65612	runt-related transcription factor 3
C1orf63	chr1	25568739	25664656	95918	arginine/serine-rich protein 1
TMEM50A	chr1	25664788	25688852	24065	transmembrane protein 50A
RHCE	chr1	25688739	25756683	67945	Rhesus blood group C antigen
TMEM57	chr1	25757348	25826698	69351	transmembrane protein 57
LDLRAP1	chr1	25870075	25895377	25303	low density lipoprotein receptor adaptor protein 1
MAN1C1	chr1	25943958	26112698	168741	mannosidase, alpha, class 1C, member 1
SEPN1	chr1	26126666	26144713	18048	selenoprotein N, 1
LOC646471	chr1	26146444	26150097	3654	uncharacterized LOC646471
MTFR1L	chr1	26146396	26159433	13038	mitochondrial fission regulator 1-like
AUNIP	chr1	26158403	26185949	27547	aurora kinase A and ninein interacting protein
PAQR7	chr1	26187974	26197744	9771	progestin and adipoQ receptor family member VII
STMN1	chr1	26210676	26233368	22693	stathmin 1
MIR3917	chr1	26232852	26232945	94	microRNA 3917
BC042538	chr1	30486798	30510456	23659	
LOC101929406	chr1	30486798	30510459	23662	uncharacterized LOC101929406
SDC3	chr1	31342312	31381480	39169	syndecan 3
SNORD103A	chr1	31408535	31408623	89	small nucleolar RNA, C/D box 103A
SNORD103B	chr1	31408535	31408623	89	small nucleolar RNA, C/D box 103B
SNORD103A	chr1	31421964	31422052	89	small nucleolar RNA, C/D box 103A
SNORD85	chr1	31441009	31441084	76	small nucleolar RNA, C/D box 85
PUM1	chr1	31404352	31538763	134412	pumilio RNA-binding family member 1
NKAIN1	chr1	31652591	31712734	60144	Na+/K+ transporting ATPase interacting 1

SNRNP40	chr1	31732414	31769644	37231	small nuclear ribonucleoprotein 40kDa (U5)
ZCCHC17	chr1	31769828	31837800	67973	zinc finger, CCHC domain containing 17
FABP3	chr1	31838099	31845923	7825	fatty acid binding protein 3, muscle and heart (mammary-derived growth inhibitor)
SERINC2	chr1	31882411	31907527	25117	serine incorporator 2
LINC01225	chr1	31971838	31974167	2330	long intergenic non-protein coding RNA 1225
LOC149086	chr1	31971838	31974167	2330	long intergenic non-protein coding RNA 1225
LINC01226	chr1	31984035	31989846	5812	long intergenic non-protein coding RNA 1226
LOC284551	chr1	31984035	31989846	5812	long intergenic non-protein coding RNA 1226
TINAGL1	chr1	32042085	32053287	11203	tubulointerstitial nephritis antigen-like 1
HCRTR1	chr1	32083300	32098119	14820	hypocretin (orexin) receptor 1
AX747565	chr1	32095467	32098435	2969	
PEF1	chr1	32095462	32110838	15377	penta-EF-hand domain containing 1
COL16A1	chr1	32117847	32169768	51922	collagen, type XVI, alpha 1
BAI2	chr1	32192705	32229664	36960	brain-specific angiogenesis inhibitor 2
MIR4254	chr1	32224260	32224336	77	microRNA 4254
BC069257	chr1	32256024	32258424	2401	
SPOCD1	chr1	32256022	32281652	25631	SPOC domain containing 1
LCK	chr1	32716839	32751768	34930	LCK proto-oncogene, Src family tyrosine kinase
HDAC1	chr1	32757707	32799224	41518	histone deacetylase 1
MARCKSL1	chr1	32799429	32801840	2412	MARCKS-like 1
FAM229A	chr1	32826870	32827844	975	family with sequence similarity 229, member A
TSSK3	chr1	32827861	32829924	2064	testis-specific serine kinase 3
BSDC1	chr1	32830233	32860062	29830	BSD domain containing 1
ZBTB8B	chr1	32930657	32953459	22803	zinc finger and BTB domain containing 8B
ZBTB8A	chr1	32930657	33071551	140895	zinc finger and BTB domain containing 8A
ZBTB8OS	chr1	33065772	33116185	50414	zinc finger and BTB domain containing 8 opposite strand
S100PBP	chr1	33283042	33324480	41439	S100P binding protein
FNDC5	chr1	33327868	33338093	10226	fibronectin type III domain containing 5
HPCA	chr1	33352097	33360247	8151	hippocalcin
TMEM54	chr1	33360195	33366953	6759	transmembrane protein 54
RNF19B	chr1	33402046	33430414	28369	ring finger protein 19B
ZSCAN20	chr1	33938231	33961995	23765	zinc finger and SCAN domain containing 20
CSMD2	chr1	33979598	34631443	651846	CUB and Sushi multiple domains 2
HMGB4	chr1	34326075	34330392	4318	high mobility group box 4
CSMD2-AS1	chr1	34334556	34351059	16504	CSMD2 antisense RNA 1
LOC402779	chr1	34334556	34351059	16504	CSMD2 antisense RNA 1
C1orf94	chr1	34632483	34684731	52249	chromosome 1 open reading frame 94
GJB5	chr1	35220647	35224113	3467	gap junction protein, beta 5, 31.1kDa
GJB4	chr1	35225341	35229325	3985	gap junction protein, beta 4, 30.3kDa
Mir_548	chr1	35244163	35244247	85	
GJB3	chr1	35246789	35251967	5179	gap junction protein, beta 3, 31kDa
GJA4	chr1	35258170	35261348	3179	gap junction protein, alpha 4, 37kDa
SMIM12	chr1	35315962	35325417	9456	small integral membrane protein 12
DLGAP3	chr1	35331036	35370984	39949	discs, large (Drosophila) homolog-associated protein 3
LOC653160	chr1	35441299	35450948	9650	uncharacterized LOC653160
ZMYM6NB	chr1	35447126	35450948	3823	ZMYM6 neighbor
ZMYM6	chr1	35449367	35497569	48203	zinc finger, MYM-type 6
ZMYM1	chr1	35525386	35581459	56074	zinc finger, MYM-type 1
BX537811	chr1	35641980	35646083	4104	
AL831889	chr1	35641980	35646083	4104	
SFPQ	chr1	35649200	35658743	9544	splicing factor proline/glutamine-rich
ZMYM4	chr1	35734567	35887545	152979	zinc finger, MYM-type 4
AF119915	chr1	35833677	35835012	1336	
KIAA0319L	chr1	35899090	36023551	124462	KIAA0319-like
NCDN	chr1	36023392	36032380	8989	neurochondrin
TFAP2E	chr1	36038970	36060927	21958	transcription factor AP-2 epsilon (activating enhancer binding protein 2 epsilon)
PSMB2	chr1	36035412	36107445	72034	proteasome (prosome, macropain) subunit, beta type, 2
C1orf216	chr1	36179476	36184790	5315	chromosome 1 open reading frame 216
CLSPN	chr1	36197712	36235551	37840	claspin
AGO4	chr1	36273827	36323490	49664	argonaute RISC catalytic component 4
AGO1	chr1	36335408	36389899	54492	argonaute RISC catalytic component 1
AK025726	chr1	36391432	36395210	3779	
AGO3	chr1	36396318	36522063	125746	argonaute RISC catalytic component 3
TEKT2	chr1	36549675	36553876	4202	tektin 2 (testicular)
CSF3R	chr1	36931643	36948915	17273	colony stimulating factor 3 receptor (granulocyte)
GRIK3	chr1	37261127	37499844	238718	glutamate receptor, ionotropic, kainate 3
MIR4255	chr1	37627163	37627235	73	microRNA 4255
AX747480	chr1	38147242	38149864	2623	
C1orf109	chr1	38147241	38156192	8952	chromosome 1 open reading frame 109
CDCA8	chr1	38158072	38175391	17320	cell division cycle associated 8
EPHA10	chr1	38179552	38230824	51273	EPH receptor A10
INPP5B	chr1	38326368	38412729	86362	inositol polyphosphate-5-phosphatase, 75kDa
SF3A3	chr1	38422651	38455761	33111	splicing factor 3a, subunit 3, 60kDa
FHL3	chr1	38462441	38471187	8747	four and a half LIM domains 3
UTP11L	chr1	38478383	38490497	12115	UTP11-like, U3 small nucleolar ribonucleoprotein (yeast)
POU3F1	chr1	38509522	38512450	2929	POU class 3 homeobox 1
MIR3659	chr1	38554902	38555001	100	microRNA 3659
LINC01343	chr1	38674705	38680439	5735	long intergenic non-protein coding RNA 1343
LOC339442	chr1	38674705	38680439	5735	long intergenic non-protein coding RNA 1343
RRAGC	chr1	39303868	39340166	36299	Ras-related GTP binding C

MYCBP	chr1	39328161	39339050	10890	MYC binding protein
GJA9-MYCBP	chr1	39328161	39347298	19138	GJA9-MYCBP readthrough
GJA9	chr1	39339738	39347298	7561	gap junction protein, alpha 9, 59kDa
RHBDL2	chr1	39351478	39407456	55979	rhomboid, veinlet-like 2 (Drosophila)
MACF1	chr1	39670422	39748740	78319	microtubule-actin crosslinking factor 1
MACF1	chr1	39547088	39952810	405723	microtubule-actin crosslinking factor 1
KIAA0754	chr1	39875175	39882154	6980	KIAA0754
BMP8A	chr1	39957317	39995541	38225	bone morphogenetic protein 8a
PPIEL	chr1	39987951	40025370	37420	peptidylprolyl isomerase E-like pseudogene
SNORA55	chr1	40033045	40033182	138	small nucleolar RNA, H/ACA box 55
PABPC4	chr1	40026484	40042521	16038	poly(A) binding protein, cytoplasmic 4 (inducible form)
LOC10192951€	chr1	40030741	40038875	8135	uncharacterized LOC101929516
HEYL	chr1	40089102	40105348	16247	hes-related family bHLH transcription factor with YRPW motif-like
NT5C1A	chr1	40124792	40137710	12919	5'-nucleotidase, cytosolic IA
HPCAL4	chr1	40144319	40157382	13064	hippocalcin like 4
PPIE	chr1	40204516	40229586	25071	peptidylprolyl isomerase E (cyclophilin E)
AB075489	chr1	40222853	40225715	2863	
OXCT2	chr1	40235196	40237020	1825	3-oxoacid CoA transferase 2
BMP8B	chr1	40223902	40254533	30632	bone morphogenetic protein 8b
TRIT1	chr1	40306705	40349177	42473	tRNA isopentenyltransferase 1
MYCL	chr1	40361095	40367687	6593	v-myc avian myelocytomatosis viral oncogene lung carcinoma derived homolog
MYCL1	chr1	40361095	40367687	6593	
BC069694	chr1	40420819	40422587	1769	
MFSD2A	chr1	40420783	40435640	14858	major facilitator superfamily domain containing 2A
CAP1	chr1	40506254	40538321	32068	CAP, adenylate cyclase-associated protein 1 (yeast)
PPT1	chr1	40538381	40563142	24762	palmitoyl-protein thioesterase 1
RLF	chr1	40627040	40706593	79554	rearranged L-myc fusion
TMCO2	chr1	40713572	40717365	3794	transmembrane and coiled-coil domains 2
ZMPSTE24	chr1	40723721	40759856	36136	zinc metallopeptidase STE24
COL9A2	chr1	40766162	40783060	16899	collagen, type IX, alpha 2
ZFP69	chr1	40943301	40962015	18715	ZFP69 zinc finger protein
EXO5	chr1	40974432	40982214	7783	exonuclease 5
ZNF684	chr1	40997232	41013841	16610	zinc finger protein 684
RIMS3	chr1	41086351	41131324	44974	regulating synaptic membrane exocytosis 3
EFCAB14-AS1	chr1	47139707	47157769	18063	EFCAB14 antisense RNA 1
EFCAB14	chr1	47124358	47184736	60379	EF-hand calcium binding domain 14
CYP4B1	chr1	47264669	47285021	20353	cytochrome P450, family 4, subfamily B, polypeptide 1
CYP4Z2P	chr1	47308766	47366147	57382	cytochrome P450, family 4, subfamily Z, polypeptide 2, pseudogene
CYP4A11	chr1	47394845	47407156	12312	cytochrome P450, family 4, subfamily A, polypeptide 11
CYP4X1	chr1	47427035	47516423	89389	cytochrome P450, family 4, subfamily X, polypeptide 1
CYP4Z1	chr1	47533159	47583992	50834	cytochrome P450, family 4, subfamily Z, polypeptide 1
CYP4A22	chr1	47603106	47614526	11421	cytochrome P450, family 4, subfamily A, polypeptide 22
LINC00853	chr1	47644921	47646011	1091	long intergenic non-protein coding RNA 853
PDZK1IP1	chr1	47649260	47655771	6512	PDZK1 interacting protein 1
TAL1	chr1	47681961	47698007	16047	T-cell acute lymphocytic leukemia 1
JA375062	chr1	47691628	47691655	28	
STIL	chr1	47694867	47779819	84953	SCL/TAL1 interrupting locus
CMPK1	chr1	47799468	47844511	45044	cytidine monophosphate (UMP-CMP) kinase 1, cytosolic
AX748181	chr1	47859449	47861215	1767	
TRABD2B	chr1	48226199	48462562	236364	TraB domain containing 2B
SKINTL	chr1	48567386	48648100	80715	Skint-like, pseudogene
SLC5A9	chr1	48688356	48714316	25961	solute carrier family 5 (sodium/sugar cotransporter), member 9
SPATA6	chr1	48761043	48937880	176838	spermatogenesis associated 6
BEND5	chr1	49193539	49242547	49009	BEN domain containing 5
LOC101929721	chr1	49723082	49734966	11885	uncharacterized LOC101929721
AGBL4	chr1	48998526	50489626	1491101	ATP/GTP binding protein-like 4
AGBL4-IT1	chr1	49839872	49937757	97886	AGBL4 intronic transcript 1 (non-protein coding)
ELAVL4	chr1	50513685	50669457	155773	ELAV like neuron-specific RNA binding protein 4
DMRTA2	chr1	50883222	50889119	5898	DMRT-like family A2
FAF1	chr1	50906934	51425936	519003	Fas (TNFRSF6) associated factor 1
CDKN2C	chr1	51434366	51440309	5944	cyclin-dependent kinase inhibitor 2C (p18, inhibits CDK4)
C1orf185	chr1	51567905	51613754	45850	chromosome 1 open reading frame 185
RNF11	chr1	51701944	51739119	37176	ring finger protein 11
TTC39A	chr1	51752929	51810788	57860	tetratricopeptide repeat domain 39A
TTC39A-AS1	chr1	51795325	51796953	1629	TTC39A antisense RNA 1
EPS15	chr1	51819934	51984995	165062	epidermal growth factor receptor pathway substrate 15
OSBPL9	chr1	52042850	52254891	212042	oxysterol-binding protein-like protein 9
RAB3B	chr1	52373627	52456436	82810	RAB3B, member RAS oncogene family
KTI12	chr1	52497776	52499472	1697	KTI12 homolog, chromatin associated (S. cerevisiae)
TXNDC12	chr1	52485803	52521843	36041	thioredoxin domain containing 12 (endoplasmic reticulum)
BTF3L4	chr1	52521856	52556388	34533	basic transcription factor 3-like 4
ZFYVE9	chr1	52607765	52812358	204594	zinc finger, FYVE domain containing 9
CC2D1B	chr1	52816264	52831877	15614	coiled-coil and C2 domain containing 1B
ORC1	chr1	52838500	52870143	31644	origin recognition complex, subunit 1
PRPF38A	chr1	52870218	52883992	13775	pre-mRNA processing factor 38A
ZCCHC11	chr1	52888947	53019130	130184	zinc finger, CCHC domain containing 11
GPX7	chr1	53068042	53074723	6682	glutathione peroxidase 7
FAM159A	chr1	53099065	53135339	36275	family with sequence similarity 159, member A
SELRC1	chr1	53152013	53164038	12026	
COA7	chr1	53152013	53164038	12026	cytochrome c oxidase assembly factor 7 (putative)

ZYG11B	chr1	53192130	53293013	100884	zyg-11 family member B, cell cycle regulator
ZYG11A	chr1	53308182	53360247	52066	zyg-11 family member A, cell cycle regulator
ECHDC2	chr1	53361581	53392851	31271	enoyl CoA hydratase domain containing 2
SCP2	chr1	53392900	53517289	124390	sterol carrier protein 2
PODN	chr1	53527723	53551174	23452	podocan
SLC1A7	chr1	53552850	53608304	55455	solute carrier family 1 (glutamate transporter), member 7
AX748428	chr1	53580247	53584281	4035	
CPT2	chr1	53662100	53679869	17770	carnitine palmitoyltransferase 2
C1orf123	chr1	53679771	53686289	6519	chromosome 1 open reading frame 123
MAGOH	chr1	53692563	53704282	11720	mago-nashi homolog, proliferation-associated (Drosophila)
LOC100507564	chr1	53704281	53708455	4175	uncharacterized LOC100507564
LRP8	chr1	53708040	53793821	85782	low density lipoprotein receptor-related protein 8, apolipoprotein e receptor
AK097571	chr1	53793904	53802889	8986	
SLC25A3P1	chr1	53904042	53905693	1652	solute carrier family 25 (mitochondrial carrier; phosphate carrier), member 3 pseudogene 1
DMRTB1	chr1	53925071	53933158	8088	DMRT-like family B with proline-rich C-terminal, 1
GLIS1	chr1	53971905	54199877	227973	GLIS family zinc finger 1
NDC1	chr1	54231133	54304225	73093	NDC1 transmembrane nucleoporin
TMEM59	chr1	54497348	54519111	21764	transmembrane protein 59
MIR4781	chr1	54519751	54519827	77	microRNA 4781
TCEANC2	chr1	54519273	54578192	58920	transcription elongation factor A (SII) N-terminal and central domain containing 2
CDCP2	chr1	54604667	54618679	14013	CUB domain containing protein 2
ACOT11	chr1	55007929	55100417	92489	acyl-CoA thioesterase 11
MROH7	chr1	55107412	55175940	68529	maestro heat-like repeat family member 7
MROH7-TTC4	chr1	55107412	55208328	100917	MROH7-TTC4 readthrough (NMD candidate)
HEATR8-TTC4	chr1	55107426	55208328	100903	MROH7-TTC4 readthrough (NMD candidate)
TTC4	chr1	55181494	55208328	26835	tetratricopeptide repeat domain 4
PARS2	chr1	55222570	55230226	7657	prolyl-tRNA synthetase 2, mitochondrial (putative)
TTC22	chr1	55246751	55266941	20191	tetratricopeptide repeat domain 22
C1orf177	chr1	55271735	55307937	36203	chromosome 1 open reading frame 177
TRNA_Lys	chr1	55423541	55423614	74	
TMEM61	chr1	55446464	55457966	11503	transmembrane protein 61
BSND	chr1	55464616	55474465	9850	barttin CLCNK-type chloride channel accessory beta subunit
PCSK9	chr1	55505148	55530526	25379	proprotein convertase subtilisin/kexin type 9
USP24	chr1	55532031	55681039	149009	ubiquitin specific peptidase 24
LOC100507634	chr1	55681080	55683128	2049	uncharacterized LOC100507634
MIR4422	chr1	55691313	55691396	84	microRNA 4422
Y_RNA	chr1	55950543	55950645	103	
AK127270	chr1	56046709	56200675	153967	
PPAP2B	chr1	56960418	57045257	84840	phosphatidic acid phosphatase type 2B
PRKAA2	chr1	57110989	57181008	70020	protein kinase, AMP-activated, alpha 2 catalytic subunit
C1orf168	chr1	57184476	57285369	100894	chromosome 1 open reading frame 168
BC048114	chr1	57289353	57292593	3241	
C8A	chr1	57320442	57383894	63453	complement component 8, alpha polypeptide
C8B	chr1	57394882	57431813	36932	complement component 8, beta polypeptide
DAB1	chr1	57463578	59012446	1548869	Dab, reelin signal transducer, homolog 1 (Drosophila)
BC047487	chr1	58326214	58328786	2573	
DAB1-AS1	chr1	58326214	58328786	2573	DAB1 antisense RNA 1
BC033978	chr1	58326265	58328786	2522	
AX746780	chr1	58933598	58934677	1080	
OMA1	chr1	58939503	59012446	72944	OMA1 zinc metallopeptidase
TACSTD2	chr1	59041094	59043166	2073	tumor-associated calcium signal transducer 2
MYSM1	chr1	59120410	59165747	45338	Myb-like, SWIRM and MPN domains 1
JUN	chr1	59246462	59249785	3324	jun proto-oncogene
LINC01135	chr1	59250822	59365384	114563	long intergenic non-protein coding RNA 1135
LOC10013106C	chr1	59250822	59365384	114563	long intergenic non-protein coding RNA 1135
LINC01358	chr1	59486147	59553919	67773	long intergenic non-protein coding RNA 1358
HSD52	chr1	59597607	59612479	14873	uncharacterized LOC729467
FGGY	chr1	59762624	60228402	465779	FGGY carbohydrate kinase domain containing
MIR4711	chr1	60198898	60198968	71	microRNA 4711
LOC101926944	chr1	60238466	60254501	16036	uncharacterized LOC101926944
BC042048	chr1	60238466	60254501	16036	
HOOK1	chr1	60280462	60342050	61589	hook microtubule-tethering protein 1
CYP2J2	chr1	60358979	60392423	33445	cytochrome P450, family 2, subfamily J, polypeptide 2
C1orf87	chr1	60454823	60539442	84620	chromosome 1 open reading frame 87
LOC101926964	chr1	61125302	61291256	165955	uncharacterized LOC101926964
AK097193	chr1	61125302	61291256	165955	
NFIA-AS2	chr1	61405915	61436448	30534	NFIA antisense RNA 2
BC030753	chr1	61405915	61436448	30534	
NFIA-AS1	chr1	61714616	61719190	4575	NFIA antisense RNA 1
NFIA	chr1	61542945	61928460	385516	nuclear factor I/A
MGC34796	chr1	62119913	62121800	1888	sepiapterin reductase (7,8-dihydrobiopterin:NADP+ oxidoreductase) pseudogene
TM2D1	chr1	62146718	62191095	44378	TM2 domain containing 1
INADL	chr1	62208148	62644347	436200	InaD-like (Drosophila)
L1TD1	chr1	62660473	62678001	17529	LINE-1 type transposase domain containing 1
KANK4	chr1	62701836	62785083	83248	KN motif and ankyrin repeat domains 4
USP1	chr1	62901974	62917475	15502	ubiquitin specific peptidase 1
DOCK7	chr1	62920396	63154039	233644	dedicator of cytokinesis 7
ANGPTL3	chr1	63063157	63071976	8820	angiopoietin-like 3
ATG4C	chr1	63249776	63330941	81166	autophagy related 4C, cysteine peptidase
LINC00466	chr1	63624753	63782901	158149	long intergenic non-protein coding RNA 466

7SK	chr1	63704613	63704845	233	RNA, 7SK small nuclear
FOXD3-AS1	chr1	63786554	63790112	3559	FOXD3 antisense RNA 1 (head to head)
FOXD3	chr1	63788729	63790797	2069	forkhead box D3
MIR6068	chr1	63792595	63792655	61	microRNA 6068
U7	chr1	63799429	63799491	63	RNA, U7 small nuclear 86 pseudogene
ALG6	chr1	63833260	63904233	70974	ALG6, alpha-1,3-glucosyltransferase
ITGB3BP	chr1	63906440	63988944	82505	integrin beta 3 binding protein (beta3-endonexin)
EFCAB7	chr1	63989012	64038364	49353	EF-hand calcium binding domain 7
DLEU2L	chr1	64014650	64016307	1658	deleted in lymphocytic leukemia 2-like
PGM1	chr1	64058946	64125916	66971	phosphoglucomutase 1
Mir_544	chr1	64262024	64262128	105	
ROR1	chr1	64239689	64647179	407491	receptor tyrosine kinase-like orphan receptor 1
BC040909	chr1	64560124	64577888	17765	
ROR1-AS1	chr1	64560124	64579474	19351	ROR1 antisense RNA 1
AK096291	chr1	64571005	64636980	65976	
UBE2U	chr1	64669489	64710027	40539	ubiquitin-conjugating enzyme E2U (putative)
MIR4794	chr1	65045529	65045604	76	microRNA 4794
CACHD1	chr1	64936475	65158741	222267	cache domain containing 1
AK026777	chr1	65154981	65158736	3756	
RAVER2	chr1	65210777	65298914	88138	ribonucleoprotein, PTB-binding 2
AK128734	chr1	65338382	65343856	5475	
JAK1	chr1	65298905	65432619	133715	Janus kinase 1
BC035370	chr1	65445259	65468159	22901	
LINC01359	chr1	65445259	65468159	22901	long intergenic non-protein coding RNA 1359
MIR3671	chr1	65523437	65523525	89	microRNA 3671
MIR101-1	chr1	65524116	65524191	76	microRNA 101-1
AK4	chr1	65613231	65697828	84598	adenylate kinase 4
AK123450	chr1	65720144	65721848	1705	
DNAJC6	chr1	65720147	65881552	161406	DnaJ (Hsp40) homolog, subfamily C, member 6
LEPROT	chr1	65886130	65901690	15561	leptin receptor overlapping transcript
LEPR	chr1	65886130	66103176	217047	leptin receptor
LOC101927139	chr1	66508182	66516401	8220	uncharacterized LOC101927139
PDE4B	chr1	66258192	66840262	582071	phosphodiesterase 4B, cAMP-specific
U4	chr1	66560143	66560229	87	RNA, U4A small nuclear
MIR3117	chr1	67094122	67094200	79	microRNA 3117
SGIP1	chr1	66999824	67210768	210945	SH3-domain GRB2-like (endophilin) interacting protein 1
AK298300	chr1	67132271	67142710	10440	
TCTEX1D1	chr1	67218139	67244730	26592	Tctex1 domain containing 1
INSL5	chr1	67263423	67266942	3520	insulin-like 5
WDR78	chr1	67278571	67390570	112000	WD repeat domain 78
MIER1	chr1	67390577	67454302	63726	mesoderm induction early response 1, transcriptional regulator
SLC35D1	chr1	67465014	67520080	55067	solute carrier family 35 (UDP-GlcA/UDP-GalNAc transporter), member D1
C1orf141	chr1	67557858	67600654	42797	chromosome 1 open reading frame 141
IL23R	chr1	67604589	67725650	121062	interleukin 23 receptor
IL12RB2	chr1	67773046	67862583	89538	interleukin 12 receptor, beta 2
SERBP1	chr1	67873492	67896123	22632	SERPINE1 mRNA binding protein 1
GADD45A	chr1	68150859	68154021	3163	growth arrest and DNA-damage-inducible, alpha
GNG12	chr1	68167148	68299155	132008	guanine nucleotide binding protein (G protein), gamma 12
U7	chr1	68238275	68238336	62	RNA, U7 small nuclear 86 pseudogene
GNG12-AS1	chr1	68297970	68668670	370701	GNG12 antisense RNA 1
DIRAS3	chr1	68511644	68516460	4817	DIRAS family, GTP-binding RAS-like 3
WLS	chr1	68564141	68698284	134144	wntless Wnt ligand secretion mediator
MIR1262	chr1	68649200	68649293	94	microRNA 1262
JB175146	chr1	68649301	68649321	21	
RPE65	chr1	68894506	68915642	21137	retinal pigment epithelium-specific protein 65kDa
DEPDC1	chr1	68939834	68962904	23071	DEP domain containing 1
DEPDC1-AS1	chr1	68962358	69004310	41953	DEPDC1 antisense RNA 1
BC020917	chr1	68962358	69004310	41953	
LRRC7	chr1	70032867	70589171	556305	leucine rich repeat containing 7
PIN1P1	chr1	70385004	70386000	997	peptidylprolyl cis/trans isomerase, NIMA-interacting 1 pseudogene 1
LRRC40	chr1	70610484	70671361	60878	leucine rich repeat containing 40
SRSF11	chr1	70671364	70717701	46338	serine/arginine-rich splicing factor 11
ANKRD13C	chr1	70724684	70820417	95734	ankyrin repeat domain 13C
HHLA3	chr1	70820492	70833705	13214	HERV-H LTR-associating 3
CTH	chr1	70876900	70905534	28635	cystathionine gamma-lyase
LOC101927244	chr1	71172135	71252151	80017	uncharacterized LOC101927244
BC041441	chr1	71172135	71252151	80017	
PTGER3	chr1	71318035	71513491	195457	prostaglandin E receptor 3 (subtype EP3)
ZRANB2-AS2	chr1	71547006	71703406	156401	ZRANB2 antisense RNA 2 (head to head)
NEGR1	chr1	71868624	72748405	879782	neuronal growth regulator 1
SLC44A5	chr1	75667815	76143610	475796	solute carrier family 44, member 5
CR936677	chr1	76103850	76188721	84872	
ACADM	chr1	76190031	76229363	39333	acyl-CoA dehydrogenase, C-4 to C-12 straight chain
SNORD45C	chr1	76252756	76252834	79	small nucleolar RNA, C/D box 45C
SNORD45A	chr1	76253573	76253657	85	small nucleolar RNA, C/D box 45A
SNORD45B	chr1	76255161	76255232	72	small nucleolar RNA, C/D box 45B
RABGGTB	chr1	76251878	76260775	8898	Rab geranylgeranyltransferase, beta subunit
MSH4	chr1	76262555	76378923	116369	mutS homolog 4
MGC27382	chr1	78695282	78835147	139866	uncharacterized MGC27382
PTGFR	chr1	78956727	79006386	49660	prostaglandin F receptor (FP)

IFI44L	chr1	79086087	79111830	25744	interferon-induced protein 44-like
IFI44	chr1	79115476	79129763	14288	interferon-induced protein 44
Mir_548	chr1	79152744	79152828	85	
ELTD1	chr1	79355448	79472495	117048	EGF, latrophilin and seven transmembrane domain containing 1
LOC101927412	chr1	81001439	81112473	111035	uncharacterized LOC101927412
LOC101927434	chr1	81979564	82023387	43824	uncharacterized LOC101927434
LPHN2	chr1	81771844	82458422	686579	latrophilin 2
Mir_544	chr1	82312940	82313045	106	
U80773	chr1	83439565	83451891	12327	
LINC01361	chr1	83439565	83451891	12327	long intergenic non-protein coding RNA 1361
BC043544	chr1	83911736	83920454	8719	
LOC101927587	chr1	84041470	84326679	285210	uncharacterized LOC101927587
BC036594	chr1	84041470	84326679	285210	
HH834010	chr1	84259583	84259634	52	
LOC10192756C	chr1	84267198	84326229	59032	uncharacterized LOC101927560
AK097722	chr1	84267442	84326229	58788	
MIR548AP	chr1	84259597	84379059	119463	microRNA 548ap
TTLL7	chr1	84335056	84464833	129778	tubulin tyrosine ligase-like family, member 7
BC037304	chr1	84543635	84546350	2716	
PRKACB	chr1	84543657	84704181	160525	protein kinase, cAMP-dependent, catalytic, beta
SNORA2	chr1	84743003	84743140	138	
SAMD13	chr1	84764048	84816481	52434	sterile alpha motif domain containing 13
UOX	chr1	84830640	84863576	32937	urate oxidase, pseudogene
DNASE2B	chr1	84864214	84880691	16478	deoxyribonuclease II beta
RPF1	chr1	84944919	84964033	19115	ribosome production factor 1 homolog (S. cerevisiae)
GNG5	chr1	84964005	84972262	8258	guanine nucleotide binding protein (G protein), gamma 5
SPATA1	chr1	84972012	85018767	46756	spermatogenesis associated 1
CTBS	chr1	85018803	85040163	21361	chitobiase, di-N-acetyl-
LINC01461	chr1	85063646	85086714	23069	long intergenic non-protein coding RNA 1461
C1orf180	chr1	85093912	85100703	6792	chromosome 1 open reading frame 180
SSX2IP	chr1	85109389	85156440	47052	synovial sarcoma, X breakpoint 2 interacting protein
LPAR3	chr1	85279085	85358896	79812	lysophosphatidic acid receptor 3
MCOLN2	chr1	85391265	85462796	71532	mucolipin 2
MCOLN3	chr1	85490240	85495642	5403	mucolipin 3
MCOLN3	chr1	85483764	85514223	30460	mucolipin 3
WDR63	chr1	85527980	85598821	70842	WD repeat domain 63
MIR4423	chr1	85599476	85599556	81	microRNA 4423
SYDE2	chr1	85623355	85666728	43374	synapse defective 1, Rho GTPase, homolog 2 (C. elegans)
C1orf52	chr1	85715636	85725355	9720	chromosome 1 open reading frame 52
BCL10	chr1	85731459	85742587	11129	B-cell CLL/lymphoma 10
LOC646626	chr1	85742040	85865646	123607	uncharacterized LOC646626
DDAH1	chr1	85784167	86044046	259880	dimethylarginine dimethylaminohydrolase 1
CYR61	chr1	86046443	86049648	3206	cysteine-rich, angiogenic inducer, 61
AL832891	chr1	86047526	86049388	1863	
ZNHIT6	chr1	86115105	86174116	59012	zinc finger, HIT-type containing 6
COL24A1	chr1	86194915	86622446	427532	collagen, type XXIV, alpha 1
DR1	chr1	93811477	93828148	16672	down-regulator of transcription 1, TBP-binding (negative cofactor 2)
FNBP1L	chr1	93913687	94020218	106532	formin binding protein 1-like
TRNA_Cys	chr1	93981833	93981906	74	
LOC10012904€	chr1	94057524	94065587	8064	uncharacterized LOC100129046
BCAR3	chr1	94027342	94312706	285365	breast cancer anti-estrogen resistance 3
MIG7	chr1	94219111	94240930	21820	mig-7
MIR760	chr1	94312387	94312467	81	microRNA 760
TRNA_Arg	chr1	94313128	94313213	86	
AX746627	chr1	94317792	94319887	2096	
DNTTIP2	chr1	94335013	94344762	9750	deoxynucleotidyltransferase, terminal, interacting protein 2
GCLM	chr1	94352589	94375012	22424	glutamate-cysteine ligase, modifier subunit
ABCA4	chr1	94458393	94586705	128313	ATP-binding cassette, sub-family A (ABC1), member 4
ARHGAP29	chr1	94615402	94740624	125223	Rho GTPase activating protein 29
ABCD3	chr1	94883932	94984219	100288	ATP-binding cassette, sub-family D (ALD), member 3
F3	chr1	94994731	95007413	12683	coagulation factor III (thromboplastin, tissue factor)
BC030750	chr1	95123088	95285834	162747	
LINC01057	chr1	95123088	95285837	162750	long intergenic non-protein coding RNA 1057
SLC44A3	chr1	95285897	95360803	74907	solute carrier family 44, member 3
CNN3	chr1	95362504	95392779	30276	calponin 3, acidic
LOC729970	chr1	95393583	95428826	35244	hCG2028352-like
ALG14	chr1	95448278	95538507	90230	ALG14, UDP-N-acetylglucosaminyltransferase subunit
Mir_448	chr1	95550007	95550119	113	
TMEM56	chr1	95558072	95663161	105090	transmembrane protein 56
TMEM56-RWD	chr1	95583478	95710509	127032	TMEM56-RWDD3 readthrough
LOC101928118	chr1	95628774	95699538	70765	uncharacterized LOC101928118
AK090700	chr1	95628774	95699538	70765	
RWDD3	chr1	95699710	95712781	13072	RWD domain containing 3
FLJ31662	chr1	95940292	95944912	4621	uncharacterized LOC440594
LOC100996635	chr1	95975671	95981020	5350	uncharacterized LOC100996635
BC067883	chr1	95975895	95981020	5126	
LOC102723661	chr1	96457623	96488436	30814	uncharacterized LOC102723661
LOC101928241	chr1	96719624	96839681	120058	uncharacterized LOC101928241
PTBP2	chr1	97187160	97280605	93446	polypyrimidine tract binding protein 2
PTBLP	chr1	97279223	97280347	1125	polypyrimidine tract binding protein 2

DPYD-AS1	chr1	97561478	97788511	227034	DPYD antisense RNA 1
DPYD	chr1	97543299	98386615	843317	dihydropyrimidine dehydrogenase
DPYD-AS2	chr1	98262476	98263607	1132	DPYD antisense RNA 2
MIR137HG	chr1	98453555	98515249	61695	MIR137 host gene (non-protein coding)
MIR2682	chr1	98510798	98510907	110	microRNA 2682
MIR137	chr1	98511625	98511727	103	microRNA 137
LOC729987	chr1	98676266	98738214	61949	uncharacterized LOC729987
SNX7	chr1	99127235	99226056	98822	sorting nexin 7
LPPR5	chr1	99355800	99470449	114650	lipid phosphate phosphatase-related protein type 5
LOC10012962(	chr1	99469831	99614408	144578	uncharacterized LOC100129620
LPPR4	chr1	99729847	99775138	45292	lipid phosphate phosphatase-related protein type 4
LOC10192827(	chr1	99937975	99999571	61597	uncharacterized LOC101928270
PALMD	chr1	100111430	100160097	48668	palmdelphin
MIR548AA1	chr1	100154610	100178513	23904	microRNA 548aa-1
MIR548D1	chr1	100154610	100178513	23904	microRNA 548d-1
FRRS1	chr1	100174258	100231349	57092	ferric-chelate reductase 1
AGL	chr1	100315639	100389579	73941	amylo-alpha-1, 6-glucosidase, 4-alpha-glucanotransferase
BC112312	chr1	100434000	100435404	1405	
SLC35A3	chr1	100435344	100492534	57191	solute carrier family 35 (UDP-N-acetylglucosamine (UDP-GlcNAc) transporter), member A3
HIAT1	chr1	100503788	100548929	45142	hippocampus abundant transcript 1
SASS6	chr1	100549101	100598511	49411	spindle assembly 6 homolog (C. elegans)
TRMT13	chr1	100598705	100616054	17350	tRNA methyltransferase 13 homolog (S. cerevisiae)
LRRC39	chr1	100614003	100643829	29827	leucine rich repeat containing 39
DBT	chr1	100652477	100715409	62933	dihydrolipoamide branched chain transacylase E2
RTCA-AS1	chr1	100730297	100731730	1434	RTCA antisense RNA 1
RTCA	chr1	100731713	100758325	26613	RNA 3'-terminal phosphate cyclase
MIR553	chr1	100746796	100746864	69	microRNA 553
CDC14A	chr1	100810583	100985833	175251	cell division cycle 14A
GPR88	chr1	101003727	101007583	3857	G protein-coupled receptor 88
LINC01349	chr1	101092605	101112560	19956	long intergenic non-protein coding RNA 1349
LOC100128787	chr1	101092605	101112560	19956	long intergenic non-protein coding RNA 1349
VCAM1	chr1	101185195	101204601	19407	vascular cell adhesion molecule 1
EXTL2	chr1	101337927	101360735	22809	exostosin-like glycosyltransferase 2
SLC30A7	chr1	101361631	101447311	85681	solute carrier family 30 (zinc transporter), member 7
DPH5	chr1	101455179	101491644	36466	diphthamide biosynthesis 5
BX538249	chr1	101491408	101552819	61412	
LOC102606465	chr1	101491401	101552935	61535	uncharterized LOC102606465
BC045807	chr1	101538215	101552819	14605	
AK021551	chr1	101548009	101552819	4811	
LOC10192837(	chr1	101700428	101702084	1657	uncharacterized LOC101928370
S1PR1	chr1	101702304	101707076	4773	sphingosine-1-phosphate receptor 1
RNU6-31P	chr1	101806424	101806451	28	RNA, U6 small nuclear 31, pseudogene
DNAJA1P5	chr1	102337566	102360299	22734	DnaJ (Hsp40) homolog, subfamily A, member 1 pseudogene 5
OLFM3	chr1	102268122	102462790	194669	olfactomedin 3
COL11A1	chr1	103342022	103574052	232031	collagen, type XI, alpha 1
LOC101928436	chr1	103960700	104068105	107406	uncharacterized LOC101928436
RNPC3	chr1	104068577	104097859	29283	RNA-binding region (RNP1, RRM) containing 3
ACTG1P4	chr1	104112025	104114008	1984	actin, gamma 1 pseudogene 4
AMY2B	chr1	104078747	104153092	74346	amylase, alpha 2B (pancreatic)
AMY2A	chr1	104159998	104168400	8403	amylase, alpha 2A (pancreatic)
AMY1A	chr1	104198301	104207173	8873	amylase, alpha 1A (salivary)
AMY1B	chr1	104230040	104238889	8850	amylase, alpha 1B (salivary)
AMY1A	chr1	104230039	104239073	9035	amylase, alpha 1A (salivary)
AMY2A	chr1	104257374	104262492	5119	amylase, alpha 2A (pancreatic)
AMY1C	chr1	104292440	104301310	8871	amylase, alpha 1C (salivary)
LOC10012913ɛ	chr1	104615644	104619693	4050	THAP domain containing, apoptosis associated protein 3 pseudogene
LOC101928476	chr1	106132315	106161557	29243	uncharacterized LOC101928476
BC043293	chr1	106144773	106161557	16785	
PRMT6	chr1	107599266	107601916	2651	protein arginine methyltransferase 6
NTNG1	chr1	107682628	108024475	341848	netrin G1
VAV3	chr1	108113781	108507545	393765	vav 3 guanine nucleotide exchange factor
MIR7852	chr1	108439844	108439926	83	microRNA 7852
VAV3-AS1	chr1	108507064	108537229	30166	VAV3 antisense RNA 1
SLC25A24	chr1	108677343	108742980	65638	solute carrier family 25 (mitochondrial carrier; phosphate carrier), member 24
NBPF4	chr1	108765962	108786703	20742	neuroblastoma breakpoint family, member 4
BC051808	chr1	108803819	108816311	12493	
NBPF4	chr1	108918459	108953434	34976	neuroblastoma breakpoint family, member 4
NBPF6	chr1	108992903	109013260	20358	neuroblastoma breakpoint family, member 6
FAM102B	chr1	109102970	109181949	78980	family with sequence similarity 102, member B
HENMT1	chr1	109190909	109204148	13240	HEN1 methyltransferase homolog 1 (Arabidopsis)
PRPF38B	chr1	109234931	109244422	9492	pre-mRNA processing factor 38B
FNDC7	chr1	109255555	109285367	29813	fibronectin type III domain containing 7
STXBP3	chr1	109289284	109352148	62865	syntaxin binding protein 3
SPATA42	chr1	109399838	109401146	1309	spermatogenesis associated 42 (non-protein coding)
AKNAD1	chr1	109358519	109506121	147603	AKNA domain containing 1
GPSM2	chr1	109419602	109476957	57356	G-protein signaling modulator 2
CLCC1	chr1	109472129	109506121	33993	chloride channel CLIC-like 1
WDR47	chr1	109512837	109584850	72014	WD repeat domain 47
TAF13	chr1	109606997	109618624	11628	TAF13 RNA polymerase II, TATA box binding protein (TBP)-associated factor, 18kDa
MYBPHL	chr1	109834986	109849663	14678	myosin binding protein H-like

SORT1	chr1	109852187	109940563	88377	sortilin 1
PSMA5	chr1	109941652	109969108	27457	proteasome (prosome, macropain) subunit, alpha type, 5
FW340027	chr1	109965120	109966952	1833	
SYPL2	chr1	110009099	110024764	15666	synaptophysin-like 2
ATXN7L2	chr1	110026560	110035420	8861	ataxin 7-like 2
CYB561D1	chr1	110036657	110043063	6407	cytochrome b561 family, member D1
AMIGO1	chr1	110049445	110052336	2892	adhesion molecule with Ig-like domain 1
GPR61	chr1	110082493	110088455	5963	G protein-coupled receptor 61
GNAI3	chr1	110091185	110138454	47270	guanine nucleotide binding protein (G protein), alpha inhibiting activity polypeptide 3
MIR197	chr1	110141514	110141589	76	microRNA 197
GNAT2	chr1	110145888	110155705	9818	guanine nucleotide binding protein (G protein), alpha transducing activity polypeptide 2
AMPD2	chr1	110162125	110174677	12553	adenosine monophosphate deaminase 2
GSTM4	chr1	110198697	110208123	9427	glutathione S-transferase mu 4
GSTM2	chr1	110198697	110226619	27923	glutathione S-transferase mu 2 (muscle)
GSTM1	chr1	110230417	110236367	5951	glutathione S-transferase mu 1
GSTM5	chr1	110254863	110260890	6028	glutathione S-transferase mu 5
GSTM3	chr1	110276553	110283660	7108	glutathione S-transferase mu 3 (brain)
EPS8L3	chr1	110292701	110306644	13944	EPS8-like 3
KCNC4	chr1	110753335	110776674	23340	potassium voltage-gated channel, Shaw-related subfamily, member 4
SNORA25	chr1	110815105	110815229	125	small nucleolar RNA, H/ACA box 25
LOC440600	chr1	110828998	110881793	52796	uncharacterized LOC440600
RBM15	chr1	110881944	110889303	7360	RNA binding motif protein 15
BC069739	chr1	110887099	110888734	1636	
SLC16A4	chr1	110905472	110933704	28233	solute carrier family 16, member 4
LAMTOR5	chr1	110943876	110950546	6671	late endosomal/lysosomal adaptor, MAPK and MTOR activator 5
LAMTOR5-AS1	chr1	110950430	110958896	8467	LAMTOR5 antisense RNA 1
PROK1	chr1	110993787	110999976	6190	prokineticin 1
CYMP	chr1	111016377	111033891	17515	chymosin pseudogene
LOC440602	chr1	111030301	111032880	2580	uncharacterized LOC440602
KCNA10	chr1	111059838	111061797	1960	potassium voltage-gated channel, shaker-related subfamily, member 10
KCNA2	chr1	111136201	111174096	37896	potassium voltage-gated channel, shaker-related subfamily, member 2
KCNA3	chr1	111196185	111217655	21471	potassium voltage-gated channel, shaker-related subfamily, member 3
CD53	chr1	111413820	111442558	28739	CD53 molecule
LRIF1	chr1	111489811	111506566	16756	ligand dependent nuclear receptor interacting factor 1
DRAM2	chr1	111659953	111682838	22886	DNA-damage regulated autophagy modulator 2
CEPT1	chr1	111682248	111727724	45477	choline/ethanolamine phosphotransferase 1
DENND2D	chr1	111728590	111747160	18571	DENN/MADD domain containing 2D
CHI3L2	chr1	111770280	111786062	15783	chitinase 3-like 2
CHIAP2	chr1	111823145	111828730	5586	chitinase, acidic pseudogene 2
CHIA	chr1	111833473	111863188	29716	chitinase, acidic
PIFO	chr1	111889181	111895639	6459	primary cilia formation
PGCP1	chr1	111927140	111932473	5334	progastricsin (pepsinogen C) pseudogene 1
OVGP1	chr1	111956936	111970399	13464	oviductal glycoprotein 1, 120kDa
WDR77	chr1	111982511	111991830	9320	WD repeat domain 77
ATP5F1	chr1	111991461	112004525	13065	ATP synthase, H+ transporting, mitochondrial Fo complex, subunit B1
C1orf162	chr1	112016490	112021134	4645	chromosome 1 open reading frame 162
ADORA3	chr1	112025969	112106597	80629	adenosine A3 receptor
LINC01160	chr1	112141628	112150940	9313	long intergenic non-protein coding RNA 1160
LOC10012926(	chr1	112141628	112150940	9313	long intergenic non-protein coding RNA 1160
RAP1A	chr1	112085054	112259317	174264	RAP1A, member of RAS oncogene family
AK023457	chr1	112256829	112259310	2482	
FAM212B	chr1	112264685	112298419	33735	family with sequence similarity 212, member B
FAM212B-AS1	chr1	112282462	112290420	7959	FAM212B antisense RNA 1
LOC10050634(	chr1	112282462	112290420	7959	FAM212B antisense RNA 1
BC041890	chr1	112287934	112298131	10198	
LOC10192871(	chr1	112287938	112298131	10194	uncharacterized LOC101928718
DDX20	chr1	112298189	112310199	12011	DEAD (Asp-Glu-Ala-Asp) box polypeptide 20
KCND3-IT1	chr1	112396383	112399527	3145	KCND3 intronic transcript 1 (non-protein coding)
KCND3	chr1	112318453	112531777	213325	potassium voltage-gated channel, Shal-related subfamily, member 3
LOC643355	chr1	112533189	112541463	8275	uncharacterized LOC643355
SnoU13	chr1	112913625	112913729	105	
AX747733	chr1	112938880	112941439	2560	
CTTNBP2NL	chr1	112938799	113003786	64988	CTTNBP2 N-terminal like
DKFZp547A02(	chr1	112999439	113006065	6627	
MIR4256	chr1	113004391	113004455	65	microRNA 4256
WNT2B	chr1	113009162	113063910	54749	wingless-type MMTV integration site family, member 2B
ST7L	chr1	113066140	113162405	96266	suppression of tumorigenicity 7 like
CAPZA1	chr1	113162074	113214241	52168	capping protein (actin filament) muscle Z-line, alpha 1
MOV10	chr1	113216933	113243368	26436	Mov10 RISC complex RNA helicase
RHOC	chr1	113243748	113250025	6278	ras homolog family member C
PPM1J	chr1	113252615	113257950	5336	protein phosphatase, Mg2+/Mn2+ dependent, 1J
FAM19A3	chr1	113263188	113269856	6669	family with sequence similarity 19 (chemokine (C-C motif)-like), member A3
LINC01356	chr1	113362790	113393265	30476	long intergenic non-protein coding RNA 1356
AX748125	chr1	113362795	113393265	30471	
HP08777	chr1	113392521	113420491	27971	
AKR7A2P1	chr1	113465971	113467295	1325	aldo-keto reductase family 7, member A2 pseudogene 1
SLC16A1	chr1	113454469	113498975	44507	solute carrier family 16 (monocarboxylate transporter), member 1
BC023568	chr1	113499036	113506690	7655	
SLC16A1-AS1	chr1	113499036	113506690	7655	SLC16A1 antisense RNA 1
BX648855	chr1	113499070	113511603	12534	

BC047723	chr1	113499036	113542118	43083	
BC037540	chr1	113554308	113615724	61417	
LOC100996251	chr1	113554308	113615724	61417	uncharacterized LOC100996251
LRIG2	chr1	113615830	113667342	51513	leucine-rich repeats and immunoglobulin-like domains 2
LOC643441	chr1	113739403	113748875	9473	uncharacterized LOC643441
MAGI3	chr1	113933474	114228545	295072	membrane associated guanylate kinase, WW and PDZ domain containing 3
PHTF1	chr1	114239823	114302098	62276	putative homeodomain transcription factor 1
RSBN1	chr1	114304453	114355070	50618	round spermatid basic protein 1
PTPN22	chr1	114356432	114414375	57944	protein tyrosine phosphatase, non-receptor type 22 (lymphoid)
AP4B1-AS1	chr1	114355233	114443859	88627	AP4B1 antisense RNA 1
BCL2L15	chr1	114419435	114430169	10735	BCL2-like 15
AP4B1	chr1	114437370	114447741	10372	adaptor-related protein complex 4, beta 1 subunit
DCLRE1B	chr1	114447914	114456708	8795	DNA cross-link repair 1B
BC048113	chr1	114466622	114471854	5233	
BC036361	chr1	114466622	114471880	5259	
HIPK1-AS1	chr1	114466622	114472147	5526	HIPK1 antisense RNA 1
HIPK1	chr1	114471995	114520491	48497	homeodomain interacting protein kinase 1
OLFML3	chr1	114522012	114524876	2865	olfactomedin-like 3
SYT6	chr1	114631913	114696472	64560	synaptotagmin VI
TRIM33	chr1	114935398	115053781	118384	tripartite motif containing 33
BCAS2	chr1	115110180	115124265	14086	breast carcinoma amplified sequence 2
DKFZp686N16:	chr1	115125468	115126223	756	
DENND2C	chr1	115064016	115213044	149029	DENN/MADD domain containing 2C
AMPD1	chr1	115215719	115238239	22521	adenosine monophosphate deaminase 1
NRAS	chr1	115247084	115259515	12432	neuroblastoma RAS viral (v-ras) oncogene homolog
CSDE1	chr1	115259533	115300671	41139	cold shock domain containing E1, RNA-binding
SIKE1	chr1	115312104	115323308	11205	suppressor of IKBKE 1
SYCP1	chr1	115397423	115537990	140568	synaptonemal complex protein 1
HIST2H2BC	chr1	149821758	149822340	583	histone cluster 2, H2bc (pseudogene)
HIST2H2AA4	chr1	149822627	149823161	535	histone cluster 2, H2aa4
HIST2H2BE	chr1	149856009	149858232	2224	histone cluster 2, H2be
HIST2H2AC	chr1	149858524	149858961	438	histone cluster 2, H2ac
HIST2H2AB	chr1	149859018	149859466	449	histone cluster 2, H2ab
BOLA1	chr1	149871154	149872348	1195	bolA family member 1
SV2A	chr1	149874871	149889434	14564	synaptic vesicle glycoprotein 2A
SF3B4	chr1	149895208	149900144	4937	splicing factor 3b, subunit 4, 49kDa
MTMR11	chr1	149900542	149908791	8250	myotubularin related protein 11
OTUD7B	chr1	149912228	149982686	70459	OTU deubiquitinase 7B
TRNA_Ala	chr1	150017381	150017452	72	
VPS45	chr1	150039341	150117505	78165	vacuolar protein sorting 45 homolog (S. cerevisiae)
PLEKHO1	chr1	150121766	150131841	10076	pleckstrin homology domain containing, family O member 1
ANP32E	chr1	150190716	150208504	17789	acidic (leucine-rich) nuclear phosphoprotein 32 family, member E
CA14	chr1	150230217	150237480	7264	carbonic anhydrase XIV
APH1A	chr1	150237798	150241609	3812	APH1A gamma secretase subunit
C1orf54	chr1	150244686	150253335	8650	chromosome 1 open reading frame 54
CIART	chr1	150254942	150259504	4563	circadian associated repressor of transcription
C1orf51	chr1	150254952	150259501	4550	circadian associated repressor of transcription
MRPS21	chr1	150266261	150281414	15154	mitochondrial ribosomal protein S21
PRPF3	chr1	150293927	150325704	31778	pre-mRNA processing factor 3
RPRD2	chr1	150336586	150449041	112456	regulation of nuclear pre-mRNA domain containing 2
MIR6878	chr1	150464820	150464886	67	microRNA 6878
TARS2	chr1	150459839	150480085	20247	threonyl-tRNA synthetase 2, mitochondrial (putative)
ECM1	chr1	150480486	150486265	5780	extracellular matrix protein 1
FALEC	chr1	150488232	150490508	2277	focally amplified long non-coding RNA in epithelial cancer
LINC00568	chr1	150488232	150490508	2277	
MIR4257	chr1	150524404	150524490	87	microRNA 4257
ADAMTSL4	chr1	150521844	150533412	11569	ADAMTS-like 4
ADAMTSL4-AS	chr1	150533370	150547028	13659	ADAMTSL4 antisense RNA 1
MCL1	chr1	150547026	150552214	5189	myeloid cell leukemia 1
ENSA	chr1	150594598	150602098	7501	endosulfine alpha
GOLPH3L	chr1	150618700	150669672	50973	golgi phosphoprotein 3-like
HORMAD1	chr1	150670534	150693364	22831	HORMA domain containing 1
CTSS	chr1	150702671	150738433	35763	cathepsin S
CTSK	chr1	150768683	150780917	12235	cathepsin K
ARNT	chr1	150782180	150849244	67065	aryl hydrocarbon receptor nuclear translocator
SETDB1	chr1	150898814	150937220	38407	SET domain, bifurcated 1
CERS2	chr1	150937648	150947479	9832	ceramide synthase 2
ANXA9	chr1	150954498	150968114	13617	annexin A9
FAM63A	chr1	150969300	150980854	11555	family with sequence similarity 63, member A
PRUNE	chr1	150980972	151008189	27218	prune exopolyphosphatase
BNIPL	chr1	151009028	151020076	11049	BCL2/adenovirus E1B 19kD interacting protein like
C1orf56	chr1	151020186	151023871	3686	chromosome 1 open reading frame 56
CDC42SE1	chr1	151023446	151032125	8680	CDC42 small effector 1
MLLT11	chr1	151032150	151040973	8824	myeloid/lymphoid or mixed-lineage leukemia (trithorax homolog, Drosophila); translocated to, 11
GABPB2	chr1	151043079	151091007	47929	GA binding protein transcription factor, beta subunit 2
SEMA6C	chr1	151104162	151119140	14979	sema domain, transmembrane domain (TM), and cytoplasmic domain, (semaphorin) 6C
TNFAIP8L2	chr1	151129104	151132225	3122	tumor necrosis factor, alpha-induced protein 8-like 2
LYSMD1	chr1	151132223	151138424	6202	LysM, putative peptidoglycan-binding, domain containing 1
TNFAIP8L2-SC	chr1	151129104	151142773	13670	TNFAIP8L2-SCNM1 readthrough
SCNM1	chr1	151138497	151142773	4277	sodium channel modifier 1

TMOD4	chr1	151142462	151148547	6086	tropomodulin 4 (muscle)
VPS72	chr1	151148775	151162689	13915	vacuolar protein sorting 72 homolog (S. cerevisiae)
PIP5K1A	chr1	151171020	151222007	50988	phosphatidylinositol-4-phosphate 5-kinase, type I, alpha
PSMD4	chr1	151227196	151239954	12759	proteasome (prosome, macropain) 26S subunit, non-ATPase, 4
BC021024	chr1	151252499	151254405	1907	
ZNF687	chr1	151254093	151264381	10289	zinc finger protein 687
HV592965	chr1	151265261	151265284	24	
HV592900	chr1	151265461	151265487	27	
HV592953	chr1	151266582	151266606	25	
HV592909	chr1	151271387	151271414	28	
HV592934	chr1	151271499	151271523	25	
HV592964	chr1	151274383	151274409	27	
HV592982	chr1	151278732	151278766	35	
PI4KB	chr1	151264272	151300191	35920	phosphatidylinositol 4-kinase, catalytic, beta
RFX5	chr1	151313115	151319769	6655	regulatory factor X, 5 (influences HLA class II expression)
SELENBP1	chr1	151336777	151345210	8434	selenium binding protein 1
PSMB4	chr1	151372040	151374412	2373	proteasome (prosome, macropain) subunit, beta type, 4
POGZ	chr1	151375199	151431941	56743	pogo transposable element with ZNF domain
CGN	chr1	151483861	151511167	27307	cingulin
MIR554	chr1	151518271	151518367	97	microRNA 554
TUFT1	chr1	151512780	151556059	43280	tuftelin 1
SNX27	chr1	151584540	151671559	87020	sorting nexin family member 27
KIAA0488	chr1	151668703	151671551	2849	hypothetical protein LOC284484
THEM4	chr1	151843342	151882361	39020	thioesterase superfamily member 4
S100A10	chr1	151955385	151966714	11330	S100 calcium binding protein A10
AC2	chr1	151967006	152015250	48245	adenylate cyclase 2 (brain)
NBPF18P	chr1	151990753	151994985	4233	neuroblastoma breakpoint family, member 18 (pseudogene)
S100A11	chr1	152004981	152009511	4531	S100 calcium binding protein A11
TCHHL1	chr1	152056619	152061540	4922	trichohyalin-like 1
TCHH	chr1	152078792	152117706	38915	trichohyalin
RPTN	chr1	152126070	152131704	5635	repetin
INTS3	chr1	153700548	153746555	46008	integrator complex subunit 3
SLC27A3	chr1	153747767	153752633	4867	solute carrier family 27 (fatty acid transporter), member 3
GATAD2B	chr1	153777202	153895451	118250	GATA zinc finger domain containing 2B
DENND4B	chr1	153901976	153919154	17179	DENN/MADD domain containing 4B
CRTC2	chr1	153920147	153931132	10986	CREB regulated transcription coactivator 2
MIR6737	chr1	153934826	153934896	71	microRNA 6737
SLC39A1	chr1	153931574	153940660	9087	solute carrier family 39 (zinc transporter), member 1
CREB3L4	chr1	153940314	153946840	6527	cAMP responsive element binding protein 3-like 4
JTB	chr1	153946744	153950451	3708	jumping translocation breakpoint
RAB13	chr1	153954092	153958853	4762	RAB13, member RAS oncogene family
RPS27	chr1	153963238	153964631	1394	ribosomal protein S27
NUP210L	chr1	153965167	154127592	162426	nucleoporin 210kDa-like
TPM3	chr1	154127779	154164611	36833	tropomyosin 3
MIR190B	chr1	154166140	154166219	80	microRNA 190b
C1orf189	chr1	154171561	154178841	7281	chromosome 1 open reading frame 189
C1orf43	chr1	154179176	154193273	14098	chromosome 1 open reading frame 43
UBAP2L	chr1	154192647	154243986	51340	ubiquitin associated protein 2-like
SNORA58	chr1	154232202	154232338	137	small nucleolar RNA, H/ACA box 58
HAX1	chr1	154245038	154248355	3318	HCLS1 associated protein X-1
AQP10	chr1	154293591	154297801	4211	aquaporin 10
ATP8B2	chr1	154298035	154323780	25746	ATPase, aminophospholipid transporter, class I, type 8B, member 2
IL6R	chr1	154377668	154441926	64259	interleukin 6 receptor
SHE	chr1	154451953	154474526	22574	Src homology 2 domain containing E
TDRD10	chr1	154474694	154520623	45930	tudor domain containing 10
UBE2Q1	chr1	154521050	154531120	10071	ubiquitin-conjugating enzyme E2Q family member 1
UBE2Q1-AS1	chr1	154526084	154527504	1421	UBE2Q1 antisense RNA 1
CHRNB2	chr1	154540256	154552353	12098	cholinergic receptor, nicotinic, beta 2 (neuronal)
ADAR	chr1	154554533	154600456	45924	adenosine deaminase, RNA-specific
KCNN3	chr1	154669941	154842754	172814	potassium intermediate/small conductance calcium-activated channel, subfamily N, member 3
SHC1	chr1	154934773	154946959	12187	SHC (Src homology 2 domain containing) transforming protein 1
MIR4258	chr1	154948168	154948259	92	microRNA 4258
CKS1B	chr1	154947117	154951725	4609	CDC28 protein kinase regulatory subunit 1B
FLAD1	chr1	154955769	154965587	9819	flavin adenine dinucleotide synthetase 1
LENEP	chr1	154966061	154966791	731	lens epithelial protein
Mir_324	chr1	154979449	154979509	61	
ZBTB7B	chr1	154975105	154991001	15897	zinc finger and BTB domain containing 7B
DCST2	chr1	154991002	155006257	15256	DC-STAMP domain containing 2
DCST1	chr1	155006281	155023406	17126	DC-STAMP domain containing 1
LOC100505666	chr1	155017667	155036467	18801	uncharacterized LOC100505666
ADAM15	chr1	155023421	155035252	11832	ADAM metallopeptidase domain 15
EFNA4	chr1	155036212	155042029	5818	ephrin-A4
EFNA3	chr1	155036212	155060014	23803	ephrin-A3
EFNA1	chr1	155100348	155107386	7039	ephrin-A1
AK308965	chr1	155105263	155106131	869	
SLC50A1	chr1	155107819	155111334	3516	solute carrier family 50 (sugar efflux transporter), member 1
DPM3	chr1	155112366	155112996	631	dolichyl-phosphate mannosyltransferase polypeptide 3
KRTCAP2	chr1	155141883	155145804	3922	keratinocyte associated protein 2
TRIM46	chr1	155145420	155157447	12028	tripartite motif containing 46
AX746485	chr1	155155666	155157427	1762	

MUC1	chr1	155158299	155162706	4408	mucin 1, cell surface associated
FAM189B	chr1	155216995	155225274	8280	family with sequence similarity 189, member B
SCAMP3	chr1	155225769	155232176	6408	secretory carrier membrane protein 3
CLK2	chr1	155232658	155247501	14844	CDC-like kinase 2
HCN3	chr1	155247217	155259639	12423	hyperpolarization activated cyclic nucleotide-gated potassium channel 3
PKLR	chr1	155259083	155271225	12143	pyruvate kinase, liver and RBC
FDPS	chr1	155278538	155290457	11920	farnesyl diphosphate synthase
RUSC1-AS1	chr1	155286653	155293938	7286	RUSC1 antisense RNA 1
RUSC1	chr1	155290639	155300909	10271	RUN and SH3 domain containing 1
MIR555	chr1	155316140	155316236	97	microRNA 555
POU5F1P4	chr1	155402970	155404053	1084	POU domain class 5, transcription factor 1 pseudogene 4
ASH1L	chr1	155305051	155532585	227535	ash1 (absent, small, or homeotic)-like (Drosophila)
ASH1L-AS1	chr1	155531771	155533735	1965	ASH1L antisense RNA 1
BC017347	chr1	155531863	155533735	1873	
MSTO2P	chr1	155579978	155584616	4639	misato family member 2, pseudogene
MSTO1	chr1	155579960	155584758	4799	misato 1, mitochondrial distribution and morphology regulator
BC041646	chr1	155596545	155618335	21791	
DAP3	chr1	155658881	155708800	49920	death associated protein 3
YY1AP1	chr1	155629232	155755165	125934	YY1 associated protein 1
MSTO2P	chr1	155715558	155720673	5116	misato family member 2, pseudogene
GON4L	chr1	155719448	155829191	109744	gon-4-like (C. elegans)
SYT11	chr1	155829259	155854990	25732	synaptotagmin XI
RIT1	chr1	155867598	155881193	13596	Ras-like without CAAX 1
SNORA42	chr1	155889699	155889833	135	small nucleolar RNA, H/ACA box 80E
SNORA80E	chr1	155889699	155889833	135	small nucleolar RNA, H/ACA box 80E
KIAA0907	chr1	155882835	155904188	21354	KIAA0907
SCARNA4	chr1	155895748	155895877	130	small Cajal body-specific RNA 4
RXFP4	chr1	155911479	155912625	1147	relaxin/insulin-like family peptide receptor 4
MIR6738	chr1	155921063	155921127	65	microRNA 6738
ARHGEF2	chr1	155916629	155959864	43236	Rho/Rac guanine nucleotide exchange factor (GEF) 2
SSR2	chr1	155978838	155990758	11921	signal sequence receptor, beta (translocon-associated protein beta)
UBQLN4	chr1	156005091	156023516	18426	ubiquilin 4
LAMTOR2	chr1	156024516	156028301	3786	late endosomal/lysosomal adaptor, MAPK and MTOR activator 2
RAB25	chr1	156030965	156040295	9331	RAB25, member RAS oncogene family
MEX3A	chr1	156041803	156051789	9987	mex-3 RNA binding family member A
MIR7851	chr1	155996957	156132001	135045	microRNA 7851
LMNA	chr1	156052336	156109880	57545	lamin A/C
SEMA4A	chr1	156119734	156147542	27809	sema domain, immunoglobulin domain (Ig), transmembrane domain (TM) and short cytoplasmic domain, (s
SLC25A44	chr1	156163722	156182587	18866	solute carrier family 25, member 44
PMF1	chr1	156182778	156209868	27091	polyamine-modulated factor 1
PMF1-BGLAP	chr1	156182778	156213123	30346	PMF1-BGLAP readthrough
BGLAP	chr1	156211950	156213123	1174	bone gamma-carboxyglutamate (gla) protein
PAQR6	chr1	156213111	156217908	4798	progestin and adipoQ receptor family member VI
SMG5	chr1	156219014	156252620	33607	SMG5 nonsense mediated mRNA decay factor
TMEM79	chr1	156252703	156262234	9532	transmembrane protein 79
AF086351	chr1	156259881	156262810	2930	
C1orf85	chr1	156262477	156265480	3004	chromosome 1 open reading frame 85
VHLL	chr1	156268414	156269428	1015	von Hippel-Lindau tumor suppressor-like
CCT3	chr1	156278751	156308206	29456	chaperonin containing TCP1, subunit 3 (gamma)
TSACC	chr1	156307104	156316785	9682	TSSK6 activating co-chaperone
RHBG	chr1	156338979	156355013	16035	Rh family, B glycoprotein (gene/pseudogene)
BC016978	chr1	156374048	156377645	3598	
C1orf61	chr1	156374054	156400493	26440	chromosome 1 open reading frame 61
MIR9-1	chr1	156390132	156390221	90	microRNA 9-1
MEF2D	chr1	156433512	156470634	37123	myocyte enhancer factor 2D
IQGAP3	chr1	156495196	156542396	47201	IQ motif containing GTPase activating protein 3
TTC24	chr1	156549518	156556562	7045	tetratricopeptide repeat domain 24
APOA1BP	chr1	156561557	156566601	5045	apolipoprotein A-I binding protein
GPATCH4	chr1	156564099	156571279	7181	G patch domain containing 4
HAPLN2	chr1	156589085	156595517	6433	hyaluronan and proteoglycan link protein 2
BC005081	chr1	156611456	156613427	1972	
BCAN	chr1	156611739	156629324	17586	brevican
NES	chr1	156638554	156647189	8636	nestin
CRABP2	chr1	156669399	156675608	6210	cellular retinoic acid binding protein 2
ISG20L2	chr1	156692412	156698211	5800	interferon stimulated exonuclease gene 20kDa-like 2
RRNAD1	chr1	156698262	156706752	8491	ribosomal RNA adenine dimethylase domain containing 1
MRPL24	chr1	156707093	156710923	3831	mitochondrial ribosomal protein L24
HDGF	chr1	156711898	156736717	24820	hepatoma-derived growth factor
PRCC	chr1	156737273	156770609	33337	papillary renal cell carcinoma (translocation-associated)
SH2D2A	chr1	156776034	156786640	10607	SH2 domain containing 2A
NTRK1	chr1	156785541	156851642	66102	neurotrophic tyrosine kinase, receptor, type 1
INSRR	chr1	156810664	156828712	18049	insulin receptor-related receptor
PEAR1	chr1	156863522	156886226	22705	platelet endothelial aggregation receptor 1
ARHGEF11	chr1	156904631	157015162	110532	Rho guanine nucleotide exchange factor (GEF) 11
ETV3L	chr1	157061834	157069600	7767	ets variant 3-like
CYCSP52	chr1	157098153	157098463	311	cytochrome c, somatic pseudogene 52
ETV3	chr1	157094458	157108383	13926	ets variant 3
FCRL4	chr1	157543538	157567870	24333	Fc receptor-like 4
FCRL3	chr1	157646270	157670775	24506	Fc receptor-like 3
FCRL1	chr1	157764193	157789940	25748	Fc receptor-like 1

CD5L	chr1	157800703	157811634	10932	CD5 molecule-like
AK057438	chr1	157895763	157918861	23099	
LOC646268	chr1	158101833	158110430	8598	hCG1654703
CD1D	chr1	158149736	158156216	6481	CD1d molecule
AK097921	chr1	158169170	158173667	4498	
CD1A	chr1	158223926	158228058	4133	CD1a molecule
CD1C	chr1	158259562	158264564	5003	CD1c molecule
CD1B	chr1	158297739	158301321	3583	CD1b molecule
CD1E	chr1	158323253	158327343	4091	CD1e molecule
OR10T2	chr1	158368311	158369256	946	olfactory receptor, family 10, subfamily T, member 2
OR10K2	chr1	158389717	158390656	940	olfactory receptor, family 10, subfamily K, member 2
OR10K1	chr1	158435351	158436293	943	olfactory receptor, family 10, subfamily K, member 1
OR10R2	chr1	158449667	158450675	1009	olfactory receptor, family 10, subfamily R, member 2
AK057554	chr1	158444244	158464676	20433	
OR6Y1	chr1	158516917	158517895	979	olfactory receptor, family 6, subfamily Y, member 1
OR6P1	chr1	158532440	158533394	955	olfactory receptor, family 6, subfamily P, member 1
OR10X1	chr1	158548708	158549689	982	olfactory receptor, family 10, subfamily X, member 1
OR10Z1	chr1	158576228	158577170	943	olfactory receptor, family 10, subfamily Z, member 1
SPTA1	chr1	158580495	158656506	76012	spectrin, alpha, erythrocytic 1
OR6K2	chr1	158669467	158670442	976	olfactory receptor, family 6, subfamily K, member 2
OR6K3	chr1	158686957	158687905	949	olfactory receptor, family 6, subfamily K, member 3
OR6K6	chr1	158724605	158725637	1033	olfactory receptor, family 6, subfamily K, member 6
MNDA	chr1	158801167	158819270	18104	myeloid cell nuclear differentiation antigen
PYHIN1	chr1	158901336	158946849	45514	pyrin and HIN domain family, member 1
IFI16	chr1	158969757	159024945	55189	interferon, gamma-inducible protein 16
BC038194	chr1	159315958	159438858	122901	
OR10J1	chr1	159409511	159410600	1090	olfactory receptor, family 10, subfamily J, member 1
OR10J5	chr1	159504867	159505797	931	olfactory receptor, family 10, subfamily J, member 5
APCS	chr1	159557615	159558661	1047	amyloid P component, serum
CRP	chr1	159682078	159684379	2302	C-reactive protein, pentraxin-related
VSIG8	chr1	159824105	159832447	8343	V-set and immunoglobulin domain containing 8
CCDC19	chr1	159842153	159869906	27754	cilia and flagella associated protein 45
CFAP45	chr1	159842153	159869906	27754	cilia and flagella associated protein 45
TAGLN2	chr1	159887896	159895332	7437	transgelin 2
IGSF9	chr1	159896828	159915386	18559	immunoglobulin superfamily, member 9
SLAMF9	chr1	159921281	159924044	2764	SLAM family member 9
LINC01133	chr1	159931013	159948876	17864	long intergenic non-protein coding RNA 1133
LOC100505633	chr1	159931013	159948876	17864	long intergenic non-protein coding RNA 1133
PIGM	chr1	159997461	160001783	4323	phosphatidylinositol glycan anchor biosynthesis, class M
KCNJ10	chr1	160007256	160040051	32796	potassium inwardly-rectifying channel, subfamily J, member 10
KCNJ9	chr1	160051359	160059212	7854	potassium inwardly-rectifying channel, subfamily J, member 9
IGSF8	chr1	160061128	160068618	7491	immunoglobulin superfamily, member 8
ATP1A2	chr1	160085519	160113374	27856	ATPase, Na+/K+ transporting, alpha 2 polypeptide
ATP1A4	chr1	160121351	160156767	35417	ATPase, Na+/K+ transporting, alpha 4 polypeptide
CASQ1	chr1	160160284	160171676	11393	calsequestrin 1 (fast-twitch, skeletal muscle)
AK093299	chr1	160171988	160178659	6672	
LOC729867	chr1	160171988	160178659	6672	uncharacterized LOC729867
PEA15	chr1	160175108	160185166	10059	phosphoprotein enriched in astrocytes 15
DCAF8	chr1	160185504	160254941	69438	DDB1 and CUL4 associated factor 8
PEX19	chr1	160246598	160254941	8344	peroxisomal biogenesis factor 19
COPA	chr1	160258376	160313354	54979	coatomer protein complex, subunit alpha
SUMO1P3	chr1	160287054	160288260	1207	SUMO1 pseudogene 3 (functional)
NCSTN	chr1	160313062	160328742	15681	nicastrin
NHLH1	chr1	160336860	160342638	5779	nescient helix loop helix 1
VANGL2	chr1	160370230	160398468	28239	VANGL planar cell polarity protein 2
SLAMF6	chr1	160454819	160493052	38234	SLAM family member 6
CD84	chr1	160510883	160549306	38424	CD84 molecule
SLAMF1	chr1	160579608	160617101	37494	signaling lymphocytic activation molecule family member 1
SLAMF7	chr1	160708846	160724608	15763	SLAM family member 7
LY9	chr1	160765863	160798045	32183	lymphocyte antigen 9
CD244	chr1	160799949	160832692	32744	CD244 molecule, natural killer cell receptor 2B4
ITLN1	chr1	160846329	160854960	8632	intelectin 1 (galactofuranose binding)
LOC101928372	chr1	160902254	160919712	17459	uncharacterized LOC101928372
ITLN2	chr1	160914815	160924589	9775	intelectin 2
F11R	chr1	160965000	161008774	43775	F11 receptor
TSTD1	chr1	161007421	161008774	1354	thiosulfate sulfurtransferase (rhodanese)-like domain containing 1
USF1	chr1	161009040	161015769	6730	upstream transcription factor 1
ARHGAP30	chr1	161016731	161039760	23030	Rho GTPase activating protein 30
KLHDC9	chr1	161068150	161070138	1989	kelch domain containing 9
PFDN2	chr1	161070345	161087866	17522	prefoldin subunit 2
NIT1	chr1	161087861	161095235	7375	nitrilase 1
DEDD	chr1	161090768	161102478	11711	death effector domain containing
UFC1	chr1	161123533	161128646	5114	ubiquitin-fold modifier conjugating enzyme 1
USP21	chr1	161129253	161135516	6264	ubiquitin-specific protease
PPOX	chr1	161136180	161147801	11622	protoporphyrinogen oxidase
B4GALT3	chr1	161141099	161147758	6660	UDP-Gal:betaGlcNAc beta 1,4- galactosyltransferase, polypeptide 3
ADAMTS4	chr1	161159537	161168845	9309	ADAM metallopeptidase with thrombospondin type 1 motif, 4
NDUFS2	chr1	161169104	161184184	15081	NADH dehydrogenase (ubiquinone) Fe-S protein 2, 49kDa (NADH-coenzyme Q reductase)
FCER1G	chr1	161185086	161189038	3953	Fc fragment of IgE, high affinity I, receptor for; gamma polypeptide
APOA2	chr1	161192082	161193418	1337	apolipoprotein A-II

MIR5187	chr1	161196975	161197051	77	microRNA 5187
NR1I3	chr1	161199455	161208000	8546	nuclear receptor subfamily 1, group I, member 3
TOMM40L	chr1	161195728	161213089	17362	translocase of outer mitochondrial membrane 40 homolog (yeast)-like
PCP4L1	chr1	161228516	161255240	26725	Purkinje cell protein 4 like 1
MPZ	chr1	161274524	161279762	5239	myelin protein zero
SDHC	chr1	161284165	161334535	50371	succinate dehydrogenase complex, subunit C, integral membrane protein, 15kDa
C1orf192	chr1	161334520	161337673	3154	chromosome 1 open reading frame 192
TRNA_Asp	chr1	161410614	161410686	73	
TRNA_Leu	chr1	161411322	161411405	84	
FCGR2A	chr1	161475204	161489360	14157	Fc fragment of IgG, low affinity IIa, receptor (CD32)
HSPA6	chr1	161494035	161496687	2653	heat shock 70kDa protein 6 (HSP70B')
TRNA_Leu	chr1	161500131	161500214	84	
TRNA_Asp	chr1	161501914	161501986	73	
FCGR3A	chr1	161511550	161520413	8864	Fc fragment of IgG, low affinity IIIa, receptor (CD16a)
FCGR2C	chr1	161551128	161571010	19883	Fc fragment of IgG, low affinity IIc, receptor for (CD32) (gene/pseudogene)
HSPA7	chr1	161575848	161578341	2494	heat shock 70kDa protein 7 (HSP70B)
FCGR3B	chr1	161592987	161601753	8767	Fc fragment of IgG, low affinity IIIb, receptor (CD16b)
FCGR2B	chr1	161632904	161648444	15541	Fc fragment of IgG, low affinity IIb, receptor (CD32)
RPL31P11	chr1	161653494	161655042	1549	ribosomal protein L31 pseudogene 11
FCRLA	chr1	161676761	161684142	7382	Fc receptor-like A
FCRLB	chr1	161691333	161697933	6601	Fc receptor-like B
DUSP12	chr1	161719580	161726952	7373	dual specificity phosphatase 12
ATF6	chr1	161736033	161933860	197828	activating transcription factor 6
MIR4654	chr1	162126896	162126972	77	microRNA 4654
NOS1AP	chr1	162039580	162339813	300234	nitric oxide synthase 1 (neuronal) adaptor protein
MIR556	chr1	162312335	162312430	96	microRNA 556
C1orf226	chr1	162348695	162356608	7914	chromosome 1 open reading frame 226
SH2D1B	chr1	162365055	162381928	16874	SH2 domain containing 1B
UHMK1	chr1	162466963	162499419	32457	U2AF homology motif (UHM) kinase 1
UAP1	chr1	162531295	162569633	38339	UDP-N-acetylglucosamine pyrophosphorylase 1
DDR2	chr1	162602227	162750247	148021	discoidin domain receptor tyrosine kinase 2
AF268386	chr1	162751900	162756362	4463	
HSD17B7	chr1	162760495	162782608	22114	hydroxysteroid (17-beta) dehydrogenase 7
C1orf110	chr1	162794247	162838605	44359	chromosome 1 open reading frame 110
RGS4	chr1	163038395	163046592	8198	regulator of G-protein signaling 4
LOC101928404	chr1	163131464	163182813	51350	uncharacterized LOC101928404
RGS5	chr1	163112088	163291581	179494	regulator of G-protein signaling 5
NUF2	chr1	163291722	163325553	33832	NUF2, NDC80 kinetochore complex component
LOC100422212	chr1	163390859	163392981	2123	eukaryotic translation initiation factor 3, subunit J pseudogene
AX748175	chr1	164595272	164651720	56449	
PBX1	chr1	164528596	164854300	325705	pre-B-cell leukemia homeobox 1
LOC100505795	chr1	164738352	164743878	5527	uncharacterized LOC100505795
LMX1A	chr1	165171103	165325952	154850	LIM homeobox transcription factor 1, alpha
RXRG	chr1	165370158	165414592	44435	retinoid X receptor, gamma
LOC400794	chr1	165446078	165551341	105264	uncharacterized LOC400794
LRRC52	chr1	165513477	165533185	19709	leucine rich repeat containing 52
MGST3	chr1	165600109	165625372	25264	microsomal glutathione S-transferase 3
ALDH9A1	chr1	165631448	165667900	36453	aldehyde dehydrogenase 9 family, member A1
LOC440700	chr1	165667986	165679199	11214	carbonic anhydrase XIV (CA14) pseudogene
BC071770	chr1	165738165	165744685	6521	
LOC100147773	chr1	165738165	165744685	6521	uncharacterized LOC100147773
TMCO1	chr1	165693527	165796992	103466	transmembrane and coiled-coil domains 1
FMO9P	chr1	166573152	166594473	21322	flavin containing monooxygenase 9 pseudogene
POGK	chr1	166808723	166823709	14987	pogo transposable element with KRAB domain
TADA1	chr1	166825748	166845654	19907	transcriptional adaptor 1
ILDR2	chr1	166882440	166944561	62122	immunoglobulin-like domain containing receptor 2
MAEL	chr1	166944818	166991449	46632	maelstrom spermatogenic transposon silencer
GPA33	chr1	167022081	167059868	37788	glycoprotein A33 (transmembrane)
DUSP27	chr1	167064086	167098402	34317	dual specificity phosphatase 27 (putative)
RBSG4	chr1	167144598	167165042	20445	
LINC01363	chr1	167144598	167165042	20445	long intergenic non-protein coding RNA 1363
POU2F1	chr1	167190065	167396582	206518	POU class 2 homeobox 1
CREG1	chr1	167510250	167523056	12807	cellular repressor of E1A-stimulated genes 1
RCSD1	chr1	167599473	167675486	76014	RCSD domain containing 1
TRNA_Pro	chr1	167683961	167684033	73	
TRNA_Pro	chr1	167684724	167684796	73	
MPZL1	chr1	167691186	167761156	69971	myelin protein zero-like 1
ADCY10	chr1	167778624	167883464	104841	adenylate cyclase 10 (soluble)
MPC2	chr1	167885912	167906307	20396	mitochondrial pyruvate carrier 2
DCAF6	chr1	167905796	168045083	139288	DDB1 and CUL4 associated factor 6
GPR161	chr1	168048779	168106905	58127	G protein-coupled receptor 161
TIPRL	chr1	168148082	168171351	23270	TOR signaling pathway regulator
SFT2D2	chr1	168195254	168212088	16835	SFT2 domain containing 2
ANKRD36BP1	chr1	168214818	168216668	1851	ankyrin repeat domain 36B pseudogene 1
DQ576756	chr1	168218461	168220378	1918	
TBX19	chr1	168195254	168283664	88411	T-box 19
LOC101928565	chr1	168433351	168464882	31532	uncharacterized LOC101928565
XCL2	chr1	168510002	168513235	3234	chemokine (C motif) ligand 2
XCL1	chr1	168545710	168551315	5606	chemokine (C motif) ligand 1
DPT	chr1	168664694	168698442	33749	dermatopontin

LINC00626	chr1	168756178	168762126	5949	long intergenic non-protein coding RNA 626
LINC00970	chr1	168873142	169056243	183102	long intergenic non-protein coding RNA 970
ATP1B1	chr1	169075946	169101960	26015	ATPase, Na+/K+ transporting, beta 1 polypeptide
NME7	chr1	169101767	169337201	235435	NME/NM23 family member 7
BLZF1	chr1	169337193	169365780	28588	basic leucine zipper nuclear factor 1
CCDC181	chr1	169364107	169429907	65801	coiled-coil domain containing 181
BC041451	chr1	169411875	169429907	18033	
SLC19A2	chr1	169433148	169455208	22061	solute carrier family 19 (thiamine transporter), member 2
F5	chr1	169481191	169555769	74579	coagulation factor V (proaccelerin, labile factor)
SELP	chr1	169558087	169599377	41291	selectin P (granule membrane protein 140kDa, antigen CD62)
SELL	chr1	169659805	169680843	21039	selectin L
SELE	chr1	169691780	169703220	11441	selectin E
C1orf112	chr1	169631244	169822229	190986	chromosome 1 open reading frame 112
METTL18	chr1	169761669	169764061	2393	methyltransferase like 18
SCYL3	chr1	169822214	169863100	40887	SCY1-like 3 (S. cerevisiae)
KIFAP3	chr1	169890469	170043879	153411	kinesin-associated protein 3
MIR3119-1	chr1	170120518	170120603	86	microRNA 3119-1
MIR3119-2	chr1	170120518	170120603	86	microRNA 3119-2
METTL11B	chr1	170115187	170136923	21737	methyltransferase like 11B
LINC01142	chr1	170240545	170253349	12805	long intergenic non-protein coding RNA 1142
LOC284688	chr1	170240545	170253349	12805	long intergenic non-protein coding RNA 1142
PRRX1	chr1	170633312	170708541	75230	paired related homeobox 1
MROH9	chr1	170904611	171033906	129296	maestro heat-like repeat family member 9
MIR1295A	chr1	171070868	171070947	80	microRNA 1295a
MIR1295B	chr1	171070879	171070939	61	microRNA 1295b
FMO3	chr1	171060017	171086959	26943	flavin containing monooxygenase 3
FMO6P	chr1	171106878	171130702	23825	flavin containing monooxygenase 6 pseudogene
FMO2	chr1	171154346	171181822	27477	flavin containing monooxygenase 2 (non-functional)
FMO1	chr1	171217609	171255117	37509	flavin containing monooxygenase 1
FMO4	chr1	171283485	171311223	27739	flavin containing monooxygenase 4
TOP1P1	chr1	171308034	171310463	2430	topoisomerase (DNA) I pseudogene 1
PRRC2C	chr1	171454665	171562650	107986	proline-rich coiled-coil 2C
MYOC	chr1	171604556	171621823	17268	myocilin, trabecular meshwork inducible glucocorticoid response
VAMP4	chr1	171669295	171711379	42085	vesicle-associated membrane protein 4
METTL13	chr1	171750760	171766856	16097	methyltransferase like 13
AK094818	chr1	171763732	171766852	3121	
DNM3	chr1	171810617	172387567	576951	dynamin 3
MIR3120	chr1	172107947	172108028	82	microRNA 3120
MIR214	chr1	172107937	172108047	111	microRNA 214
DNM3OS	chr1	172106018	172113975	7958	DNM3 opposite strand/antisense RNA
MIR199A2	chr1	172113674	172113784	111	microRNA 199a-2
PIGC	chr1	172362273	172413230	50958	phosphatidylinositol glycan anchor biosynthesis, class C
C1orf105	chr1	172389827	172437969	48143	chromosome 1 open reading frame 105
SUCO	chr1	172501488	172580975	79488	SUN domain containing ossification factor
TNFSF18	chr1	173010359	173020103	9745	tumor necrosis factor (ligand) superfamily, member 18
TNFSF4	chr1	173152869	173176452	23584	tumor necrosis factor (ligand) superfamily, member 4
LOC100506023	chr1	173204198	173446294	242097	uncharacterized LOC100506023
LOC101928673	chr1	173386927	173430501	43575	uncharacterized LOC101928673
BC136808	chr1	173387087	173430501	43415	
PRDX6	chr1	173446485	173457946	11462	peroxiredoxin 6
SLC9C2	chr1	173469603	173572233	102631	solute carrier family 9, member C2 (putative)
LOC730159	chr1	173604660	173606272	1613	uncharacterized LOC730159
ANKRD45	chr1	173577474	173639001	61528	ankyrin repeat domain 45
KLHL20	chr1	173684079	173755840	71762	kelch-like family member 20
CENPL	chr1	173768687	173793777	25091	centromere protein L
DARS2	chr1	173793796	173827682	33887	aspartyl-tRNA synthetase 2, mitochondrial
GAS5-AS1	chr1	173832385	173833079	695	GAS5 antisense RNA 1
SNORD81	chr1	173833312	173833355	44	small nucleolar RNA, C/D box 81
SNORD47	chr1	173833506	173833583	78	small nucleolar RNA, C/D box 47
SNORD80	chr1	173833970	173834041	72	small nucleolar RNA, C/D box 80
SNORD79	chr1	173834487	173834568	82	small nucleolar RNA, C/D box 79
SNORD78	chr1	173834770	173834824	55	small nucleolar RNA, C/D box 78
GAS5	chr1	173833038	173837125	4088	growth arrest-specific 5 (non-protein coding)
SNORD44	chr1	173835105	173835166	62	small nucleolar RNA, C/D box 44
SNORD77	chr1	173835448	173835509	62	small nucleolar RNA, C/D box 77
SNORD76	chr1	173835772	173835853	82	small nucleolar RNA, C/D box 76
SNORD75	chr1	173836016	173836076	61	small nucleolar RNA, C/D box 75
SNORD74	chr1	173836811	173836883	73	small nucleolar RNA, C/D box 74
ZBTB37	chr1	173837219	173855774	18556	zinc finger and BTB domain containing 37
DQ593451	chr1	173867684	173867712	29	
SERPINC1	chr1	173872941	173886516	13576	serpin peptidase inhibitor, clade C (antithrombin), member 1
RC3H1	chr1	173900221	173962210	61990	ring finger and CCCH-type domains 1
LOC102724601	chr1	174090775	174128425	37651	uncharacterized LOC102724601
GPR52	chr1	174417211	174418683	1473	G protein-coupled receptor 52
RABGAP1L	chr1	174128551	174964445	835895	RAB GTPase activating protein 1-like
LOC101928696	chr1	174904083	174923398	19316	uncharacterized LOC101928696
CACYBP	chr1	174968570	174981163	12594	calcyclin binding protein
MRPS14	chr1	174982093	174992591	10499	mitochondrial ribosomal protein S14
TNN	chr1	175036993	175117202	80210	tenascin N
TNR	chr1	175291934	175712752	420819	tenascin R

AK093214	chr1	175526123	175533005	6883	
BC043291	chr1	175526123	175537388	11266	
LOC101928751	chr1	175846478	175849600	3123	uncharacterized LOC101928751
SCARNA3	chr1	175937532	175937676	145	small Cajal body-specific RNA 3
RFWD2	chr1	175913961	176176380	262420	ring finger and WD repeat domain 2, E3 ubiquitin protein ligase
PAPPA2	chr1	176432306	176811970	379665	pappalysin 2
SEC16B	chr1	177897488	178007142	109655	SEC16 homolog B (S. cerevisiae)
LOC730102	chr1	177975274	178007142	31869	quinone oxidoreductase-like protein 2 pseudogene
RASAL2-AS1	chr1	178060642	178063128	2487	RASAL2 antisense RNA 1
RASAL2	chr1	178062863	178448648	385786	RAS protein activator like 2
TEX35	chr1	178482211	178517734	35524	testis expressed 35
C1orf220	chr1	178511930	178518024	6095	chromosome 1 open reading frame 220
MIR4424	chr1	178646883	178646969	87	microRNA 4424
RALGPS2	chr1	178694281	178890977	196697	Ral GEF with PH domain and SH3 binding motif 2
ANGPTL1	chr1	178818669	178840215	21547	angiopoietin-like 1
FAM20B	chr1	178995073	179045702	50630	family with sequence similarity 20, member B
TOR3A	chr1	179051111	179065129	14019	torsin family 3, member A
ABL2	chr1	179068461	179198819	130359	ABL proto-oncogene 2, non-receptor tyrosine kinase
DD413682	chr1	179170962	179170982	21	
SOAT1	chr1	179262848	179327814	64967	sterol O-acyltransferase 1
AXDND1	chr1	179334854	179523870	189017	axonemal dynein light chain domain containing 1
TDRD5	chr1	179560747	179660407	99661	tudor domain containing 5
FAM163A	chr1	179696914	179785333	88420	family with sequence similarity 163, member A
TOR1AIP2	chr1	179809101	179846941	37841	torsin A interacting protein 2
TOR1AIP1	chr1	179851176	179889212	38037	torsin A interacting protein 1
CEP350	chr1	179923907	180084015	160109	centrosomal protein 350kDa
QSOX1	chr1	180123967	180167169	43203	quiescin Q6 sulfhydryl oxidase 1
AX747393	chr1	180150089	180151749	1661	
FLJ23867	chr1	180167143	180169859	2717	uncharacterized protein FLJ23867
ACBD6	chr1	180257351	180472022	214672	acyl-CoA binding domain containing 6
MIR3121	chr1	180407448	180407525	78	microRNA 3121
BC036830	chr1	180528109	180535654	7546	
OVAAL	chr1	180528109	180535654	7546	ovarian adenocarcinoma amplified long non-coding RNA
XPR1	chr1	180601145	180859415	258271	xenotropic and polytropic retrovirus receptor 1
LOC101928973	chr1	181143619	181151342	7724	uncharacterized LOC101928973
BC039493	chr1	181143619	181151342	7724	
GM140	chr1	181205523	181207740	2218	uncharacterized LOC100287948
AF387615	chr1	181382578	181382716	139	
CACNA1E	chr1	181452685	181775921	323237	calcium channel, voltage-dependent, R type, alpha 1E subunit
ZNF648	chr1	182023704	182030847	7144	zinc finger protein 648
LINC01344	chr1	182173079	182283196	110118	long intergenic non-protein coding RNA 1344
GLUL	chr1	182350838	182361341	10504	glutamate-ammonia ligase
JA429801	chr1	182360854	182360875	22	
JA429802	chr1	182360900	182360923	24	
TEDDM1	chr1	182367251	182369751	2501	transmembrane epididymal protein 1
LINC00272	chr1	182376755	182383948	7194	long intergenic non-protein coding RNA 272
RGSL1	chr1	182419255	182529732	110478	regulator of G-protein signaling like 1
RNASEL	chr1	182542768	182558394	15627	ribonuclease L (2',5'-oligoisoadenylate synthetase-dependent)
RGS16	chr1	182567757	182573548	5792	regulator of G-protein signaling 16
LOC284648	chr1	182584274	182585764	1491	uncharacterized LOC284648
RGS8	chr1	182615791	182642067	26277	regulator of G-protein signaling 8
NPL	chr1	182758583	182799519	40937	N-acetylneuraminate pyruvate lyase (dihydrodipicolinate synthase)
DHX9	chr1	182808438	182857117	48680	DEAH (Asp-Glu-Ala-His) box helicase 9
SHCBP1L	chr1	182868999	182922553	53555	SHC SH2-domain binding protein 1-like
LAMC1	chr1	182992594	183114727	122134	laminin, gamma 1 (formerly LAMB2)
LAMC2	chr1	183155173	183214262	59090	laminin, gamma 2
NMNAT2	chr1	183217371	183387634	170264	nicotinamide nucleotide adenylyltransferase 2
SMG7-AS1	chr1	183430010	183441117	11108	SMG7 antisense RNA 1
SMG7	chr1	183441505	183523328	81824	SMG7 nonsense mediated mRNA decay factor
APOBEC4	chr1	183615410	183622448	7039	apolipoprotein B mRNA editing enzyme, catalytic polypeptide-like 4 (putative)
RGL1	chr1	183605181	183897685	292505	ral guanine nucleotide dissociation stimulator-like 1
COLGALT2	chr1	183898795	184006878	108084	collagen beta(1-O)galactosyltransferase 2
TSEN15	chr1	184020784	184043344	22561	TSEN15 tRNA splicing endonuclease subunit
C1orf21	chr1	184356149	184598155	242007	chromosome 1 open reading frame 21
EDEM3	chr1	184659624	184724041	64418	ER degradation enhancer, mannosidase alpha-like 3
FAM129A	chr1	184760158	184943718	183561	family with sequence similarity 129, member A
RNF2	chr1	185014550	185071740	57191	ring finger protein 2
TRMT1L	chr1	185087217	185126230	39014	tRNA methyltransferase 1 homolog (S. cerevisiae)-like
SWT1	chr1	185126191	185260913	134723	SWT1 RNA endoribonuclease homolog (S. cerevisiae)
JA611272	chr1	185220508	185220534	27	
IVNS1ABP	chr1	185265521	185286461	20941	influenza virus NS1A binding protein
GS1-279B7.1	chr1	185292978	185304171	11194	microtubule-associated protein 1 light chain 3 beta pseudogene
LOC10028807	chr1	185292978	185304171	11194	microtubule-associated protein 1 light chain 3 beta pseudogene
AF508906	chr1	185527511	185597620	70110	
LINC01350	chr1	185527511	185597620	70110	long intergenic non-protein coding RNA 1350
AF508907	chr1	185576315	185591914	15600	
HMCN1	chr1	185703682	186160085	456404	hemicentin 1
MIR548F1	chr1	186029866	186446655	416790	microRNA 548f-1
RNU6-72P	chr1	186198963	186281028	82066	RNA, U6 small nuclear 72, pseudogene
PRG4	chr1	186265404	186283688	18285	proteoglycan 4

TPR	chr1	186280785	186344864	64080	translocated promoter region, nuclear basket protein
C1orf27	chr1	186344889	186390503	45615	chromosome 1 open reading frame 27
OCLM	chr1	186369703	186370587	885	oculomedin
PDC	chr1	186412697	186430240	17544	phosducin
LOC10272491Ş	chr1	186404292	186439423	35132	uncharacterized LOC102724919
PTGS2	chr1	186640943	186649559	8617	prostaglandin-endoperoxide synthase 2 (prostaglandin G/H synthase and cyclooxygenase)
PACERR	chr1	186649785	186650578	794	PTGS2 antisense NFKB1 complex-mediated expression regulator RNA
PLA2G4A	chr1	186798031	186958113	160083	phospholipase A2, group IVA (cytosolic, calcium-dependent)
C1orf99	chr1	187610357	187612988	2632	endogenous retrovirus group MER61, member 1
FAM5C	chr1	190066796	190446759	379964	
BRINP3	chr1	190066796	190446759	379964	bone morphogenetic protein/retinoic acid inducible neural-specific 3
CR936711	chr1	190447389	190450524	3136	
LINC01351	chr1	190447389	190450524	3136	long intergenic non-protein coding RNA 1351
LOC440704	chr1	190594019	190770788	176770	uncharacterized LOC440704
RGS18	chr1	192127591	192154945	27355	regulator of G-protein signaling 18
RGS21	chr1	192286121	192336414	50294	regulator of G-protein signaling 21
RGS1	chr1	192544856	192549159	4304	regulator of G-protein signaling 1
RGS13	chr1	192605267	192629440	24174	regulator of G-protein signaling 13
RGS2	chr1	192778168	192781407	3240	regulator of G-protein signaling 2
LINC01032	chr1	192904873	192917387	12515	long intergenic non-protein coding RNA 1032
UCHL5	chr1	192981495	193029237	47743	ubiquitin carboxyl-terminal hydrolase L5
SnoU109	chr1	193026410	193026545	136	
TROVE2	chr1	193028551	193060906	32356	TROVE domain family, member 2
AK022030	chr1	193057300	193060904	3605	
GLRX2	chr1	193065594	193075244	9651	glutaredoxin 2
MIR1278	chr1	193105632	193105713	82	microRNA 1278
B3GALT2	chr1	193147859	193155743	7885	UDP-Gal:betaGlcNAc beta 1,3-galactosyltransferase, polypeptide 2
CDC73	chr1	193091087	193223942	132856	cell division cycle 73
LINC01031	chr1	193273874	193335083	61210	long intergenic non-protein coding RNA 1031
KCNT2	chr1	196194909	196577561	382653	potassium channel, subfamily T, member 2
MIR4735	chr1	196551542	196551611	70	microRNA 4735
CFH	chr1	196621007	196716634	95628	complement factor H
CFHR3	chr1	196743929	196763203	19275	complement factor H-related 3
CFHR4	chr1	196659181	196887843	228663	complement factor H-related 4
CFHR1	chr1	196748291	196801319	53029	complement factor H-related 1
CFHR2	chr1	196912933	196928356	15424	complement factor H-related 2
CFHR5	chr1	196946666	196978803	32138	complement factor H-related 5
F13B	chr1	197008320	197036397	28078	coagulation factor XIII, B polypeptide
ASPM	chr1	197053256	197115824	62569	asp (abnormal spindle) homolog, microcephaly associated (Drosophila)
ZBTB41	chr1	197122813	197169672	46860	zinc finger and BTB domain containing 41
CRB1	chr1	197170591	197447585	276995	crumbs family member 1, photoreceptor morphogenesis associated
DENND1B	chr1	197473878	197744623	270746	DENN/MADD domain containing 1B
C1orf53	chr1	197871681	197876497	4817	chromosome 1 open reading frame 53
LHX9	chr1	197881634	197901715	20082	LIM homeobox 9
NEK7	chr1	198126107	198291548	165442	NIMA-related kinase 7
ATP6V1G3	chr1	198492351	198510075	17725	ATPase, H+ transporting, lysosomal 13kDa, V1 subunit G3
PTPRC	chr1	198608097	198726605	118509	protein tyrosine phosphatase, receptor type, C
MIR181B1	chr1	198828001	198828111	111	microRNA 181b-1
MIR181A1	chr1	198828172	198828282	111	microRNA 181a-1
MIR181A1HG	chr1	198777131	198906558	129428	MIR181A1 host gene (non-protein coding)
LINC01222	chr1	198961717	198988093	26377	long intergenic non-protein coding RNA 1222
BC040869	chr1	198975168	198990166	14999	
NR5A2	chr1	199996729	200146550	149822	nuclear receptor subfamily 5, group A, member 2
LINC00862	chr1	200311671	200342920	31250	long intergenic non-protein coding RNA 862
ZNF281	chr1	200374074	200379186	5113	zinc finger protein 281
EU250746	chr1	200380927	200444641	63715	
KIF14	chr1	200520624	200589862	69239	kinesin family member 14
DDX59	chr1	200610118	200639126	29009	DEAD (Asp-Glu-Ala-Asp) box polypeptide 59
LOC101929224	chr1	200638634	200663378	24745	uncharacterized LOC101929224
CAMSAP2	chr1	200708685	200829835	121151	calmodulin regulated spectrin-associated protein family, member 2
GPR25	chr1	200842082	200843306	1225	G protein-coupled receptor 25
C1orf106	chr1	200860626	200884864	24239	chromosome 1 open reading frame 106
C1orf81	chr1	200898071	200935796	37726	chromosome 1 open reading frame 81
KIF21B	chr1	200938513	200992828	54316	kinesin family member 21B
BC016656	chr1	200993076	200997920	4845	
CACNA1S	chr1	201008639	201081694	73056	calcium channel, voltage-dependent, L type, alpha 1S subunit
LAD1	chr1	201349965	201368669	18705	ladinin 1
TNNI1	chr1	201372894	201398994	26101	troponin I type 1 (skeletal, slow)
PHLDA3	chr1	201434606	201438299	3694	pleckstrin homology-like domain, family A, member 3
MIR5191	chr1	201688635	201688755	121	microRNA 5191
NAV1	chr1	201617449	201796102	178654	neuron navigator 1
IPO9-AS1	chr1	201657383	201798687	141305	IPO9 antisense RNA 1
RNU6-79P	chr1	201736888	201767778	30891	RNA, U6 small nuclear 79, pseudogene
MIR1231	chr1	201777738	201777830	93	microRNA 1231
IPO9	chr1	201798287	201853422	55136	importin 9
MIR6739	chr1	201832500	201832575	76	microRNA 6739
SHISA4	chr1	201857796	201861715	3920	shisa family member 4
LMOD1	chr1	201865583	201915716	50134	leiomodin 1 (smooth muscle)
TIMM17A	chr1	201924618	201939789	15172	translocase of inner mitochondrial membrane 17 homolog A (yeast)
RNPEP	chr1	201951765	201975275	23511	arginyl aminopeptidase (aminopeptidase B)

MIR6740	chr1	201972251	201972364	114	microRNA 6740
ELF3	chr1	201977072	201986315	9244	E74-like factor 3 (ets domain transcription factor, epithelial-specific)
GPR37L1	chr1	202092028	202098634	6607	G-protein coupled receptor 37 like 1
ARL8A	chr1	202102531	202113871	11341	ADP-ribosylation factor-like 8A
PTPN7	chr1	202116140	202130716	14577	protein tyrosine phosphatase, non-receptor type 7
UBE2T	chr1	202300784	202311094	10311	ubiquitin-conjugating enzyme E2T (putative)
PPP1R12B	chr1	202317829	202557697	239869	protein phosphatase 1, regulatory subunit 12B
SYT2	chr1	202559724	202679551	119828	synaptotagmin II
KDM5B	chr1	202696531	202777549	81019	lysine (K)-specific demethylase 5B
PCAT6	chr1	202780073	202781041	969	prostate cancer associated transcript 6 (non-protein coding)
KDM5B-AS1	chr1	202780073	202781041	969	prostate cancer associated transcript 6 (non-protein coding)
MGAT4EP	chr1	202789384	202796341	6958	MGAT4 family, member E, pseudogene
LOC641515	chr1	202789393	202796341	6949	MGAT4 family, member E, pseudogene
BC040684	chr1	202819223	202830736	11514	
BC049825	chr1	202827080	202830736	3657	
LOC148709	chr1	202830881	202844369	13489	actin pseudogene
RABIF	chr1	202847409	202858385	10977	RAB interacting factor
KLHL12	chr1	202860229	202897727	37499	kelch-like family member 12
ADIPOR1	chr1	202909952	202927700	17749	adiponectin receptor 1
PPFIA4	chr1	203003611	203047864	44254	protein tyrosine phosphatase, receptor type, f polypeptide (PTPRF), interacting protein (liprin), alpha 4
MYOG	chr1	203052256	203055166	2911	myogenin (myogenic factor 4)
ADORA1	chr1	203096835	203136533	39699	adenosine A1 receptor
MYBPH	chr1	203136938	203144942	8005	myosin binding protein H
BC034684	chr1	203148063	203148611	549	
CHI3L1	chr1	203148058	203155922	7865	chitinase 3-like 1 (cartilage glycoprotein-39)
CHIT1	chr1	203181958	203241937	59980	chitinase 1 (chitotriosidase)
LINC01353	chr1	203256279	203257924	1646	long intergenic non-protein coding RNA 1353
LINC01136	chr1	203267885	203274453	6569	long intergenic non-protein coding RNA 1136
LOC730227	chr1	203267885	203274453	6569	long intergenic non-protein coding RNA 1136
BTG2	chr1	203274663	203278729	4067	BTG family, member 2
FMOD	chr1	203309748	203320557	10810	fibromodulin
ATP2B4	chr1	203595914	203713209	117296	ATPase, Ca++ transporting, plasma membrane 4
U42379	chr1	203696679	203696730	52	
SNORA77	chr1	203698708	203698833	126	small nucleolar RNA, H/ACA box 77
LINC00260	chr1	203699704	203700979	1276	long intergenic non-protein coding RNA 260
LAX1	chr1	203734283	203745480	11198	lymphocyte transmembrane adaptor 1
ZBED6	chr1	203766650	203769590	2941	zinc finger, BED-type containing 6
ZC3H11A	chr1	203764750	203823256	58507	zinc finger CCCH-type containing 11A
SNRPE	chr1	203830726	203840280	9555	small nuclear ribonucleoprotein polypeptide E
LINC00303	chr1	204001574	204010392	8819	long intergenic non-protein coding RNA 303
SOX13	chr1	204042245	204096871	54627	SRY (sex determining region Y)-box 13
ETNK2	chr1	204100188	204121310	21123	ethanolamine kinase 2
PLEKHA6	chr1	204218311	204226997	8687	pleckstrin homology domain containing, family A member 6
PLEKHA6	chr1	204187978	204329057	141080	pleckstrin homology domain containing, family A member 6
PIK3C2B	chr1	204391757	204463852	72096	phosphatidylinositol-4-phosphate 3-kinase, catalytic subunit type 2 beta
TRNA_Lys	chr1	204475654	204475727	74	
MDM4	chr1	204485506	204677661	192156	MDM4, p53 regulator
AK097184	chr1	204595902	204598840	2939	
LRRN2	chr1	204586302	204654597	68296	leucine rich repeat neuronal 2
NFASC	chr1	204797781	204991950	194170	neurofascin
AX746920	chr1	204989426	204991950	2525	
RBBP5	chr1	205055269	205091150	35882	retinoblastoma binding protein 5
DSTYK	chr1	205111630	205180727	69098	dual serine/threonine and tyrosine protein kinase
TMCC2	chr1	205197037	205242471	45435	transmembrane and coiled-coil domain family 2
NUAK2	chr1	205271190	205290883	19694	NUAK family, SNF1-like kinase, 2
KLHDC8A	chr1	205305192	205326218	21027	kelch domain containing 8A
LEMD1-AS1	chr1	205342379	205356568	14190	LEMD1 antisense RNA 1
LEMD1	chr1	205350505	205391214	40710	LEM domain containing 1
BLACAT1	chr1	205404013	205425214	21202	bladder cancer associated transcript 1 (non-protein coding)
MIR135B	chr1	205417429	205417526	98	microRNA 135b
AK095633	chr1	205425185	205438152	12968	
CDK18	chr1	205473683	205501921	28239	cyclin-dependent kinase 18
LOC284578	chr1	205523400	205525763	2364	uncharacterized LOC284578
MFSD4	chr1	205538111	205572046	33936	major facilitator superfamily domain containing 4
ELK4	chr1	205566694	205649630	82937	ELK4, ETS-domain protein (SRF accessory protein 1)
SLC45A3	chr1	205592803	205649630	56828	solute carrier family 45, member 3
NUCKS1	chr1	205681946	205719372	37427	nuclear casein kinase and cyclin-dependent kinase substrate 1
RAB7L1	chr1	205737113	205744610	7498	
RAB29	chr1	205737113	205744610	7498	RAB29, member RAS oncogene family
IL10	chr1	206940947	206946034	5088	interleukin 10
IL19	chr1	206972214	207016326	44113	interleukin 19
C1orf116	chr1	207191865	207206101	14237	chromosome 1 open reading frame 116
YOD1	chr1	207217193	207226325	9133	YOD1 deubiquitinase
PFKFB2	chr1	207207760	207254368	46609	6-phosphofructo-2-kinase/fructose-2,6-biphosphatase 2
C4BPB	chr1	207262211	207273337	11127	complement component 4 binding protein, beta
AX229788	chr1	207277510	207277617	108	
C4BPA	chr1	207277606	207318317	40712	complement component 4 binding protein, alpha
CD55	chr1	207494816	207534311	39496	CD55 molecule, decay accelerating factor for complement (Cromer blood group)
CR2	chr1	207627644	207663240	35597	complement component (3d/Epstein Barr virus) receptor 2
CR1	chr1	207669472	207815110	145639	complement component (3b/4b) receptor 1 (Knops blood group)

CR1L	chr1	207818457	207898053	79597	complement component (3b/4b) receptor 1-like
CD46	chr1	207925382	207968861	43480	CD46 molecule, complement regulatory protein
MIR29C	chr1	207975196	207975868	673	microRNA 29c
MIR29B2	chr1	207975787	207975868	82	microRNA 29b-2
mir-29b-2	chr1	207974866	207996048	21183	
LOC148696	chr1	207991723	207995941	4219	uncharacterized LOC148696
AK123177	chr1	208039465	208042417	2953	
CD34	chr1	208059882	208084683	24802	CD34 molecule
PLXNA2	chr1	208195587	208417665	222079	plexin A2
MIR205HG	chr1	209602167	209605892	3726	MIR205 host gene (non-protein coding)
MIR205	chr1	209605477	209605587	111	microRNA 205
CAMK1G	chr1	209757044	209787284	30241	calcium/calmodulin-dependent protein kinase IG
MIR4260	chr1	209796788	209796855	68	microRNA 4260
LAMB3	chr1	209788217	209825820	37604	laminin, beta 3
G0S2	chr1	209848669	209849735	1067	G0/G1 switch 2
HSD11B1	chr1	209859524	209908295	48772	hydroxysteroid (11-beta) dehydrogenase 1
DIEXF	chr1	210001311	210030910	29600	digestive organ expansion factor homolog (zebrafish)
SYT14	chr1	210111518	210337633	226116	synaptotagmin XIV
SERTAD4-AS1	chr1	210404803	210407466	2664	SERTAD4 antisense RNA 1
SERTAD4	chr1	210406194	210416440	10247	SERTA domain containing 4
HHAT	chr1	210501595	210849638	348044	hedgehog acyltransferase
KCNH1	chr1	210851656	211307457	455802	potassium voltage-gated channel, subfamily H (eag-related), member 1
RCOR3	chr1	211432707	211489725	57019	REST corepressor 3
TRAF5	chr1	211499956	211548286	48331	TNF receptor-associated factor 5
LINC00467	chr1	211556096	211605877	49782	long intergenic non-protein coding RNA 467
RD3	chr1	211649863	211666259	16397	retinal degeneration 3
SLC30A1	chr1	211748380	211752099	3720	solute carrier family 30 (zinc transporter), member 1
NEK2	chr1	211831598	211848972	17375	NIMA-related kinase 2
LPGAT1	chr1	211916798	212004114	87317	lysophosphatidylglycerol acyltransferase 1
INTS7	chr1	212113740	212209002	95263	integrator complex subunit 7
DTL	chr1	212208894	212278348	69455	denticleless E3 ubiquitin protein ligase homolog (Drosophila)
MIR3122	chr1	212250954	212251027	74	microRNA 3122
BC010168	chr1	212317864	212318301	438	
LOC101929541	chr1	212386343	212458363	72021	uncharacterized LOC101929541
PPP2R5A	chr1	212458878	212535205	76328	protein phosphatase 2, regulatory subunit B', alpha
SNORA16B	chr1	212526159	212526292	134	small nucleolar RNA, H/ACA box 16B
TMEM206	chr1	212537815	212588267	50453	transmembrane protein 206
NENF	chr1	212606228	212619721	13494	neudesin neurotrophic factor
LOC101929565	chr1	212719035	212729408	10374	uncharacterized LOC101929565
ATF3	chr1	212738675	212794119	55445	activating transcription factor 3
FAM71A	chr1	212797625	212800120	2496	family with sequence similarity 71, member A
BATF3	chr1	212859758	212873327	13570	basic leucine zipper transcription factor, ATF-like 3
NSL1	chr1	212899494	212965139	65646	NSL1, MIS12 kinetochore complex component
TATDN3	chr1	212965169	212990167	24999	TatD DNase domain containing 3
SPATA45	chr1	213003484	213020991	17508	spermatogenesis associated 45
C1orf227	chr1	213003484	213020991	17508	spermatogenesis associated 45
FLVCR1-AS1	chr1	213029945	213031480	1536	FLVCR1 antisense RNA 1 (head to head)
FLVCR1	chr1	213031596	213072705	41110	feline leukemia virus subgroup C cellular receptor 1
VASH2	chr1	213123861	213164927	41067	vasohibin 2
ANGEL2	chr1	213165523	213189217	23695	angel homolog 2 (Drosophila)
RPS6KC1	chr1	213224574	213446808	222235	ribosomal protein S6 kinase, 52kDa, polypeptide 1
AK092251	chr1	213992977	214139607	146631	
LINC00538	chr1	214098091	214099996	1906	long intergenic non-protein coding RNA 538
PROX1	chr1	214161277	214214847	53571	prospero homeobox 1
SMYD2	chr1	214454564	214510477	55914	SET and MYND domain containing 2
PTPN14	chr1	214522038	214725024	202987	protein tyrosine phosphatase, non-receptor type 14
BC171896	chr1	214655881	214656819	939	
CENPF	chr1	214776531	214837914	61384	centromere protein F, 350/400kDa
KCNK2	chr1	215178884	215410436	231553	potassium channel, subfamily K, member 2
KCTD3	chr1	215740734	215795149	54416	potassium channel tetramerization domain containing 3
USH2A	chr1	215796235	216596738	800504	Usher syndrome 2A (autosomal recessive, mild)
LOC102723833	chr1	216245806	216260259	14454	uncharacterized LOC102723833
Mir_598	chr1	216825011	216825068	58	
ESRRG	chr1	216676587	217311097	634511	estrogen-related receptor gamma
GPATCH2	chr1	217600334	217804444	204111	G patch domain containing 2
SPATA17	chr1	217804694	218040484	235791	spermatogenesis associated 17
SPATA17-AS1	chr1	217954539	217958462	3924	SPATA17 antisense RNA 1
LOC101929631	chr1	218216846	218232482	15637	uncharacterized LOC101929631
RRP15	chr1	218458628	218511325	52698	ribosomal RNA processing 15 homolog (S. cerevisiae)
LOC728463	chr1	218517537	218519020	1484	TGFB2 antisense RNA 1 (head to head)
TGFB2-AS1	chr1	218517537	218519020	1484	TGFB2 antisense RNA 1 (head to head)
TGFB2	chr1	218518675	218617961	99287	transforming growth factor, beta 2
TGFB2-OT1	chr1	218615967	218617337	1371	TGFB2 overlapping transcript 1
LYPLAL1-AS1	chr1	219254316	219347130	92815	LYPLAL1 antisense RNA 1 (head to head)
LOC643723	chr1	219254316	219347130	92815	LYPLAL1 antisense RNA 1 (head to head)
LYPLAL1	chr1	219347172	219386207	39036	lysophospholipase-like 1
SLC30A10	chr1	219858768	220131989	273222	solute carrier family 30, member 10
RNU5F-1	chr1	220046618	220292777	246160	RNA, U5F small nuclear 1
EPRS	chr1	220141941	220220000	78060	glutamyl-prolyl-tRNA synthetase
BPNT1	chr1	220230823	220263195	32373	3'(2'), 5'-bisphosphate nucleotidase 1

MIR215	chr1	220291194	220291304	111	microRNA 215
MIR194-1	chr1	220291498	220291583	86	microRNA 194-1
DM119532	chr1	220291547	220291569	23	
IARS2	chr1	220267454	220321383	53930	isoleucyl-tRNA synthetase 2, mitochondrial
MIR664A	chr1	220373883	220373961	79	microRNA 664a
SNORA36B	chr1	220373887	220374018	132	small nucleolar RNA, H/ACA box 36B
RAB3GAP2	chr1	220321609	220445843	124235	RAB3 GTPase activating protein subunit 2 (non-catalytic)
AURKAPS1	chr1	220439520	220441057	1538	aurora kinase A pseudogene 1
MARK1	chr1	220701524	220837799	136276	MAP/microtubule affinity-regulating kinase 1
DUSP10	chr1	221874761	221915518	40758	dual specificity phosphatase 10
LOC101929771	chr1	222001007	222014008	13002	uncharacterized LOC101929771
TRNA_Thr	chr1	222638346	222638419	74	
DQ587965	chr1	222646009	222646043	35	
DQ573170	chr1	222646722	222646767	46	
DQ576410	chr1	222646839	222646870	32	
DQ584993	chr1	222647751	222647786	36	
DQ584971	chr1	222648391	222648423	33	
DQ575983	chr1	222648536	222648596	61	
DQ597892	chr1	222648680	222648717	38	
DQ574659	chr1	222649002	222649064	63	
DQ584609	chr1	222649161	222649251	91	
DQ600234	chr1	222650319	222650381	63	
HHIPL2	chr1	222695601	222721444	25844	HHIP-like 2
TAF1A	chr1	222731243	222763275	32033	TATA box binding protein (TBP)-associated factor, RNA polymerase I, A, 48kDa
TAF1A-AS1	chr1	222763261	222765975	2715	TAF1A antisense RNA 1
MIA3	chr1	222791443	222841354	49912	melanoma inhibitory activity family, member 3
AIDA	chr1	222841354	222886526	45173	axin interactor, dorsalization associated
BROX	chr1	222885894	222908538	22645	BRO1 domain and CAAX motif containing
AK025140	chr1	222906202	222908533	2332	
FAM177B	chr1	222910557	222924002	13446	family with sequence similarity 177, member B
AK094916	chr1	222900275	222946483	46209	
DISP1	chr1	222988430	223179337	190908	dispatched homolog 1 (Drosophila)
TLR5	chr1	223282747	223316624	33878	toll-like receptor 5
SUSD4	chr1	223394160	223537544	143385	sushi domain containing 4
C1orf65	chr1	223566714	223568812	2099	coiled-coil domain containing 185
CCDC185	chr1	223566714	223568812	2099	coiled-coil domain containing 185
CAPN8	chr1	223714971	223853436	138466	calpain 8
CAPN2	chr1	223889294	223963720	74427	calpain 2, (m/II) large subunit
TP53BP2	chr1	223967594	224033674	66081	tumor protein p53 binding protein 2
HM358976	chr1	224051997	224052306	310	
AK124970	chr1	224189608	224197818	8211	
FBXO28	chr1	224301788	224349749	47962	F-box protein 28
DEGS1	chr1	224370909	224381142	10234	delta(4)-desaturase, sphingolipid 1
LOC101927143	chr1	224396449	224400981	4533	uncharacterized LOC101927143
LOC101927164	chr1	224407314	224415745	8432	uncharacterized LOC101927164
MIR320B2	chr1	224444705	224444843	139	microRNA 320b-2
NVL	chr1	224415035	224517891	102857	nuclear VCP-like
CNIH4	chr1	224544512	224567153	22642	cornichon family AMPA receptor auxiliary protein 4
MIR4742	chr1	224585928	224586013	86	microRNA 4742
DL490795	chr1	224586541	224586601	61	
WDR26	chr1	224572844	224622001	49158	WD repeat domain 26
AB586698	chr1	224605809	224606083	275	
CNIH3	chr1	224804178	224928249	124072	cornichon family AMPA receptor auxiliary protein 3
DNAH14	chr1	225117355	225586996	469642	dynein, axonemal, heavy chain 14
LBR	chr1	225589203	225616557	27355	lamin B receptor
ENAH	chr1	225674533	225840845	166313	enabled homolog (Drosophila)
AK124056	chr1	225888305	225939945	51641	
SRP9	chr1	225965514	225978168	12655	signal recognition particle 9kDa
EPHX1	chr1	225997775	226033262	35488	epoxide hydrolase 1, microsomal (xenobiotic)
LEFTY1	chr1	226073981	226112040	38060	left-right determination factor 1
PYCR2	chr1	226107576	226112040	4465	pyrroline-5-carboxylate reductase family, member 2
MIR6741	chr1	226109779	226109842	64	microRNA 6741
LEFTY2	chr1	226124297	226129083	4787	left-right determination factor 2
SDE2	chr1	226170402	226187066	16665	SDE2 telomere maintenance homolog (S. pombe)
H3F3A	chr1	226250407	226259703	9297	H3 histone, family 3A
H3F3AP4	chr1	226250427	226259703	9277	H3 histone, family 3A, pseudogene 4
BC032899	chr1	226271655	226277994	6340	
ACBD3	chr1	226332379	226374423	42045	acyl-CoA binding domain containing 3
BC033346	chr1	226374532	226385086	10555	
MIXL1	chr1	226411318	226414756	3439	Mix paired-like homeobox
LIN9	chr1	226418849	226497449	78601	lin-9 DREAM MuvB core complex component
PARP1	chr1	226548391	226595801	47411	poly (ADP-ribose) polymerase 1
AK055856	chr1	226723318	226730469	7152	
C1orf95	chr1	226736500	226796915	60416	chromosome 1 open reading frame 95
ITPKB	chr1	226819390	226926876	107487	inositol-trisphosphate 3-kinase B
PSEN2	chr1	227058272	227083804	25533	presenilin 2
CABC1	chr1	227084588	227091365	6778	chaperone, ABC1 activity of bc1 complex like (S. pombe)
ADCK3	chr1	227084588	227175246	90659	aarF domain containing kinase 3
CDC42BPA	chr1	227177565	227505826	328262	CDC42 binding protein kinase alpha (DMPK-like)
DTNB	chr2	25600066	25896516	296451	dystrobrevin, beta

Y_RNA	chr2	25919944	25920057	114	
ASXL2	chr2	25962252	26101312	139061	additional sex combs like transcriptional regulator 2
KIF3C	chr2	26149454	26205443	55990	kinesin family member 3C
GAREML	chr2	26395959	26412532	16574	GRB2 associated, regulator of MAPK1-like
HADHA	chr2	26413503	26467594	54092	hydroxyacyl-CoA dehydrogenase/3-ketoacyl-CoA thiolase/enoyl-CoA hydratase (trifunctional protein), alpha
HADHB	chr2	26467615	26513333	45719	hydroxyacyl-CoA dehydrogenase/3-ketoacyl-CoA thiolase/enoyl-CoA hydratase (trifunctional protein), beta
GPR113	chr2	26531040	26569685	38646	G protein-coupled receptor 113
EMILIN1	chr2	27301434	27309265	7832	elastin microfibril interfacer 1
KHK	chr2	27309610	27323619	14010	ketohexokinase (fructokinase)
CGREF1	chr2	27322220	27341995	19776	cell growth regulator with EF hand domain 1
AK074615	chr2	27346722	27349815	3094	
ABHD1	chr2	27346656	27353680	7025	abhydrolase domain containing 1
PREB	chr2	27353624	27357542	3919	prolactin regulatory element binding
PRR30	chr2	27359714	27362332	2619	proline rich 30
C2orf53	chr2	27359714	27362332	2619	proline rich 30
TCF23	chr2	27371944	27375819	3876	transcription factor 23
SLC5A6	chr2	27422454	27435175	12722	solute carrier family 5 (sodium/multivitamin and iodide cotransporter), member 6
LRP1B	chr2	140988995	142889270	1900276	low density lipoprotein receptor-related protein 1B
NAALADL2	chr3	174156362	174455536	299175	N-acetylated alpha-linked acidic dipeptidase-like 2
LGSN	chr6	63985855	64029882	44028	lengsin, lens protein with glutamine synthetase domain
PTP4A1	chr6	64231650	64293493	61844	protein tyrosine phosphatase type IVA, member 1
PHF3	chr6	64345706	64425418	79713	PHD finger protein 3
EYS	chr6	64429875	66417118	1987244	eyes shut homolog (Drosophila)
LMBRD1	chr6	70385640	70507049	121410	LMBR1 domain containing 1
COL19A1	chr6	70576447	70922157	345711	collagen, type XIX, alpha 1
COL9A1	chr6	70925742	71012786	87045	collagen, type IX, alpha 1
LOC101928353	chr6	71103904	71109120	5217	uncharacterized LOC101928353
FAM135A	chr6	71123106	71270877	147772	family with sequence similarity 135, member A
C6orf57	chr6	71276624	71298606	21983	chromosome 6 open reading frame 57
SMAP1	chr6	71377473	71571716	194244	small ArfGAP 1
B3GAT2	chr6	71571068	71666788	95721	beta-1,3-glucuronyltransferase 2
U3	chr6	71836595	71836808	214	RNA, U3 small nucleolar
OGFRL1	chr6	71998476	72011973	13498	opioid growth factor receptor-like 1
MIR30C2	chr6	72086662	72086734	73	microRNA 30c-2
DM119538	chr6	72113256	72113278	23	
MIR30A	chr6	72113253	72113324	72	microRNA 30a
DM119539	chr6	72113297	72113319	23	
LINC00472	chr6	72117566	72130448	12883	long intergenic non-protein coding RNA 472
LOC102724000	chr6	72130729	72168575	37847	uncharacterized LOC102724000
RIMS1	chr6	72596405	73112845	516441	regulating synaptic membrane exocytosis 1
KCNQ5-IT1	chr6	73340222	73388282	48061	KCNQ5 intronic transcript 1 (non-protein coding)
KCNQ5	chr6	73331570	73908573	577004	potassium voltage-gated channel, KQT-like subfamily, member 5
MIR4282	chr6	73677409	73677476	68	microRNA 4282
KCNQ5-AS1	chr6	73844525	73853237	8713	KCNQ5 antisense RNA 1
KHDC1L	chr6	73933267	73935175	1909	KH homology domain containing 1-like
KHDC1	chr6	73919470	74019938	100469	KH homology domain containing 1
BC031876	chr6	73972949	73986225	13277	
AL832252	chr6	73977773	74001061	23289	
C6orf147	chr6	73983861	74020088	36228	chromosome 6 open reading frame 147
DPPA5	chr6	74062784	74063999	1216	developmental pluripotency associated 5
KHDC3L	chr6	74072399	74073898	1500	KH domain containing 3-like, subcortical maternal complex member
OOEP	chr6	74078279	74104816	26538	oocyte expressed protein
DDX43	chr6	74104284	74127289	23006	DEAD (Asp-Glu-Ala-Asp) box polypeptide 43
MB21D1	chr6	74134855	74162043	27189	Mab-21 domain containing 1
MTO1	chr6	74189658	74190528	871	mitochondrial tRNA translation optimization 1
MTO1	chr6	74171453	74211179	39727	mitochondrial tRNA translation optimization 1
EEF1A1	chr6	74225472	74230755	5284	eukaryotic translation elongation factor 1 alpha 1
DL491467	chr6	74228125	74228181	57	
DQ590492	chr6	74228271	74228299	29	
SLC17A5	chr6	74303101	74363737	60637	solute carrier family 17 (acidic sugar transporter), member 5
AK124950	chr6	74403625	74405743	2119	
LOC101928489	chr6	74403625	74405854	2230	uncharacterized LOC101928489
CD109	chr6	74405807	74538041	132235	CD109 molecule
LOC101928516	chr6	74779166	75400443	621278	uncharacterized LOC101928516
AF086303	chr6	74779166	75400443	621278	
COL12A1	chr6	75794041	75915623	121583	collagen, type XII, alpha 1
COX7A2	chr6	75947390	75953644	6255	cytochrome c oxidase subunit VIIa polypeptide 2 (liver)
TMEM30A	chr6	75962637	75994632	31996	transmembrane protein 30A
LOC100506804	chr6	75994729	76001580	6852	uncharacterized LOC100506804
FILIP1	chr6	76004606	76203545	198940	filamin A interacting protein 1
LOC101928540	chr6	76092901	76168615	75715	uncharacterized LOC101928540
SENP6	chr6	76311621	76427994	116374	SUMO1/sentrin specific peptidase 6
MYO6	chr6	76458892	76629254	170363	myosin VI
IMPG1	chr6	76630830	76782395	151566	interphotoreceptor matrix proteoglycan 1
HTR1B	chr6	78171947	78173120	1174	5-hydroxytryptamine (serotonin) receptor 1B, G protein-coupled
MEI4	chr6	78400372	78634476	234105	meiosis-specific 4 homolog (S. cerevisiae)
IRAK1BP1	chr6	79577260	79656157	78898	interleukin-1 receptor-associated kinase 1 binding protein 1
PHIP	chr6	79644135	79788011	143877	pleckstrin homology domain interacting protein
HMGN3	chr6	79910961	79944455	33495	high mobility group nucleosomal binding domain 3
HMGN3-AS1	chr6	79943390	79946517	3128	HMGN3 antisense RNA 1

LOC100288198	chr6	79943390	79946517	3128	HMGN3 antisense RNA 1
BC070061	chr6	80017385	80023101	5717	
LCA5	chr6	80194707	80247147	52441	Leber congenital amaurosis 5
DQ582269	chr6	80248450	80248479	30	
SH3BGRL2	chr6	80340999	80413369	72371	SH3 domain binding glutamate-rich protein like 2
RNY4	chr6	80451635	80451669	35	RNA, Ro-associated Y4
C6orf7	chr6	80513303	80580137	66835	chromosome 6 open reading frame 7
ELOVL4	chr6	80624528	80657315	32788	ELOVL fatty acid elongase 4
TTK	chr6	80714321	80752244	37924	TTK protein kinase
BCKDHB	chr6	80816343	81055987	239645	branched chain keto acid dehydrogenase E1, beta polypeptide
FAM46A	chr6	82455446	82462428	6983	family with sequence similarity 46, member A
BC038576	chr6	82523002	82523909	908	
IBTK	chr6	82879703	82957471	77769	inhibitor of Bruton agammaglobulinemia tyrosine kinase
TPBG	chr6	83072922	83077133	4212	trophoblast glycoprotein
UBE3D	chr6	83602185	83775545	173361	ubiquitin protein ligase E3D
DOPEY1	chr6	83777384	83881065	103682	dopey family member 1
PGM3	chr6	83874592	83903655	29064	phosphoglucomutase 3
RWDD2A	chr6	83903031	83906256	3226	RWD domain containing 2A
ME1	chr6	83920109	84140938	220830	malic enzyme 1, NADP(+)-dependent, cytosolic
PRSS35	chr6	84222193	84235421	13229	protease, serine, 35
SNAP91	chr6	84262604	84419127	156524	synaptosomal-associated protein, 91kDa
RIPPLY2	chr6	84562984	84567234	4251	ripply transcriptional repressor 2
CYB5R4	chr6	84569369	84670146	100778	cytochrome b5 reductase 4
MRAP2	chr6	84743419	84800605	57187	melanocortin 2 receptor accessory protein 2
KIAA1009	chr6	84833959	84937335	103377	
CEP162	chr6	84833959	84937353	103395	centrosomal protein 162kDa
LOC102724201	chr6	85399175	85419252	20078	uncharacterized LOC102724201
TBX18	chr6	85397078	85473954	76877	T-box 18
AK024998	chr6	86096936	86099904	2969	
NT5E	chr6	86159301	86205509	46209	5'-nucleotidase, ecto (CD73)
SNX14	chr6	86215214	86303850	88637	sorting nexin 14
SYNCRIP	chr6	86317501	86353043	35543	synaptotagmin binding, cytoplasmic RNA interacting protein
SNORD50A	chr6	86387011	86387086	76	small nucleolar RNA, C/D box 50A
SNORD50B	chr6	86387306	86387377	72	small nucleolar RNA, C/D box 50B
SNHG5	chr6	86386724	86388451	1728	small nucleolar RNA host gene 5 (non-protein coding)
HTR1E	chr6	87647023	87726397	79375	5-hydroxytryptamine (serotonin) receptor 1E, G protein-coupled
CGA	chr6	87795215	87804865	9651	glycoprotein hormones, alpha polypeptide
ZNF292	chr6	87862550	87973406	110857	zinc finger protein 292
GJB7	chr6	87992696	88038996	46301	gap junction protein, beta 7, 25kDa
SMIM8	chr6	88032305	88085428	53124	small integral membrane protein 8
C6orf163	chr6	88054570	88075181	20612	chromosome 6 open reading frame 163
C6orf164	chr6	88091719	88109459	17741	chromosome 6 open reading frame 164
C6orf165	chr6	88117689	88205858	88170	chromosome 6 open reading frame 165
SLC35A1	chr6	88146443	88222057	75615	solute carrier family 35 (CMP-sialic acid transporter), member A1
AK299432	chr6	88218259	88218731	473	
RARS2	chr6	88224095	88299735	75641	arginyl-tRNA synthetase 2, mitochondrial
ORC3	chr6	88299784	88377172	77389	origin recognition complex, subunit 3
AKIRIN2	chr6	88384577	88411985	27409	akirin 2
LOC101928911	chr6	88494577	88621449	126873	uncharacterized LOC101928911
AY927641	chr6	88494577	88621449	126873	
SPACA1	chr6	88757506	88776550	19045	sperm acrosome associated 1
CNR1	chr6	88849584	88875767	26184	cannabinoid receptor 1 (brain)
LOC101928936	chr6	89235642	89249088	13447	uncharacterized LOC101928936
JQ733905	chr6	89372191	89380624	8434	
RNGTT	chr6	89319615	89673348	353734	RNA guanylyltransferase and 5'-phosphatase
PNRC1	chr6	89790428	89794879	4452	proline-rich nuclear receptor coactivator 1
SRSF12	chr6	89805677	89827800	22124	serine/arginine-rich splicing factor 12
PM20D2	chr6	89855768	89875288	19521	peptidase M20 domain containing 2
GABRR1	chr6	89887222	89941007	53786	gamma-aminobutyric acid (GABA) A receptor, rho 1
GABRR2	chr6	89966839	90025018	58180	gamma-aminobutyric acid (GABA) A receptor, rho 2
UBE2J1	chr6	90036343	90062619	26277	ubiquitin-conjugating enzyme E2, J1
RRAGD	chr6	90074334	90121995	47662	Ras-related GTP binding D
ANKRD6	chr6	90142896	90343553	200658	ankyrin repeat domain 6
LYRM2	chr6	90277862	90348474	70613	LYR motif containing 2
LOC101929057	chr6	90348250	90399200	50951	uncharacterized LOC101929057
MDN1	chr6	90352493	90529513	177021	MDN1, midasin homolog (yeast)
CASP8AP2	chr6	90539618	90584155	44538	caspase 8 associated protein 2
GJA10	chr6	90604187	90605819	1633	gap junction protein, alpha 10, 62kDa
BACH2	chr6	90636246	91006627	370382	BTB and CNC homology 1, basic leucine zipper transcription factor 2
MIR4464	chr6	91022460	91022552	93	microRNA 4464
Mir_562	chr6	91177261	91177362	102	
MAP3K7	chr6	91223291	91297020	73730	mitogen-activated protein kinase kinase kinase 7
MIR4643	chr6	92231377	92231455	79	microRNA 4643
BC037927	chr6	92338753	92400146	61394	
CASC6	chr6	92338757	92400146	61390	cancer susceptibility candidate 6 (non-protein coding)
EPHA7	chr6	93949739	94129300	179562	EPH receptor A7
TSG1	chr6	94416800	94486199	69400	tumor suppressor TSG1
MANEA-AS1	chr6	96007971	96025327	17357	MANEA antisense RNA 1 (head to head)
MANEA	chr6	96025372	96057328	31957	mannosidase, endo-alpha
FUT9	chr6	96463844	96663488	199645	fucosyltransferase 9 (alpha (1,3) fucosyltransferase)

UFL1	chr6	96969701	97003151	33451	UFM1-specific ligase 1
FHL5	chr6	97010423	97064512	54090	four and a half LIM domains 5
U4	chr6	97097328	97097467	140	RNA, U4A small nuclear
GPR63	chr6	97241997	97285353	43357	G protein-coupled receptor 63
NDUFAF4	chr6	97337186	97345767	8582	NADH dehydrogenase (ubiquinone) complex I, assembly factor 4
Metazoa_SRP	chr6	97362643	97362871	229	
KLHL32	chr6	97372495	97588630	216136	kelch-like family member 32
MMS22L	chr6	97590036	97731061	141026	MMS22-like, DNA repair protein
MIR548H3	chr6	97537842	97862283	324442	microRNA 548h-3
AK091365	chr6	97753512	98156726	403215	
LOC101927314	chr6	97753461	98156793	403333	uncharacterized LOC101927314
MIR2113	chr6	98472406	98472495	90	microRNA 2113
POU3F2	chr6	99282579	99286666	4088	POU class 3 homeobox 2
FBXL4	chr6	99321600	99395882	74283	F-box and leucine-rich repeat protein 4
FAXC	chr6	99720792	99797531	76740	failed axon connections homolog (Drosophila)
COQ3	chr6	99817347	99842082	24736	coenzyme Q3 methyltransferase
BC033061	chr6	99846536	99848504	1969	
PNISR	chr6	99846536	99873207	26672	PNN-interacting serine/arginine-rich protein
DQ599242	chr6	99872797	99875596	2800	
LOC101927365	chr6	99872746	99879190	6445	uncharacterized LOC101927365
USP45	chr6	99880183	99963308	83126	ubiquitin specific peptidase 45
TSTD3	chr6	99968869	100033084	64216	thiosulfate sulfurtransferase (rhodanese)-like domain containing 3
CCNC	chr6	99990262	100016690	26429	cyclin C
PRDM13	chr6	100054649	100063454	8806	PR domain containing 13
MCHR2	chr6	100367785	100442114	74330	melanin-concentrating hormone receptor 2
MCHR2-AS1	chr6	100441913	100524295	82383	MCHR2 antisense RNA 1
LOC728012	chr6	100441913	100524295	82383	MCHR2 antisense RNA 1
SIM1	chr6	100836749	100912805	76057	single-minded family bHLH transcription factor 1
ASCC3	chr6	100956070	101329248	373179	activating signal cointegrator 1 complex subunit 3
DQ585302	chr6	101878708	101878736	29	
GRIK2	chr6	101841381	102517958	676578	glutamate receptor, ionotropic, kainate 2
HACE1	chr6	105175967	105307794	131828	HECT domain and ankyrin repeat containing E3 ubiquitin protein ligase 1
LINC00577	chr6	105384168	105388402	4235	long intergenic non-protein coding RNA 577
LIN28B	chr6	105404922	105531207	126286	lin-28 homolog B (C. elegans)
BVES	chr6	105544698	105585049	40352	blood vessel epicardial substance
BVES-AS1	chr6	105585561	105617819	32259	BVES antisense RNA 1
POPDC3	chr6	105605774	105627858	22085	popeye domain containing 3
PREP	chr6	105725441	105850999	125559	prolyl endopeptidase
PRDM1	chr6	106534194	106557814	23621	PR domain containing 1, with ZNF domain
ATG5	chr6	106632351	106773695	141345	autophagy related 5
AIM1	chr6	106959729	107018334	58606	absent in melanoma 1
RTN4IP1	chr6	107018902	107078366	59465	reticulon 4 interacting protein 1
QRSL1	chr6	107077440	107116292	38853	glutaminyl-tRNA synthase (glutamine-hydrolyzing)-like 1
LOC100422737	chr6	107165326	107235300	69975	uncharacterized LOC100422737
AK123416	chr6	107218004	107222877	4874	
C6orf203	chr6	107349375	107372790	23416	chromosome 6 open reading frame 203
BEND3	chr6	107386384	107435636	49253	BEN domain containing 3
PDSS2	chr6	107473760	107780779	307020	prenyl (decaprenyl) diphosphate synthase, subunit 2
SOBP	chr6	107811316	107982513	171198	sine oculis binding protein homolog (Drosophila)
SCML4	chr6	108023360	108145521	122162	sex comb on midleg-like 4 (Drosophila)
SEC63	chr6	108188959	108279482	90524	SEC63 homolog (S. cerevisiae)
AJ420489	chr6	108362675	108365439	2765	
OSTM1	chr6	108362612	108395941	33330	osteopetrosis associated transmembrane protein 1
AF520419	chr6	108444839	108479222	34384	
NR2E1	chr6	108487261	108510013	22753	nuclear receptor subfamily 2, group E, member 1
SNX3	chr6	108532420	108582464	50045	sorting nexin 3
LACE1	chr6	108616097	108844251	228155	lactation elevated 1
FOXO3	chr6	108881025	109005971	124947	forkhead box O3
LINC00222	chr6	109072856	109091145	18290	long intergenic non-protein coding RNA 222
ARMC2	chr6	109169618	109295675	126058	armadillo repeat containing 2
ARMC2-AS1	chr6	109229342	109245306	15965	ARMC2 antisense RNA 1
SESN1	chr6	109307639	109415708	108070	sestrin 1
CEP57L1	chr6	109416355	109485115	68761	centrosomal protein 57kDa-like 1
LOC100996634	chr6	109557816	109591717	33902	transmembrane protein FLJ37396
CCDC162P	chr6	109615505	109629423	13919	coiled-coil domain containing 162, pseudogene
C6orf185	chr6	109659677	109676151	16475	coiled-coil domain containing 162, pseudogene
CD164	chr6	109687716	109703762	16047	CD164 molecule, sialomucin
PPIL6	chr6	109711417	109762374	50958	peptidylprolyl isomerase (cyclophilin)-like 6
SMPD2	chr6	109761930	109765122	3193	sphingomyelin phosphodiesterase 2, neutral membrane (neutral sphingomyelinase)
MICAL1	chr6	109765265	109787171	21907	microtubule associated monooxygenase, calponin and LIM domain containing 1
ZBTB24	chr6	109783718	109804440	20723	zinc finger and BTB domain containing 24
AK9	chr6	109850073	109871740	21668	adenylate kinase 9
AK9	chr6	109814058	110012415	198358	adenylate kinase 9
FIG4	chr6	110012423	110146634	134212	FIG4 phosphoinositide 5-phosphatase
GPR6	chr6	110299458	110301924	2467	G protein-coupled receptor 6
WASF1	chr6	110421021	110501207	80187	WAS protein family, member 1
CDC40	chr6	110501623	110553422	51800	cell division cycle 40
METTL24	chr6	110567148	110731939	164792	methyltransferase like 24
DDO	chr6	110713382	110736753	23372	D-aspartate oxidase
SLC22A16	chr6	110745891	110797844	51954	solute carrier family 22 (organic cation/carnitine transporter), member 16

CDK19	chr6	110931180	111137088	205909	cyclin-dependent kinase 19
BC047513	chr6	111133327	111136191	2865	
AMD1	chr6	111135823	111216915	81093	adenosylmethionine decarboxylase 1
GTF3C6	chr6	111279762	111289091	9330	general transcription factor IIIC, polypeptide 6, alpha 35kDa
RPF2	chr6	111303219	111349466	46248	ribosome production factor 2 homolog (S. cerevisiae)
GSTM2P1	chr6	111367621	111368757	1137	glutathione S-transferase mu 2 (muscle) pseudogene 1
SLC16A10	chr6	111408780	111544606	135827	solute carrier family 16 (aromatic amino acid transporter), member 10
KIAA1919	chr6	111580481	111590261	9781	KIAA1919
REV3L	chr6	111620233	111804918	184686	REV3-like, polymerase (DNA directed), zeta, catalytic subunit
AF216583	chr6	111816121	111816969	849	
TRAF3IP2-AS1	chr6	111804674	111923497	118824	TRAF3IP2 antisense RNA 1
TRAF3IP2	chr6	111876580	111927477	50898	TRAF3 interacting protein 2
FYN	chr6	111981534	112194655	213122	FYN proto-oncogene, Src family tyrosine kinase
WISP3	chr6	112375277	112390887	15611	WNT1 inducible signaling pathway protein 3
TUBE1	chr6	112391859	112408751	16893	tubulin, epsilon 1
FAM229B	chr6	112408673	112423993	15321	family with sequence similarity 229, member B
LAMA4	chr6	112429133	112575917	146785	laminin, alpha 4
LOC10192764C	chr6	112557796	112627885	70090	uncharacterized LOC101927640
RFPL4B	chr6	112668531	112672498	3968	ret finger protein-like 4B
LOC10192768€	chr6	113944736	113971277	26542	uncharacterized LOC101927686
MARCKS	chr6	114178526	114184652	6127	myristoylated alanine-rich protein kinase C substrate
LINC01268	chr6	114189178	114194512	5335	long intergenic non-protein coding RNA 1268
LOC285758	chr6	114189178	114194512	5335	long intergenic non-protein coding RNA 1268
FLJ34503	chr6	114225550	114242806	17257	uncharacterized FLJ34503
HDAC2	chr6	114257319	114292359	35041	histone deacetylase 2
LOC10192776E	chr6	114290864	114661893	371030	uncharacterized LOC101927768
BC042098	chr6	114290864	114661899	371036	
Mir_320	chr6	114499969	114500051	83	
HS3ST5	chr6	114376749	114663540	286792	heparan sulfate (glucosamine) 3-O-sulfotransferase 5
Mir_548	chr6	114598550	114598634	85	
Mir_584	chr6	114743162	114743280	119	
FRK	chr6	116262692	116381921	119230	fyn-related Src family tyrosine kinase
TPI1P3	chr6	116359893	116361107	1215	triosephosphate isomerase 1 pseudogene 3
COL10A1	chr6	116440084	116447296	7213	collagen, type X, alpha 1
NT5DC1	chr6	116421998	116566853	144856	5'-nucleotidase domain containing 1
TSPYL4	chr6	116571130	116575261	4132	TSPY-like 4
AK093256	chr6	116575436	116577906	2471	
TSPYL1	chr6	116596021	116601280	5260	TSPY-like 1
DSE	chr6	116575335	116759442	184108	dermatan sulfate epimerase
FAM26F	chr6	116782532	116784934	2403	family with sequence similarity 26, member F
FAM26E	chr6	116832807	116839709	6903	family with sequence similarity 26, member E
TRAPPC3L	chr6	116817650	116866773	49124	trafficking protein particle complex 3-like
FAM26D	chr6	116850175	116880031	29857	family with sequence similarity 26, member D
RWDD1	chr6	116892529	116914764	22236	RWD domain containing 1
RSPH4A	chr6	116937641	116954148	16508	radial spoke head 4 homolog A (Chlamydomonas)
ZUFSP	chr6	116956780	116989973	33194	zinc finger with UFM1-specific peptidase domain
AX746765	chr6	116999739	117001922	2184	
KPNA5	chr6	117002366	117063030	60665	karyopherin alpha 5 (importin alpha 6)
FAM162B	chr6	117073359	117086886	13528	family with sequence similarity 162, member B
GPRC6A	chr6	117113247	117150220	36974	G protein-coupled receptor, class C, group 6, member A
RFX6	chr6	117198375	117253326	54952	regulatory factor X, 6
VGLL2	chr6	117586720	117594728	8009	vestigial-like family member 2
ROS1	chr6	117609529	117747018	137490	ROS proto-oncogene 1 , receptor tyrosine kinase
GOPC	chr6	117639350	117923705	284356	golgi-associated PDZ and coiled-coil motif containing
DCBLD1	chr6	117803819	117891020	87202	discoidin, CUB and LCCL domain containing 1
AJ420595	chr6	117896482	117897548	1067	
LOC10192791S	chr6	117993975	117996447	2473	uncharacterized LOC101927919
NUS1	chr6	117996616	118031886	35271	nuclear undecaprenyl pyrophosphate synthase 1 homolog (S. cerevisiae)
SLC35F1	chr6	118228688	118638839	410152	solute carrier family 35, member F1
BRD7P3	chr6	118822535	118824996	2462	bromodomain containing 7 pseudogene 3
PLN	chr6	118869441	118881587	12147	phospholamban
CEP85L	chr6	118781934	119031238	249305	centrosomal protein 85kDa-like
LOC10028763Z	chr6	119103870	119104581	712	selenoprotein K pseudogene
MCM9	chr6	119134611	119256327	121717	minichromosome maintenance complex component 9
ASF1A	chr6	119215240	119230335	15096	anti-silencing function 1A histone chaperone
FAM184A	chr6	119280993	119470358	189366	family with sequence similarity 184, member A
MIR548B	chr6	119390211	119390308	98	microRNA 548b
MAN1A1	chr6	119498365	119670931	172567	mannosidase, alpha, class 1A, member 1
LOC285762	chr6	119773711	119812467	38757	uncharacterized LOC285762
C6orf170	chr6	121400626	121655644	255019	TBC1 domain family, member 32
TBC1D32	chr6	121400639	121655646	255008	TBC1 domain family, member 32
GJA1	chr6	121756722	121770890	14169	gap junction protein, alpha 1, 43kDa
HSF2	chr6	122720695	122754264	33570	heat shock transcription factor 2
BC022047	chr6	122757933	122760368	2436	
SERINC1	chr6	122764492	122792952	28461	serine incorporator 1
PKIB	chr6	122793061	123047518	254458	protein kinase (cAMP-dependent, catalytic) inhibitor beta
FABP7	chr6	123100645	123105218	4574	fatty acid binding protein 7, brain
SMPDL3A	chr6	123109970	123130864	20895	sphingomyelin phosphodiesterase, acid-like 3A
CLVS2	chr6	123317115	123394064	76950	clavesin 2
TRDN	chr6	123537483	123958238	420756	triadin

LOC101927990	chr6	123760748	123792901	32154	uncharacterized LOC101927990
AL832096	chr6	123760748	123792901	32154	
NKAIN2	chr6	124124990	125146786	1021797	Na+/K+ transporting ATPase interacting 2
STL	chr6	125229391	125284173	54783	
RNF217-AS1	chr6	125229391	125284173	54783	RNF217 antisense RNA 1 (head to head)
Metazoa_SRP	chr6	125286438	125286740	303	
RNF217	chr6	125283690	125407484	123795	ring finger protein 217
TPD52L1	chr6	125474874	125584644	109771	tumor protein D52-like 1
HDDC2	chr6	125596495	125623282	26788	HD domain containing 2
LOC643623	chr6	125995498	126041364	45867	uncharacterized LOC643623
BC036196	chr6	126039386	126070336	30951	
HEY2	chr6	126068779	126082415	13637	hes-related family bHLH transcription factor with YRPW motif 2
TRNA_Glu	chr6	126101392	126101464	73	
NCOA7	chr6	126102306	126253176	150871	nuclear receptor coactivator 7
HINT3	chr6	126277860	126301389	23530	histidine triad nucleotide binding protein 3
TRMT11	chr6	126307575	126360420	52846	tRNA methyltransferase 11 homolog (S. cerevisiae)
MIR5695	chr6	126433836	126443762	9927	microRNA 5695
CENPW	chr6	126660934	126804466	143533	centromere protein W
Vimentin3	chr6	126923379	126923444	66	
AK127472	chr6	126698800	127440994	742195	
RSPO3	chr6	127440047	127520626	80580	R-spondin 3
RNF146	chr6	127587826	127609705	21880	ring finger protein 146
ECHDC1	chr6	127609856	127664754	54899	enoyl CoA hydratase domain containing 1
KIAA0408	chr6	127759550	127780535	20986	KIAA0408
SOGA3	chr6	127759550	127840500	80951	SOGA family member 3
C6orf58	chr6	127898318	127912963	14646	chromosome 6 open reading frame 58
THEMIS	chr6	128029338	128239776	210439	thymocyte selection associated
LOC101928140	chr6	128349009	128407435	58427	uncharacterized LOC101928140
PTPRK	chr6	128289923	128841870	551948	protein tyrosine phosphatase, receptor type, K
LAMA2	chr6	129204285	129837710	633426	laminin, alpha 2
BC035400	chr6	129800757	129873731	72975	
ARHGAP18	chr6	129898239	130182692	284454	Rho GTPase activating protein 18
TMEM244	chr6	130152388	130182416	30029	transmembrane protein 244
L3MBTL3	chr6	130339727	130462594	122868	l(3)mbt-like 3 (Drosophila)
SAMD3	chr6	130465446	130686570	221125	sterile alpha motif domain containing 3
TMEM200A	chr6	130687425	130764210	76786	transmembrane protein 200A
Mir_562	chr6	131092913	131093012	100	
SMLR1	chr6	131148544	131158276	9733	small leucine-rich protein 1
EPB41L2	chr6	131160487	131384462	223976	erythrocyte membrane protein band 4.1-like 2
AKAP7	chr6	131456825	131604675	147851	A kinase (PRKA) anchor protein 7
ARG1	chr6	131894343	131905472	11130	arginase 1
MED23	chr6	131895105	131949379	54275	mediator complex subunit 23
ENPP3	chr6	131949581	132068550	118970	ectonucleotide pyrophosphatase/phosphodiesterase 3
OR2A4	chr6	132020783	132022541	1759	olfactory receptor, family 2, subfamily A, member 4
CTAGE9	chr6	132029580	132032157	2578	CTAGE family, member 9
ENPP1	chr6	132129155	132216295	87141	ectonucleotide pyrophosphatase/phosphodiesterase 1
DD182001	chr6	132270468	132270491	24	
CTGF	chr6	132269316	132272518	3203	connective tissue growth factor
LINC01013	chr6	132455117	132490514	35398	long intergenic non-protein coding RNA 1013
LOC100507254	chr6	132455117	132490514	35398	long intergenic non-protein coding RNA 1013
MOXD1	chr6	132617193	132722673	105481	monooxygenase, DBH-like 1
STX7	chr6	132778662	132834337	55676	syntaxin 7
TAAR9	chr6	132859426	132860475	1050	trace amine associated receptor 9 (gene/pseudogene)
TAAR8	chr6	132873831	132874860	1030	trace amine associated receptor 8
TAAR6	chr6	132891460	132892498	1039	trace amine associated receptor 6
TAAR5	chr6	132909730	132910877	1148	trace amine associated receptor 5
TAAR3	chr6	132929363	132930441	1079	trace amine associated receptor 3 (gene/pseudogene)
TAAR2	chr6	132938288	132945414	7127	trace amine associated receptor 2
TAAR1	chr6	132966122	132967142	1021	trace amine associated receptor 1
VNN1	chr6	133001996	133035194	33199	vanin 1
VNN3	chr6	133043925	133055904	11980	vanin 3
VNN2	chr6	133065008	133084598	19591	vanin 2
SLC18B1	chr6	133090506	133119747	29242	solute carrier family 18, subfamily B, member 1
SNORD101	chr6	133136445	133136518	74	small nucleolar RNA, C/D box 101
RPS12	chr6	133135707	133138703	2997	ribosomal protein S12
SNORD100	chr6	133137940	133138016	77	small nucleolar RNA, C/D box 100
SNORA33	chr6	133138357	133138490	134	small nucleolar RNA, H/ACA box 33
LINC00326	chr6	133409218	133427717	18500	long intergenic non-protein coding RNA 326
EYA4	chr6	133561735	133853258	291524	EYA transcriptional coactivator and phosphatase 4
AK093513	chr6	133756215	133828795	72581	
BC041459	chr6	133823389	134210120	386732	
TARID	chr6	133823389	134210144	386756	TCF21 antisense RNA inducing promoter demethylation
LINC01312	chr6	134142284	134175130	32847	long intergenic non-protein coding RNA 1312
MGC34034	chr6	134142284	134175130	32847	
TCF21	chr6	134210258	134216675	6418	transcription factor 21
AX747860	chr6	134216640	134219157	2518	
TBPL1	chr6	134273307	134308638	35332	TBP-like 1
SLC2A12	chr6	134308718	134373789	65072	solute carrier family 2 (facilitated glucose transporter), member 12
HMGA1P7	chr6	134435110	134438818	3709	high mobility group AT-hook 1 pseudogene 7
SGK1	chr6	134490383	134639196	148814	serum/glucocorticoid regulated kinase 1

AJ606330	chr6	134749385	134797680	48296	
BC040308	chr6	134749385	134800051	50667	
AJ606328	chr6	134749385	134800051	50667	
LOC101928231	chr6	134750167	134800035	49869	uncharacterized LOC101928231
LINC01010	chr6	134758853	134825158	66306	long intergenic non-protein coding RNA 1010
LOC154092	chr6	134758853	134825158	66306	long intergenic non-protein coding RNA 1010
LOC101928304	chr6	134846455	134861143	14689	uncharacterized LOC101928304
AJ606331	chr6	134846455	134930796	84342	
DQ591448	chr6	134943815	134943843	29	
ALDH8A1	chr6	135238527	135271260	32734	aldehyde dehydrogenase 8 family, member A1
MIR3662	chr6	135300475	135300570	96	microRNA 3662
HBS1L	chr6	135281516	135376036	94521	HBS1-like translational GTPase
MYB	chr6	135502452	135540311	37860	v-myb avian myeloblastosis viral oncogene homolog
AHI1	chr6	135605109	135818903	213795	Abelson helper integration site 1
LINC00271	chr6	135818938	136037193	218256	long intergenic non-protein coding RNA 271
PDE7B	chr6	136172833	136516709	343877	phosphodiesterase 7B
AJ606316	chr6	136175173	136546762	371590	
BC038719	chr6	136364989	136393965	28977	
MTFR2	chr6	136552167	136571449	19283	mitochondrial fission regulator 2
BCLAF1	chr6	136578000	136610989	32990	BCL2-associated transcription factor 1
AB073649	chr6	136692055	136694961	2907	
MAP7	chr6	136663418	136871957	208540	microtubule-associated protein 7
LOC101928461	chr6	136950251	136969336	19086	uncharacterized LOC101928461
CRISPR_DR35	chr6	136972992	136973028	37	
MAP3K5	chr6	136878186	137113656	235471	mitogen-activated protein kinase kinase kinase 5
PEX7	chr6	137143701	137235072	91372	peroxisomal biogenesis factor 7
SLC35D3	chr6	137243401	137246776	3376	solute carrier family 35, member D3
NHEG1	chr6	137303295	137314368	11074	neuroblastoma highly expressed 1
IL20RA	chr6	137321107	137366317	45211	interleukin 20 receptor, alpha
IL22RA2	chr6	137464956	137494785	29830	interleukin 22 receptor, alpha 2
IFNGR1	chr6	137518620	137540567	21948	interferon gamma receptor 1
OLIG3	chr6	137813335	137815531	2197	oligodendrocyte transcription factor 3
LOC10272364	chr6	137986782	137995691	8910	uncharacterized LOC102723649
LOC10050740	chr6	138051306	138060174	8869	uncharacterized LOC100507406
LOC10013047	chr6	138144806	138189370	44565	uncharacterized LOC100130476
TNFAIP3	chr6	138188324	138204451	16128	tumor necrosis factor, alpha-induced protein 3
PERP	chr6	138409641	138428660	19020	PERP, TP53 apoptosis effector
PBOV1	chr6	138537126	138539627	2502	prostate and breast cancer overexpressed 1
KIAA1244	chr6	138483052	138665800	182749	KIAA1244
HEBP2	chr6	138725335	138734582	9248	heme binding protein 2
MIR3145	chr6	138756349	138756431	83	microRNA 3145
NHSL1	chr6	138743180	138893668	150489	NHS-like 1
FLJ46906	chr6	139012804	139018425	5622	uncharacterized LOC441172
LOC100507462	chr6	139046347	139094816	48470	uncharacterized LOC100507462
CCDC28A	chr6	139094656	139114456	19801	coiled-coil domain containing 28A
ECT2L	chr6	139117247	139225207	107961	epithelial cell transforming sequence 2 oncogene-like
REPS1	chr6	139225151	139309398	84248	RALBP1 associated Eps domain containing 1
ABRACL	chr6	139349818	139364439	14622	ABRA C-terminal like
HECA	chr6	139456248	139501946	45699	headcase homolog (Drosophila)
TXLNB	chr6	139561198	139613208	52011	taxilin beta
CITED2	chr6	139693391	139695787	2397	Cbp/p300-interacting transactivator, with Glu/Asp-rich carboxy-terminal domain, 2
LOC645434	chr6	139790131	139795733	5603	uncharacterized LOC645434
LOC10013273	chr6	140092209	140181608	89400	uncharacterized LOC100132735
LOC103352541	chr6	140299486	140312398	12913	uncharacterized LOC103352541
LOC100507477	chr6	140297455	140414858	117404	uncharacterized LOC100507477
BC038188	chr6	140297466	140414858	117393	
MIR3668	chr6	140526388	140526463	76	microRNA 3668
MIR4465	chr6	141004950	141005020	71	microRNA 4465
AK097143	chr6	141902805	142351554	448750	
JA611295	chr6	142334791	142334865	75	
JA611294	chr6	142334796	142334860	65	
NMBR	chr6	142396744	142409936	13193	neuromedin B receptor
VTA1	chr6	142468298	142542085	73788	vesicle (multivesicular body) trafficking 1
TRNA_Gly	chr6	142578775	142578846	72	
GPR126	chr6	142623055	142767403	144349	G protein-coupled receptor 126
LOC153910	chr6	142847591	142959026	111436	uncharacterized LOC153910
HIVEP2	chr6	143072603	143266338	193736	human immunodeficiency virus type I enhancer binding protein 2
LINC01277	chr6	143287558	143358719	71162	long intergenic non-protein coding RNA 1277
LOC10050748	chr6	143287558	143358719	71162	long intergenic non-protein coding RNA 1277
AIG1	chr6	143380499	143661480	280982	androgen-induced 1
ADAT2	chr6	143743968	143771841	27874	adenosine deaminase, tRNA-specific 2
PEX3	chr6	143771917	143811751	39835	peroxisomal biogenesis factor 3
FUCA2	chr6	143815948	143833020	17073	fucosidase, alpha-L- 2, plasma
LOC285740	chr6	143875251	143890476	15226	uncharacterized LOC285740
PHACTR2	chr6	143929316	144152322	223007	phosphatase and actin regulator 2
LTV1	chr6	144164507	144184943	20437	LTV1 ribosome biogenesis factor
AX746989	chr6	144175205	144177090	1886	
ZC2HC1B	chr6	144178439	144259483	81045	zinc finger, C2HC-type containing 1B
PLAGL1	chr6	144261436	144385736	124301	pleiomorphic adenoma gene-like 1
HYMAI	chr6	144324033	144329867	5835	hydatidiform mole associated and imprinted (non-protein coding)

SF3B5	chr6	144416017	144416754	738	splicing factor 3b, subunit 5, 10kDa
STX11	chr6	144471653	144513076	41424	syntaxin 11
UTRN	chr6	144607489	145174170	566682	utrophin
EPM2A	chr6	145946439	146057122	110684	epilepsy, progressive myoclonus type 2A, Lafora disease (laforin)
FLJ44955	chr6	146110405	146113109	2705	
FBXO30	chr6	146119271	146135921	16651	F-box protein 30
LOC100507557	chr6	146056004	146207721	151718	uncharacterized LOC100507557
SHPRH	chr6	146205944	146285233	79290	SNF2 histone linker PHD RING helicase
GRM1	chr6	146348781	146758734	409954	glutamate receptor, metabotropic 1
RAB32	chr6	146864827	146876086	11260	RAB32, member RAS oncogene family
FW339973	chr6	146870894	146873504	2611	
LOC101928661	chr6	146915651	146920067	4417	uncharacterized LOC101928661
ADGB	chr6	146920135	147136597	216463	androglobin
KATNBL1P6	chr6	147122804	147124960	2157	katanin p80 subunit B-like 1 pseudogene 6
LOC729176	chr6	147122804	147124960	2157	katanin p80 subunit B-like 1 pseudogene 6
STXBP5-AS1	chr6	147162524	147525750	363227	STXBP5 antisense RNA 1
STXBP5	chr6	147525493	147711612	186120	syntaxin binding protein 5 (tomosyn)
BC043173	chr6	147708803	147711603	2801	
SAMD5	chr6	147829827	147891157	61331	sterile alpha motif domain containing 5
BC047626	chr6	148048811	148051503	2693	
SASH1	chr6	148663728	148873184	209457	SAM and SH3 domain containing 1
UST	chr6	149068270	149398126	329857	uronyl-2-sulfotransferase
LOC100128176	chr6	149275396	149285820	10425	uncharacterized LOC100128176
BC041998	chr6	149539061	149544218	5158	
TAB2	chr6	149539059	149732749	193691	TGF-beta activated kinase 1/MAP3K7 binding protein 2
SUMO4	chr6	149721494	149722182	689	small ubiquitin-like modifier 4
ZC3H12D	chr6	149768765	149806148	37384	zinc finger CCCH-type containing 12D
PPIL4	chr6	149825630	149867238	41609	peptidylprolyl isomerase (cyclophilin)-like 4
GINM1	chr6	149887527	149912067	24541	glycoprotein integral membrane 1
RPS18P9	chr6	149914751	149915720	970	ribosomal protein S18 pseudogene 9
KATNA1	chr6	149916010	149969940	53931	katanin p60 (ATPase containing) subunit A 1
LATS1	chr6	149979288	150039392	60105	large tumor suppressor kinase 1
NUP43	chr6	150045451	150067708	22258	nucleoporin 43kDa
PCMT1	chr6	150070830	150132557	61728	protein-L-isoaspartate (D-aspartate) O-methyltransferase
LRP11	chr6	150139893	150185480	45588	low density lipoprotein receptor-related protein 11
RAET1E	chr6	150204510	150217195	12686	retinoic acid early transcript 1E
RAET1E-AS1	chr6	150184633	150240644	56012	RAET1E antisense RNA 1
LOC100652739	chr6	150184633	150240644	56012	RAET1E antisense RNA 1
RAET1G	chr6	150238013	150244214	6202	retinoic acid early transcript 1G
BC040898	chr6	150255662	150257455	1794	
ULBP2	chr6	150263135	150270368	7234	UL16 binding protein 2
ULBP1	chr6	150285142	150294846	9705	UL16 binding protein 1
RAET1K	chr6	150319154	150326280	7127	retinoic acid early transcript 1K pseudogene
RAET1L	chr6	150341265	150346668	5404	retinoic acid early transcript 1L
ULBP3	chr6	150383340	150390283	6944	UL16 binding protein 3
PPP1R14C	chr6	150464187	150571528	107342	protein phosphatase 1, regulatory (inhibitor) subunit 14C
IYD	chr6	150690027	150725765	35739	iodotyrosine deiodinase
PLEKHG1	chr6	150920998	151164799	243802	pleckstrin homology domain containing, family G (with RhoGef domain) member 1
MTHFD1L	chr6	151186814	151423023	236210	methylenetetrahydrofolate dehydrogenase (NADP+ dependent) 1-like
LOC102723831	chr6	151517807	151549592	31786	uncharacterized LOC102723831
BC031960	chr6	151517807	151549592	31786	
AKAP12	chr6	151561133	151679694	118562	A kinase (PRKA) anchor protein 12
C6orf211	chr6	151773392	151791234	17843	chromosome 6 open reading frame 211
CCDC170	chr6	151815174	151942328	127155	coiled-coil domain containing 170
ESR1	chr6	151977829	152424408	446580	estrogen receptor 1
SYNE1	chr6	152656412	152702497	46086	spectrin repeat containing, nuclear envelope 1
SYNE1	chr6	152442818	152958534	515717	spectrin repeat containing, nuclear envelope 1
SYNE1-AS1	chr6	152701664	152702847	1184	SYNE1 antisense RNA 1
MYCT1	chr6	153019029	153045715	26687	myc target 1
VIP	chr6	153071931	153080902	8972	vasoactive intestinal peptide
FBXO5	chr6	153291657	153304740	13084	F-box protein 5
MTRF1L	chr6	153308399	153323925	15527	mitochondrial translational release factor 1-like
RGS17	chr6	153332031	153452389	120359	regulator of G-protein signaling 17
OPRM1	chr6	154331630	154568001	236372	opioid receptor, mu 1
IPCEF1	chr6	154475617	154831753	356137	interaction protein for cytohesin exchange factors 1
CNKSR3	chr6	154726432	154831753	105322	CNKSR family member 3
SCAF8	chr6	155054511	155155378	100868	SR-related CTD-associated factor 8
AK124484	chr6	155184661	155230000	45340	
U8	chr6	155226209	155226340	132	small nucleolar RNA, C/D box 118
TIAM2	chr6	155153830	155578857	425028	T-cell lymphoma invasion and metastasis 2
AB075492	chr6	155572433	155575064	2632	
AK022993	chr6	155574273	155577858	3586	
CLDN20	chr6	155585146	155597682	12537	claudin 20
TFB1M	chr6	155577263	155635617	58355	transcription factor B1, mitochondrial
NOX3	chr6	155716501	155777037	60537	NADPH oxidase 3
MIR4466	chr6	157100811	157100865	55	microRNA 4466
ARID1B	chr6	157099063	157531913	432851	AT rich interactive domain 1B (SWI1-like)
TMEM242	chr6	157710053	157745291	35239	transmembrane protein 242
ZDHHC14	chr6	157802556	158094977	292422	zinc finger, DHHC-type containing 14
SNX9	chr6	158244202	158366109	121908	sorting nexin 9

SYNJ2-IT1	chr6	158422138	158423415	1278	SYNJ2 intronic transcript 1 (non-protein coding)
AK026758	chr6	158448398	158450058	1661	
SYNJ2	chr6	158402887	158520207	117321	synaptojanin 2
SERAC1	chr6	158530535	158589312	58778	serine active site containing 1
GTF2H5	chr6	158589378	158620376	30999	general transcription factor IIH, polypeptide 5
TULP4	chr6	158733691	158932856	199166	tubby like protein 4
DQ581726	chr6	158981118	158981146	29	
TMEM181	chr6	158957467	159056467	99001	transmembrane protein 181
MIR7161	chr6	159030738	159030822	85	microRNA 7161
DQ586009	chr6	159031785	159031813	29	
DYNLT1	chr6	159057506	159065818	8313	dynein, light chain, Tctex-type 1
SYTL3	chr6	159071045	159185908	114864	synaptotagmin-like 3
EZR	chr6	159186772	159240456	53685	ezrin
AX747826	chr6	159219681	159221893	2213	
EZR-AS1	chr6	159239010	159243272	4263	EZR antisense RNA 1
OSTCP1	chr6	159262157	159278664	16508	oligosaccharyltransferase complex subunit pseudogene 1
C6orf99	chr6	159309618	159331385	21768	chromosome 6 open reading frame 99
RSPH3	chr6	159398265	159421198	22934	radial spoke 3 homolog (Chlamydomonas)
TAGAP	chr6	159455500	159466184	10685	T-cell activation RhoGTPase activating protein
LOC101929122	chr6	159586930	159591039	4110	uncharacterized LOC101929122
FNDC1	chr6	159590428	159693140	102713	fibronectin type III domain containing 1
LOC102724053	chr6	159804562	159817473	12912	uncharacterized LOC102724053
AK130765	chr6	160007986	160010201	2216	
BC016015	chr6	160100148	160101403	1256	
SOD2	chr6	160100148	160114353	14206	superoxide dismutase 2, mitochondrial
WTAP	chr6	160148029	160177352	29324	Wilms tumor 1 associated protein
LOC100129518	chr6	160181290	160183364	2075	uncharacterized LOC100129518
ACAT2	chr6	160182988	160200087	17100	acetyl-CoA acetyltransferase 2
SNORA20	chr6	160201281	160201413	133	small nucleolar RNA, H/ACA box 20
TCP1	chr6	160199529	160210735	11207	t-complex 1
SNORA29	chr6	160206625	160206765	141	small nucleolar RNA, H/ACA box 29
MRPL18	chr6	160211021	160219461	8441	mitochondrial ribosomal protein L18
PNLDC1	chr6	160221280	160241736	20457	poly(A)-specific ribonuclease (PARN)-like domain containing 1
MAS1	chr6	160327973	160329107	1135	MAS1 proto-oncogene, G protein-coupled receptor
AIRN	chr6	160424322	160428696	4375	antisense of IGF2R non-protein coding RNA
IGF2R	chr6	160390130	160527583	137454	insulin-like growth factor 2 receptor
SLC22A1	chr6	160542862	160579750	36889	solute carrier family 22 (organic cation transporter), member 1
SLC22A2	chr6	160637793	160679963	42171	solute carrier family 22 (organic cation transporter), member 2
SLC22A3	chr6	160769404	160873611	104208	solute carrier family 22 (organic cation transporter), member 3
LPAL2	chr6	160887586	160932156	44571	lipoprotein, Lp(a)-like 2, pseudogene
LPA	chr6	160952514	161087407	134894	lipoprotein, Lp(a)
PLG	chr6	161123224	161175085	51862	plasminogen
MAP3K4	chr6	161412758	161538417	125660	mitogen-activated protein kinase kinase kinase 4
AGPAT4-IT1	chr6	161581163	161583014	1852	AGPAT4 intronic transcript 1 (non-protein coding)
AGPAT4	chr6	161551056	161695107	144052	1-acylglycerol-3-phosphate O-acyltransferase 4
PARK2	chr6	161768589	163148834	1380246	parkin RBR E3 ubiquitin protein ligase
PACRG	chr6	163148163	163736524	588362	PARK2 co-regulated
LOC101929239	chr6	163464011	163475193	11183	uncharacterized LOC101929239
AK058177	chr6	163464011	163475193	11183	
AK296276	chr6	163586445	163612840	26396	
LOC729658	chr6	163602676	163613034	10359	uncharacterized LOC729658
AK311212	chr6	163603634	163613034	9401	
DKFZp451B082	chr6	163759373	163768065	8693	uncharacterized LOC401282
CAHM	chr6	163834096	163834982	887	colon adenocarcinoma hypermethylated (non-protein coding)
QKI	chr6	163835674	163999628	163955	QKI, KH domain containing, RNA binding
AK093114	chr6	164092635	164195657	103023	
AK090788	chr6	165206863	165235542	28680	
C6orf118	chr6	165693152	165723111	29960	chromosome 6 open reading frame 118
PDE10A	chr6	165740775	166075588	334814	phosphodiesterase 10A
AK090688	chr6	166193756	166399984	206229	
LINC00473	chr6	166337535	166401527	63993	long intergenic non-protein coding RNA 473
LINC00602	chr6	166401038	166403103	2066	long intergenic non-protein coding RNA 602
T	chr6	166571145	166582157	11013	T, brachyury homolog (mouse)
LOC101929297	chr6	166650456	166670432	19977	uncharacterized LOC101929297
PRR18	chr6	166719167	166721871	2705	proline rich 18
SFT2D1	chr6	166733516	166755991	22476	SFT2 domain containing 1
LOC100289495	chr6	166756118	166764957	8840	uncharacterized LOC100289495
MPC1	chr6	166778407	166796501	18095	mitochondrial pyruvate carrier 1
RPS6KA2-IT1	chr6	166874150	166878871	4722	RPS6KA2 intronic transcript 1 (non-protein coding)
MIR1913	chr6	166922841	166922921	81	microRNA 1913
RPS6KA2	chr6	166822853	167275771	452919	ribosomal protein S6 kinase, 90kDa, polypeptide 2
RPS6KA2-AS1	chr6	167317185	167318557	1373	RPS6KA2 antisense RNA 1
MIR3939	chr6	167411294	167411400	107	microRNA 3939
FGFR1OP	chr6	167412804	167455906	43103	FGFR1 oncogene partner
CCR6	chr6	167412815	167552629	139815	chemokine (C-C motif) receptor 6
GPR31	chr6	167570359	167571319	961	G protein-coupled receptor 31
TCP10L2	chr6	167584080	167596396	12317	t-complex 10-like 2
UNC93A	chr6	167704802	167729502	24701	unc-93 homolog A (C. elegans)
TTLL2	chr6	167738573	167756177	17605	tubulin tyrosine ligase-like family, member 2
TCP10	chr6	167770520	167797998	27479	t-complex 10

LOC401286	chr6	168067521	168079903	12383	uncharacterized LOC401286
AK127120	chr6	168067523	168079903	12381	
LOC441178	chr6	168080305	168084736	4432	uncharacterized LOC441178
AL832737	chr6	168080305	168096970	16666	
C6orf123	chr6	168185218	168197539	12322	chromosome 6 open reading frame 123
MLLT4-AS1	chr6	168224569	168227476	2908	MLLT4 antisense RNA 1 (head to head)
MLL/AF6 fusion	chr6	168265223	168265426	204	
MLLT4	chr6	168227670	168372700	145031	myeloid/lymphoid or mixed-lineage leukemia (trithorax homolog, Drosophila); translocated to, 4
BX649158	chr6	168362689	168365707	3019	
HGC6.3	chr6	168376603	168377619	1017	uncharacterized LOC100128124
HGC6.1.1	chr6	168393501	168397757	4257	non-protein coding RNA 300
KIF25-AS1	chr6	168394866	168397757	2892	KIF25 antisense RNA 1
KIF25	chr6	168399771	168445769	45999	kinesin family member 25
FRMD1	chr6	168456463	168482237	25775	FERM domain containing 1
LOC10192942(	chr6	168643617	168663258	19642	uncharacterized LOC101929420
DACT2	chr6	168693509	168720434	26926	dishevelled-binding antagonist of beta-catenin 2
SMOC2	chr6	168841830	169068674	226845	SPARC related modular calcium binding 2
LOC101929504	chr6	169575398	169582835	7438	uncharacterized LOC101929504
AF086258	chr6	169575398	169582835	7438	
THBS2	chr6	169615874	169654209	38336	thrombospondin 2
AK055570	chr6	169773775	169788480	14706	
WDR27	chr6	169857302	170102159	244858	WD repeat domain 27
AK128656	chr6	170060912	170063234	2323	
C6orf120	chr6	170102232	170106402	4171	chromosome 6 open reading frame 120
PHF10	chr6	170104001	170124106	20106	PHD finger protein 10
TCTE3	chr6	170140214	170151638	11425	t-complex-associated-testis-expressed 3
AK023627	chr6	170149607	170151679	2073	
C6orf70	chr6	170151720	170181617	29898	ER membrane-associated RNA degradation
ERMARD	chr6	170151717	170181680	29964	ER membrane-associated RNA degradation
LINC00242	chr6	170188885	170198921	10037	long intergenic non-protein coding RNA 242
LINC00574	chr6	170190168	170202969	12802	long intergenic non-protein coding RNA 574
LOC154449	chr6	170563421	170571657	8237	uncharacterized LOC154449
LOC285804	chr6	170575756	170585850	10095	uncharacterized LOC285804
FLJ38122	chr6	170581561	170588554	6994	uncharacterized LOC401289
DLL1	chr6	170591293	170599697	8405	delta-like 1 (Drosophila)
MIR4644	chr6	170639848	170639932	85	microRNA 4644
FAM120B	chr6	170599790	170716159	116370	family with sequence similarity 120B
PSMB1	chr6	170780106	170862417	82312	proteasome (prosome, macropain) subunit, beta type, 1
TBP	chr6	170863420	170881958	18539	TATA box binding protein
PDCD2	chr6	170884659	170893780	9122	programmed cell death 2
BC036251	chr6	171004401	171006035	1635	
TCR V3-JP2-C(	chr7	38288826	38295990	7165	
TRGC2	chr7	38279180	38356821	77642	T cell receptor gamma constant 2
TARP	chr7	38279180	38357589	78410	TCR gamma alternate reading frame protein
TCRGC2	chr7	38288843	38356709	67867	T cell receptor gamma constant 2 (2x)
TCRGV	chr7	38339411	38339881	471	
AK096766	chr7	38342348	38344378	2031	
B4GALNT4	chr11	369794	382117	12324	beta-1,4-N-acetyl-galactosaminyl transferase 4
PKP3	chr11	392598	404908	12311	plakophilin 3
SIGIRR	chr11	405715	417397	11683	single immunoglobulin and toll-interleukin 1 receptor (TIR) domain
ANO9	chr11	417929	442011	24083	anoctamin 9
Metazoa_SRP	chr11	440404	440693	290	
PTDSS2	chr11	450279	491387	41109	phosphatidylserine synthase 2
RNH1	chr11	494511	507283	12773	ribonuclease/angiogenin inhibitor 1
HRAS	chr11	532241	535550	3310	Harvey rat sarcoma viral oncogene homolog
LRRC56	chr11	537521	554916	17396	leucine rich repeat containing 56
LMNTD2	chr11	554849	560779	5931	lamin tail domain containing 2
C11orf35	chr11	554849	560779	5931	lamin tail domain containing 2
AX748330	chr11	557594	560107	2514	
BC031953	chr11	559476	560107	632	
RASSF7	chr11	560970	564025	3056	Ras association (RalGDS/AF-6) domain family (N-terminal) member 7
MIR210HG	chr11	565656	568457	2802	MIR210 host gene (non-protein coding)
MIR210	chr11	568088	568198	111	microRNA 210
CD151	chr11	832951	838835	5885	CD151 molecule (Raph blood group)
POLR2L	chr11	839720	842529	2810	polymerase (RNA) II (DNA directed) polypeptide L, 7.6kDa
TSPAN4	chr11	842823	867116	24294	tetraspanin 4
AK126635	chr11	856879	858707	1829	
AX747537	chr11	880866	881475	610	
CHID1	chr11	867356	915058	47703	chitinase domain containing 1
BRSK2	chr11	1411128	1483919	72792	BR serine/threonine kinase 2
MOB2	chr11	1490677	1508009	17333	MOB kinase activator 2
DUSP8	chr11	1575280	1593150	17871	dual specificity phosphatase 8
KRTAP5-1	chr11	1605571	1606513	943	keratin associated protein 5-1
KRTAP5-AS1	chr11	1593970	1620414	26445	KRTAP5-1/KRTAP5-2 antisense RNA 1
KRTAP5-2	chr11	1618406	1619524	1119	keratin associated protein 5-2
KRTAP5-3	chr11	1628794	1629693	900	keratin associated protein 5-3
KRTAP5-4	chr11	1642187	1643368	1182	keratin associated protein 5-4
MIR483	chr11	2155363	2155439	77	microRNA 483
IGF2	chr11	2150341	2170833	20493	insulin-like growth factor 2
IGF2-AS	chr11	2161757	2169896	8140	IGF2 antisense RNA

INS-IGF2	chr11	2150341	2182439	32099	INS-IGF2 readthrough
INS	chr11	2181008	2182439	1432	insulin
TH	chr11	2185158	2193035	7878	tyrosine hydroxylase
MIR4686	chr11	2194292	2194368	77	microRNA 4686
C11orf21	chr11	2317506	2323143	5638	chromosome 11 open reading frame 21
TSPAN32	chr11	2323242	2339430	16189	tetraspanin 32
CD81-AS1	chr11	2349978	2399222	49245	CD81 antisense RNA 1
BC019904	chr11	2349978	2399222	49245	
CD81	chr11	2397406	2418649	21244	CD81 molecule
TSSC4	chr11	2421717	2425108	3392	tumor suppressing subtransferable candidate 4
TRPM5	chr11	2425745	2444275	18531	transient receptor potential cation channel, subfamily M, member 5
KCNQ1	chr11	2466220	2870340	404121	potassium voltage-gated channel, KQT-like subfamily, member 1
KCNQ1OT1	chr11	2629557	2721228	91672	KCNQ1 overlapping transcript 1
KCNQ1DN	chr11	2891262	2893336	2075	KCNQ1 downstream neighbor (non-protein coding)
SLC22A18AS	chr11	2909326	2925175	15850	solute carrier family 22 (organic cation transporter), member 18 antisense
SLC22A18	chr11	2920950	2946476	25527	solute carrier family 22, member 18
ZNF195	chr11	3379156	3400452	21297	zinc finger protein 195
OR7E12P	chr11	3411936	3413145	1210	olfactory receptor, family 7, subfamily E, member 12 pseudogene
TSSC2	chr11	3402190	3430378	28189	tumor suppressing subtransferable candidate 2 pseudogene
LOC650368	chr11	3402190	3430378	28189	tumor suppressing subtransferable candidate 2 pseudogene
AB231779	chr11	3638461	3647749	9289	
TRPC2	chr11	3647689	3658789	11101	transient receptor potential cation channel, subfamily C, member 2, pseudogene
ART5	chr11	3659735	3663546	3812	ADP-ribosyltransferase 5
ART1	chr11	3666360	3685646	19287	ADP-ribosyltransferase 1
CHRNA10	chr11	3686816	3692614	5799	cholinergic receptor, nicotinic, alpha 10 (neuronal)
NUP98	chr11	3696239	3819022	122784	nucleoporin 98kDa
PGAP2	chr11	3818953	3847601	28649	post-GPI attachment to proteins 2
RHOG	chr11	3848207	3862213	14007	ras homolog family member G
MIR4687	chr11	3877291	3877371	81	microRNA 4687
STIM1	chr11	3876932	4114440	237509	stromal interaction molecule 1
RRM1	chr11	4115923	4223883	107961	ribonucleotide reductase M1
LOC100506082	chr11	4208381	4223883	15503	uncharacterized LOC100506082
OR52B4	chr11	4388492	4389616	1125	olfactory receptor, family 52, subfamily B, member 4
TRIM21	chr11	4406126	4414926	8801	tripartite motif containing 21
OR52K2	chr11	4470524	4471591	1068	olfactory receptor, family 52, subfamily K, member 2
OR52K1	chr11	4510108	4511138	1031	olfactory receptor, family 52, subfamily K, member 1
OR52M1	chr11	4566420	4567374	955	olfactory receptor, family 52, subfamily M, member 1
C11orf40	chr11	4592652	4599050	6399	chromosome 11 open reading frame 40
OR52I2	chr11	4608020	4609135	1116	olfactory receptor, family 52, subfamily I, member 2
OR52I1	chr11	4615268	4616243	976	olfactory receptor, family 52, subfamily I, member 1
TRIM68	chr11	4619901	4629489	9589	tripartite motif containing 68
OR51D1	chr11	4660944	4662068	1125	olfactory receptor, family 51, subfamily D, member 1
OR51E1	chr11	4665155	4676716	11562	olfactory receptor, family 51, subfamily E, member 1
OR51E2	chr11	4701400	4719076	17677	olfactory receptor, family 51, subfamily E, member 2
OR51F1	chr11	4790208	4791147	940	olfactory receptor, family 51, subfamily F, member 1
OR52R1	chr11	4824662	4825610	949	olfactory receptor, family 52, subfamily R, member 1
OR51F2	chr11	4842615	4843644	1030	olfactory receptor, family 51, subfamily F, member 2
OR51S1	chr11	4869466	4870438	973	olfactory receptor, family 51, subfamily S, member 1
OR51T1	chr11	4903048	4904113	1066	olfactory receptor, family 51, subfamily T, member 1
OR51A7	chr11	4928599	4929538	940	olfactory receptor, family 51, subfamily A, member 7
OR51G2	chr11	4935948	4936893	946	olfactory receptor, family 51, subfamily G, member 2
OR51G1	chr11	4944603	4945569	967	olfactory receptor, family 51, subfamily G, member 1
OR51A4	chr11	4967388	4968330	943	olfactory receptor, family 51, subfamily A, member 4
OR51A2	chr11	4976001	4976943	943	olfactory receptor, family 51, subfamily A, member 2
MMP26	chr11	5009423	5013659	4237	matrix metallopeptidase 26
OR51L1	chr11	5020212	5021160	949	olfactory receptor, family 51, subfamily L, member 1
OR52J3	chr11	5067755	5068691	937	olfactory receptor, family 52, subfamily J, member 3
OR52E2	chr11	5079879	5080857	979	olfactory receptor, family 52, subfamily E, member 2
DQ656008	chr11	5142384	5145743	3360	
OR52A5	chr11	5152921	5153872	952	olfactory receptor, family 52, subfamily A, member 5
OR52A1	chr11	5172660	5173599	940	olfactory receptor, family 52, subfamily A, member 1
OR52Z1	chr11	5199340	5199783	444	olfactory receptor, family 52, subfamily Z, member 1 (gene/pseudogene)
OR51V1	chr11	5220964	5221930	967	olfactory receptor, family 51, subfamily V, member 1
CoTC_ribozyme	chr11	5245677	5245869	193	
DL074624	chr11	5246721	5246744	24	
HBB	chr11	5246695	5248301	1607	hemoglobin, beta
HBD	chr11	5254058	5255858	1801	hypophosphatemic bone disease
HBBP1	chr11	5263184	5264822	1639	hemoglobin, beta pseudogene 1
BGLT3	chr11	5265783	5266776	994	beta globin locus transcript 3 (non-protein coding)
HBG1	chr11	5269501	5271087	1587	hemoglobin, gamma A
OR51B4	chr11	5322243	5323176	934	olfactory receptor, family 51, subfamily B, member 4
OR51B2	chr11	5344527	5345582	1056	olfactory receptor, family 51, subfamily B, member 2
OR51B6	chr11	5372737	5373676	940	olfactory receptor, family 51, subfamily B, member 6
HBE1	chr11	5289579	5526882	237304	hemoglobin, epsilon 1
OR51M1	chr11	5410606	5411664	1059	olfactory receptor, family 51, subfamily M, member 1
OR51Q1	chr11	5443340	5444436	1097	olfactory receptor, family 51, subfamily Q, member 1
OR51B5	chr11	5362112	5526882	164771	olfactory receptor, family 51, subfamily B, member 5
OR51I1	chr11	5461771	5462783	1013	olfactory receptor, family 51, subfamily I, member 1
HBG2	chr11	5269501	5667011	397511	hemoglobin, gamma G
OR51I2	chr11	5474637	5475707	1071	olfactory receptor, family 51, subfamily I, member 2

OR52D1	chr11	5509914	5510978	1065	olfactory receptor, family 52, subfamily D, member 1
UBQLN3	chr11	5528529	5531153	2625	ubiquilin 3
UBQLNL	chr11	5535622	5537956	2335	ubiquilin-like
OR52H1	chr11	5565790	5566753	964	olfactory receptor, family 52, subfamily H, member 1
OR52B6	chr11	5602106	5603114	1009	olfactory receptor, family 52, subfamily B, member 6
TRIM6	chr11	5617330	5634188	16859	tripartite motif containing 6
TRIM6-TRIM34	chr11	5617864	5665625	47762	TRIM6-TRIM34 readthrough
TRIM34	chr11	5641173	5665625	24453	tripartite motif containing 34
TRIpartite motif	chr11	5686441	5687610	1170	
OR56B1	chr11	5757677	5758768	1092	olfactory receptor, family 56, subfamily B, member 1
TRIM22	chr11	5710816	5821759	110944	tripartite motif containing 22
OR52N4	chr11	5775922	5776959	1038	olfactory receptor, family 52, subfamily N, member 4
OR52N5	chr11	5798863	5799897	1035	olfactory receptor, family 52, subfamily N, member 5
OR52N1	chr11	5809083	5810046	964	olfactory receptor, family 52, subfamily N, member 1
TRIM5	chr11	5684424	5959849	275426	tripartite motif containing 5
OR52N2	chr11	5841565	5842531	967	olfactory receptor, family 52, subfamily N, member 2
OR52E6	chr11	5862185	5863127	943	olfactory receptor, family 52, subfamily E, member 6
OR52E8	chr11	5877978	5878932	955	olfactory receptor, family 52, subfamily E, member 8
OR52E4	chr11	5905522	5906461	940	olfactory receptor, family 52, subfamily E, member 4
OR56A3	chr11	5968576	5969524	949	olfactory receptor, family 56, subfamily A, member 3
OR56A5	chr11	5988782	5989724	943	olfactory receptor, family 56, subfamily A, member 5
OR52L1	chr11	6007121	6008215	1095	olfactory receptor, family 52, subfamily L, member 1
OR56A4	chr11	6023280	6024378	1099	olfactory receptor, family 56, subfamily A, member 4
OR56A1	chr11	6047900	6048971	1072	olfactory receptor, family 56, subfamily A, member 1
OR56B4	chr11	6129008	6129968	961	olfactory receptor, family 56, subfamily B, member 4
OLFR690	chr11	6172912	6173819	908	
OR52B2	chr11	6190584	6191556	973	olfactory receptor, family 52, subfamily B, member 2
OR52W1	chr11	6220453	6221416	964	olfactory receptor, family 52, subfamily W, member 1
C11orf42	chr11	6226797	6232365	5569	chromosome 11 open reading frame 42
FAM160A2	chr11	6232563	6255941	23379	family with sequence similarity 160, member A2
CNGA4	chr11	6255994	6265709	9716	cyclic nucleotide gated channel alpha 4
CCKBR	chr11	6280903	6293357	12455	cholecystokinin B receptor
PRKCDBP	chr11	6340175	6341740	1566	protein kinase C, delta binding protein
DNHD1	chr11	6518525	6593254	74730	dynein heavy chain domain 1
GVINP1	chr11	6734380	6767670	33291	GTPase, very large interferon inducible pseudogene 1
OR2AG2	chr11	6789237	6790188	952	olfactory receptor, family 2, subfamily AG, member 2
OR2AG1	chr11	6806247	6807245	999	olfactory receptor, family 2, subfamily AG, member 1
OR6A2	chr11	6815755	6817139	1385	olfactory receptor, family 6, subfamily A, member 2
OR10A5	chr11	6866913	6867867	955	olfactory receptor, family 10, subfamily A, member 5
OR10A2	chr11	6890985	6891897	913	olfactory receptor, family 10, subfamily A, member 2
OR10A4	chr11	6897855	6898850	996	olfactory receptor, family 10, subfamily A, member 4
OR2D2	chr11	6912804	6913731	928	olfactory receptor, family 2, subfamily D, member 2
OR2D3	chr11	6942232	6943225	994	olfactory receptor, family 2, subfamily D, member 3
ZNF215	chr11	6947653	7005861	58209	zinc finger protein 215
ZNF214	chr11	7020548	7041586	21039	zinc finger protein 214
NLRP14	chr11	7041699	7092757	51059	NLR family, pyrin domain containing 14
JB175177	chr11	7078055	7078075	21	
RBMXL2	chr11	7110164	7112379	2216	RNA binding motif protein, X-linked-like 2
SYT9	chr11	7260008	7490276	230269	synaptotagmin IX
BC040358	chr11	7458867	7487042	28176	
LOC100506258	chr11	7458867	7487066	28200	uncharacterized LOC100506258
AK128569	chr11	7448496	7526103	77608	
OLFML1	chr11	7506599	7532606	26008	olfactomedin-like 1
PPFIBP2	chr11	7534995	7678358	143364	PTPRF interacting protein, binding protein 2 (liprin beta 2)
CYB5R2	chr11	7686325	7694994	8670	cytochrome b5 reductase 2
OVCH2	chr11	7711154	7727941	16788	ovochymase 2 (gene/pseudogene)
PJCG1	chr11	7794439	7795197	759	
OR5P2	chr11	7817520	7818489	970	olfactory receptor, family 5, subfamily P, member 2
OR5P3	chr11	7846583	7847519	937	olfactory receptor, family 5, subfamily P, member 3
OR5E1P	chr11	7870597	7871118	522	olfactory receptor, family 5, subfamily E, member 1 pseudogene
LOC283299	chr11	7872297	7927502	55206	uncharacterized LOC283299
U6	chr11	7910728	7910826	99	RNA, U6 small nuclear 50, pseudogene
OR10A6	chr11	7949264	7950209	946	olfactory receptor, family 10, subfamily A, member 6
OR10A3	chr11	7960122	7961067	946	olfactory receptor, family 10, subfamily A, member 3
NLRP10	chr11	7981155	7985059	3905	NLR family, pyrin domain containing 10
EIF3F	chr11	8008444	8017719	9276	eukaryotic translation initiation factor 3, subunit F
BC027619	chr11	8032824	8038036	5213	
CASC23	chr11	8032824	8038056	5233	cancer susceptibility candidate 23 (non-protein coding)
TUB	chr11	8040790	8127654	86865	tubby bipartite transcription factor
TUB-AS1	chr11	8081584	8090920	9337	TUB antisense RNA 1
AK055772	chr11	8081584	8090920	9337	
RIC3	chr11	8127596	8190590	62995	RIC3 acetylcholine receptor chaperone
LMO1	chr11	8245850	8290182	44333	LIM domain only 1 (rhombotin 1)
STK33	chr11	8413412	8615836	202425	serine/threonine kinase 33
SCARNA20	chr11	8576562	8576693	132	small Cajal body-specific RNA 20
TRIM66	chr11	8633583	8693413	59831	tripartite motif containing 66
BC068088	chr11	8693132	8701271	8140	
SNORA3	chr11	8705773	8705903	131	small nucleolar RNA, H/ACA box 45A
SNORA45A	chr11	8705773	8705903	131	small nucleolar RNA, H/ACA box 45A
SNORA45	chr11	8706985	8707116	132	small nucleolar RNA, H/ACA box 45B

SNORA45B	chr11	8706985	8707116	132	small nucleolar RNA, H/ACA box 45B
RPL27A	chr11	8703994	8711419	7426	ribosomal protein L27a
LOC102724784	chr11	8790324	8831823	41500	uncharacterized LOC102724784
ST5	chr11	8714898	8932498	217601	suppression of tumorigenicity 5
5S_rRNA	chr11	8866809	8866905	97	
AKIP1	chr11	8932700	8941626	8927	A kinase (PRKA) interacting protein 1
C11orf16	chr11	8941622	8954553	12932	chromosome 11 open reading frame 16
ASCL3	chr11	8959118	8964580	5463	achaete-scute family bHLH transcription factor 3
TMEM9B	chr11	8968747	8986558	17812	TMEM9 domain family, member B
TMEM9B-AS1	chr11	8986221	8997830	11610	TMEM9B antisense RNA 1
NRIP3	chr11	9002122	9025596	23475	nuclear receptor interacting protein 3
SCUBE2	chr11	9041046	9113150	72105	signal peptide, CUB domain, EGF-like 2
MIR5691	chr11	9111858	9111926	69	microRNA 5691
KRT8P41	chr11	9115909	9117737	1829	keratin 8 pseudogene 41
DENND5A	chr11	9160374	9286873	126500	DENN/MADD domain containing 5A
TRNA_Leu	chr11	9296789	9296863	75	
TMEM41B	chr11	9302200	9336315	34116	transmembrane protein 41B
F379	chr12	67610	69070	1461	chromosome 1 F379 retina specific protein
FAM138D	chr12	67606	69079	1474	family with sequence similarity 138, member D
DKFZp434K132	chr12	74271	87703	13433	hypothetical protein LOC58481
LOC100288778	chr12	73879	91263	17385	WAS protein family homolog 1 pseudogene
DDX11L11	chr12	91352	93018	1667	DEAD/H (Asp-Glu-Ala-Asp/His) box helicase 11 like 11
IQSEC3	chr12	176048	287625	111578	IQ motif and Sec7 domain 3
KDM5A	chr12	389222	498621	109400	lysine (K)-specific demethylase 5A
CCDC77	chr12	498515	551806	53292	coiled-coil domain containing 77
B4GALNT3	chr12	569542	671058	101517	beta-1,4-N-acetyl-galactosaminyl transferase 3
U4atac	chr12	890298	890424	127	
WNK1	chr12	862088	1020618	158531	WNK lysine deficient protein kinase 1
HSN2	chr12	976959	978360	1402	hereditary sensory neuropathy, type II
AK128619	chr12	1011721	1016190	4470	
RAD52	chr12	1020901	1099219	78319	RAD52 homolog (S. cerevisiae)
BC039168	chr12	1084462	1099219	14758	
ERC1	chr12	1100373	1605099	504727	ELKS/RAB6-interacting/CAST family member 1
LINC00942	chr12	1609656	1613590	3935	long intergenic non-protein coding RNA 942
AX746535	chr12	1615335	1616484	1150	
FBXL14	chr12	1675158	1703331	28174	F-box and leucine-rich repeat protein 14
WNT5B	chr12	1683822	1756378	72557	wingless-type MMTV integration site family, member 5B
MIR3649	chr12	1769480	1769546	67	microRNA 3649
ADIPOR2	chr12	1800246	1897845	97600	adiponectin receptor 2
LRTM2	chr12	1929432	1945918	16487	leucine-rich repeats and transmembrane domains 2
CACNA2D4	chr12	1901122	2027870	126749	calcium channel, voltage-dependent, alpha 2/delta subunit 4
LINC00940	chr12	2038367	2045742	7376	long intergenic non-protein coding RNA 940
CACNA1C	chr12	2080228	2080366	139	calcium channel, voltage-dependent, L type, alpha 1C subunit
DCP1B	chr12	2050756	2113677	62922	decapping mRNA 1B
CACNA1C-IT2	chr12	2157517	2158628	1112	CACNA1C intronic transcript 2 (non-protein coding)
AK308652	chr12	2229490	2229863	374	
CACNA1C-AS4	chr12	2329702	2332647	2946	CACNA1C antisense RNA 4
CACNA1C-IT3	chr12	2378941	2397911	18971	CACNA1C intronic transcript 3 (non-protein coding)
CACNA1C	chr12	2162415	2807115	644701	calcium channel, voltage-dependent, L type, alpha 1C subunit
LOC100507424	chr12	2945981	2968961	22981	uncharacterized LOC100507424
FOXM1	chr12	2966846	2986321	19476	forkhead box M1
RHNO1	chr12	2985423	2998691	13269	RAD9-HUS1-RAD1 interacting nuclear orphan 1
TULP3	chr12	2986364	3050306	63943	tubby like protein 3
TEAD4	chr12	3068477	3149842	81366	TEA domain family member 4
TSPAN9	chr12	3186520	3395730	209211	tetraspanin 9
AK056228	chr12	3405367	3409390	4024	
AK095365	chr12	3427883	3434509	6627	
BC047090	chr12	3445188	3445980	793	
DQ574680	chr12	3553532	3553563	32	
DQ597527	chr12	3565649	3565688	40	
DQ591569	chr12	3567721	3570074	2354	
DQ590472	chr12	3575436	3575477	42	
DQ590289	chr12	3579507	3579548	42	
DQ591331	chr12	3579697	3579731	35	
DQ599425	chr12	3580294	3580328	35	
DQ584103	chr12	3581012	3581048	37	
DQ583990	chr12	3581870	3581899	30	
DQ573743	chr12	3584206	3584271	66	
DQ594563	chr12	3584318	3584352	35	
DQ601344	chr12	3584494	3584528	35	
DQ600002	chr12	3585019	3585049	31	
DQ570426	chr12	3585610	3585642	33	
DQ589173	chr12	3586923	3586953	31	
DQ587099	chr12	3586955	3586984	30	
DQ594785	chr12	3587759	3587799	41	
DQ588300	chr12	3590068	3590097	30	
DQ596092	chr12	3590833	3590862	30	
DQ583138	chr12	3591678	3591708	31	
DQ579489	chr12	3592511	3592544	34	
PRMT8	chr12	3490514	3703138	212625	protein arginine methyltransferase 8

AK125333	chr12	3592879	3601985	9107	
EFCAB4B	chr12	3716317	3862366	146050	
CRACR2A	chr12	3724493	3862366	137874	calcium release activated channel regulator 2A
PARP11	chr12	3918026	3982614	64589	poly (ADP-ribose) polymerase family, member 11
CCND2-AS1	chr12	4357932	4385350	27419	CCND2 antisense RNA 1
CCND2	chr12	4382901	4414522	31622	cyclin D2
C12orf5	chr12	4430358	4469190	38833	chromosome 12 open reading frame 5
FGF23	chr12	4477392	4488894	11503	fibroblast growth factor 23
FGF6	chr12	4543307	4554780	11474	fibroblast growth factor 6
C12orf4	chr12	4596900	4647637	50738	chromosome 12 open reading frame 4
RAD51AP1	chr12	4647949	4669213	21265	RAD51 associated protein 1
DYRK4	chr12	4671369	4723054	51686	dual-specificity tyrosine-(Y)-phosphorylation regulated kinase 4
AKAP3	chr12	4724673	4758213	33541	A kinase (PRKA) anchor protein 3
NDUFA9	chr12	4758263	4796720	38458	NADH dehydrogenase (ubiquinone) 1 alpha subcomplex, 9, 39kDa
LOC101929549	chr12	4809582	4829268	19687	uncharacterized LOC101929549
GALNT8	chr12	4829751	4881892	52142	N-acetylgalactosaminyltransferase 8
KCNA6	chr12	4918341	4960278	41938	potassium voltage-gated channel, shaker-related subfamily, member 6
KCNA1	chr12	5019072	5027422	8351	potassium voltage-gated channel, shaker-related subfamily, member 1 (episodic ataxia with myokymia)
KCNA5	chr12	5153084	5155954	2871	potassium voltage-gated channel, shaker-related subfamily, member 5
LOC101929584	chr12	5343161	5352317	9157	uncharacterized LOC101929584
BC031884	chr12	5347213	5352317	5105	
NTF3	chr12	5541279	5604465	63187	neurotrophin 3
ANO2	chr12	5671816	6055398	383583	anoctamin 2, calcium activated chloride channel
VWF	chr12	6058039	6233836	175798	von Willebrand factor
Metazoa_SRP	chr12	6204863	6205136	274	
CD9	chr12	6309481	6347437	37957	CD9 molecule
PLEKHG6	chr12	6419601	6437672	18072	pleckstrin homology domain containing, family G (with RhoGef domain) member 6
TNFRSF1A	chr12	6437922	6451283	13362	tumor necrosis factor receptor superfamily, member 1A
SCNN1A	chr12	6456008	6486523	30516	sodium channel, non-voltage-gated 1 alpha subunit
LTBR	chr12	6484533	6500737	16205	lymphotoxin beta receptor (TNFR superfamily, member 3)
CD27-AS1	chr12	6548166	6560683	12518	CD27 antisense RNA 1
CD27	chr12	6554050	6560884	6835	CD27 molecule
TAPBPL	chr12	6561176	6575601	14426	TAP binding protein-like
VAMP1	chr12	6571403	6580065	8663	vesicle-associated membrane protein 1 (synaptobrevin 1)
MRPL51	chr12	6601315	6602471	1157	mitochondrial ribosomal protein L51
SCARNA10	chr12	6619387	6619717	331	small Cajal body-specific RNA 10
NCAPD2	chr12	6603297	6641132	37836	non-SMC condensin I complex, subunit D2
GAPDH	chr12	6643570	6647541	3972	glyceraldehyde-3-phosphate dehydrogenase
DL491527	chr12	6645665	6645791	127	
IFFO1	chr12	6647538	6665249	17712	intermediate filament family orphan 1
NOP2	chr12	6666035	6677498	11464	NOP2 nucleolar protein
SCARNA11	chr12	6690638	6690775	138	small Cajal body-specific RNA 11
AK096395	chr12	6687787	6693905	6119	
CHD4	chr12	6679247	6716599	37353	chromodomain helicase DNA binding protein 4
LPAR5	chr12	6728000	6748203	20204	lysophosphatidic acid receptor 5
ACRBP	chr12	6747241	6756580	9340	acrosin binding protein
ING4	chr12	6759703	6772308	12606	inhibitor of growth family, member 4
ZNF384	chr12	6775642	6798738	23097	zinc finger protein 384
PIANP	chr12	6802956	6810009	7054	PILR alpha associated neural protein
COPS7A	chr12	6833149	6841041	7893	COP9 signalosome subunit 7A
MLF2	chr12	6857157	6876641	19485	myeloid leukemia factor 2
PTMS	chr12	6875540	6880118	4579	parathymosin
LAG3	chr12	6881669	6887621	5953	lymphocyte-activation gene 3
CD4	chr12	6898637	6929976	31340	CD4 molecule
GPR162	chr12	6930962	6939175	8214	G protein-coupled receptor 162
LEPREL2	chr12	6937537	6949018	11482	leprecan-like 2
GNB3	chr12	6949117	6956559	7443	guanine nucleotide binding protein (G protein), beta polypeptide 3
AK097957	chr12	6954022	6957970	3949	
CDCA3	chr12	6953962	6961230	7269	cell division cycle associated 3
USP5	chr12	6961284	6975795	14512	ubiquitin specific peptidase 5 (isopeptidase T)
TPI1	chr12	6976583	6980110	3528	triosephosphate isomerase 1
SPSB2	chr12	6980099	6982521	2423	splA/ryanodine receptor domain and SOCS box containing 2
RRPL13L	chr12	6982789	6993768	10980	
RPL13P5	chr12	6993144	6993768	625	ribosomal protein L13 pseudogene 5
DSTNP2	chr12	6993845	6994950	1106	destrin (actin depolymerizing factor) pseudogene 2
LRRC23	chr12	7013896	7023406	9511	leucine rich repeat containing 23
ENO2	chr12	7023613	7032859	9247	enolase 2 (gamma, neuronal)
ATN1	chr12	7033625	7051484	17860	atrophin 1
C12orf57	chr12	7052140	7055166	3027	chromosome 12 open reading frame 57
PTPN6	chr12	7055739	7070479	14741	protein tyrosine phosphatase, non-receptor type 6
MIR200C	chr12	7072861	7072929	69	microRNA 200c
MIR141	chr12	7073259	7073354	96	microRNA 141
SCARNA12	chr12	7076499	7076769	271	small Cajal body-specific RNA 12
PHB2	chr12	7074514	7079916	5403	prohibitin 2
EMG1	chr12	7079943	7105520	25578	EMG1 N1-specific pseudouridine methyltransferase
LPCAT3	chr12	7085346	7125842	40497	lysophosphatidylcholine acyltransferase 3
C1S	chr12	7096259	7178335	82077	complement component 1, s subcomponent
C1R	chr12	7187514	7245043	57530	complement component 1, r subcomponent
C1RL	chr12	7247145	7261874	14730	complement component 1, r subcomponent-like
C1RL-AS1	chr12	7260903	7274447	13545	C1RL antisense RNA 1

RBP5	chr12	7276279	7281466	5188	retinol binding protein 5, cellular
CLSTN3	chr12	7282966	7311530	28565	calsyntenin 3
PEX5	chr12	7341758	7371169	29412	peroxisomal biogenesis factor 5
ACSM4	chr12	7456927	7480969	24043	acyl-CoA synthetase medium-chain family member 4
CD163L1	chr12	7507555	7596781	89227	CD163 molecule-like 1
CD163	chr12	7623411	7656414	33004	CD163 molecule
APOBEC1	chr12	7801995	7818502	16508	apolipoprotein B mRNA editing enzyme, catalytic polypeptide 1
GDF3	chr12	7842380	7848360	5981	growth differentiation factor 3
DPPA3	chr12	7864088	7870152	6065	developmental pluripotency associated 3
CLEC4C	chr12	7882010	7902069	20060	C-type lectin domain family 4, member C
NANOGNB	chr12	7917811	7926717	8907	NANOG neighbor homeobox
NANOG	chr12	7941991	7948657	6667	Nanog homeobox
Y_RNA	chr12	7999883	7999978	96	
SLC2A14	chr12	7965107	8043792	78686	solute carrier family 2 (facilitated glucose transporter), member 14
CLEC4A	chr12	8276227	8291203	14977	C-type lectin domain family 4, member A
POU5F1P3	chr12	8286364	8287448	1085	POU class 5 homeobox 1 pseudogene 3
ZNF705A	chr12	8325149	8332642	7494	zinc finger protein 705A
FAM66C	chr12	8332804	8368747	35944	family with sequence similarity 66, member C
FAM90A1	chr12	8373855	8380214	6360	family with sequence similarity 90, member A1
FAM86FP	chr12	8383644	8395542	11899	family with sequence similarity 86, member A pseudogene
LOC101927905	chr12	8388010	8395160	7151	uncharacterized LOC101927905
LINC00937	chr12	8509559	8549399	39841	long intergenic non-protein coding RNA 937
CLEC6A	chr12	8608590	8630926	22337	C-type lectin domain family 6, member A
CLEC4D	chr12	8666135	8674960	8826	C-type lectin domain family 4, member D
CLEC4E	chr12	8685900	8693558	7659	C-type lectin domain family 4, member E
AICDA	chr12	8754761	8765442	10682	activation-induced cytidine deaminase
MFAP5	chr12	8798538	8815484	16947	microfibrillar associated protein 5
RIMKLB	chr12	8834272	8935694	101423	ribosomal modification protein rimK-like family member B
A2ML1	chr12	8975149	9039597	64449	alpha-2-macroglobulin-like 1
PHC1	chr12	9067315	9094060	26746	polyhomeotic homolog 1 (Drosophila)
M6PR	chr12	9092956	9102357	9402	mannose-6-phosphate receptor (cation dependent)
KLRG1	chr12	9142220	9163340	21121	killer cell lectin-like receptor subfamily G, member 1
LINC00612	chr12	9208184	9217666	9483	long intergenic non-protein coding RNA 612
A2M-AS1	chr12	9217772	9220651	2880	A2M antisense RNA 1 (head to head)
A2M	chr12	9220303	9268558	48256	alpha-2-macroglobulin
JB175316	chr12	9280497	9280518	22	
PZP	chr12	9301435	9360966	59532	pregnancy-zone protein
A2MP1	chr12	9381128	9386803	5676	alpha-2-macroglobulin pseudogene 1
MIR1244-1	chr12	9392065	9392147	83	microRNA 1244-1
MIR1244-2	chr12	9392065	9392147	83	microRNA 1244-2
MIR1244-3	chr12	9392065	9392147	83	microRNA 1244-3
LINC00987	chr12	9392598	9395645	3048	long intergenic non-protein coding RNA 987
LOC10049940	chr12	9392598	9395645	3048	long intergenic non-protein coding RNA 987
SNORA75	chr12	9439268	9439418	151	small nucleolar RNA, H/ACA box 75
LOC642846	chr12	9436252	9466684	30433	DEAD/H (Asp-Glu-Ala-Asp/His) box polypeptide 11-like
DQ582774	chr12	9481705	9481733	29	
DQ586985	chr12	9481920	9481986	67	
DQ577612	chr12	9485756	9485790	35	
DQ586485	chr12	9488358	9488396	39	
DQ577180	chr12	9488431	9488461	31	
DQ585706	chr12	9488849	9488879	31	
DQ575612	chr12	9492398	9492435	38	
DQ592342	chr12	9499998	9500028	31	
LOC10192803	chr12	9524377	9534018	9642	uncharacterized LOC101928030
LOC10193045	chr12	9520059	9550213	30155	uncharacterized LOC101930452
DQ599803	chr12	9555037	9555064	28	
DDX12P	chr12	9570286	9600768	30483	DEAD/H (Asp-Glu-Ala-Asp/His) box polypeptide 12, pseudogene
SNORA75	chr12	9597653	9597801	149	small nucleolar RNA, H/ACA box 75
BX647938	chr12	9709909	9728864	18956	
KLRB1	chr12	9747869	9760497	12629	killer cell lectin-like receptor subfamily B, member 1
LOC374443	chr12	9769879	9811010	41132	C-type lectin domain family 2, member D pseudogene
CLEC2D	chr12	9822303	9852151	29849	C-type lectin domain family 2, member D
CLECL1	chr12	9868455	9885895	17441	C-type lectin-like 1
CD69	chr12	9905081	9913497	8417	CD69 molecule
KLRF1	chr12	9980076	9997603	17528	killer cell lectin-like receptor subfamily F, member 1
CLEC2B	chr12	10004967	10022458	17492	C-type lectin domain family 2, member B
KLRF2	chr12	10034087	10048432	14346	killer cell lectin-like receptor subfamily F, member 2
CLEC2A	chr12	10051271	10084980	33710	C-type lectin domain family 2, member A
LOC10050615	chr12	10089177	10096094	6918	uncharacterized LOC100506159
CLEC12A	chr12	10103914	10138194	34281	C-type lectin domain family 12, member A
CLEC1B	chr12	10145661	10151899	6239	C-type lectin domain family 1, member B
CLEC12B	chr12	10163230	10171399	8170	C-type lectin domain family 12, member B
LOC10272402	chr12	10167950	10183205	15256	uncharacterized LOC102724020
CLEC9A	chr12	10183275	10218565	35291	C-type lectin domain family 9, member A
CLEC1A	chr12	10222152	10251664	29513	C-type lectin domain family 1, member A
CLEC7A	chr12	10269375	10282868	13494	C-type lectin domain family 7, member A
OLR1	chr12	10310898	10324790	13893	oxidized low density lipoprotein (lectin-like) receptor 1
GABARAPL1	chr12	10365488	10375724	10237	GABA(A) receptor-associated protein like 1
KLRD1	chr12	10457049	10469850	12802	killer cell lectin-like receptor subfamily D, member 1
LOC10192810	chr12	10516367	10551105	34739	uncharacterized LOC101928100

AK096314	chr12	10516367	10551105	34739	
KLRK1	chr12	10524951	10544490	19540	killer cell lectin-like receptor subfamily K, member 1
KLRC4-KLRK1	chr12	10524951	10562745	37795	KLRC4-KLRK1 readthrough
KLRC4	chr12	10559982	10562356	2375	killer cell lectin-like receptor subfamily C, member 4
KLRC3	chr12	10564913	10588592	23680	killer cell lectin-like receptor subfamily C, member 3
KLRC2	chr12	10579192	10588592	9401	killer cell lectin-like receptor subfamily C, member 2
KLRC1	chr12	10594862	10607215	12354	killer cell lectin-like receptor subfamily C, member 1
KLRAP1	chr12	10741076	10752434	11359	killer cell lectin-like receptor subfamily A pseudogene 1
MAGOHB	chr12	10756363	10766208	9846	mago-nashi homolog B (Drosophila)
STYK1	chr12	10771537	10826891	55355	serine/threonine/tyrosine kinase 1
YBX3	chr12	10851675	10875953	24279	Y box binding protein 3
LOC101928162	chr12	10902832	10930050	27219	uncharacterized LOC101928162
TAS2R7	chr12	10954130	10955226	1097	taste receptor, type 2, member 7
TAS2R8	chr12	10958649	10959579	931	taste receptor, type 2, member 8
TAS2R9	chr12	10961692	10962767	1076	taste receptor, type 2, member 9
TAS2R10	chr12	10977944	10978868	925	taste receptor, type 2, member 10
PRR4	chr12	10998447	11002075	3629	proline rich 4 (lacrimal)
TAS2R13	chr12	11060524	11062161	1638	taste receptor, type 2, member 13
PRH2	chr12	11081833	11087444	5612	proline-rich protein HaeIII subfamily 2
TAS2R14	chr12	11090852	11091806	955	taste receptor, type 2, member 14
TAS2R50	chr12	11138511	11139511	1001	taste receptor, type 2, member 50
TAS2R20	chr12	11148560	11150474	1915	taste receptor, type 2, member 20
PRH1-PRR4	chr12	10998447	11324224	325778	PRH1-PRR4 readthrough
TAS2R19	chr12	11174270	11175170	901	taste receptor, type 2, member 19
PRH1	chr12	11033559	11324222	290664	proline-rich protein HaeIII subfamily 1
TAS2R31	chr12	11182985	11184006	1022	taste receptor, type 2, member 31
TAS2R46	chr12	11213963	11214893	931	taste receptor, type 2, member 46
TAS2R43	chr12	11243885	11244912	1028	taste receptor, type 2, member 43
PRB4	chr12	11033559	11463369	429811	proline-rich protein BstNI subfamily 4
TAS2R30	chr12	11285883	11286843	961	taste receptor, type 2, member 30
LOC100129361	chr12	11323779	11328619	4841	chromosome X open reading frame 69-like
TAS2R42	chr12	11338598	11339543	946	taste receptor, type 2, member 42
PRB3	chr12	11418846	11422641	3796	proline-rich protein BstNI subfamily 3
PRB1	chr12	11504756	11508524	3769	proline-rich protein BstNI subfamily 1
PRB2	chr12	11544473	11548498	4026	proline-rich protein BstNI subfamily 2
LINC01252	chr12	11700963	11717335	16373	long intergenic non-protein coding RNA 1252
LOC338817	chr12	11700963	11717335	16373	long intergenic non-protein coding RNA 1252
RNU6-19P	chr12	11944823	12079107	134285	RNA, U6 small nuclear 19, pseudogene
ETV6	chr12	11802787	12268261	465475	ets variant 6
BC038742	chr12	12202797	12208703	5907	
MIR1244-1	chr12	12264885	12264967	83	microRNA 1244-1
BCL2L14	chr12	12223877	12363947	140071	BCL2-like 14 (apoptosis facilitator)
LRP6	chr12	12268960	12419811	150852	low density lipoprotein receptor-related protein 6
MANSC1	chr12	12482217	12503169	20953	MANSC domain containing 1
LOH12CR1	chr12	12510012	12619838	109827	loss of heterozygosity, 12, chromosomal region 1
DUSP16	chr12	12626215	12715448	89234	dual specificity phosphatase 16
CREBL2	chr12	12764766	12798042	33277	cAMP responsive element binding protein-like 2
GPR19	chr12	12813994	12849121	35128	G protein-coupled receptor 19
GPRC5A	chr12	13043955	13066600	22646	G protein-coupled receptor, class C, group 5, member A
MIR614	chr12	13068762	13068852	91	microRNA 614
GPRC5D	chr12	13093708	13103318	9611	G protein-coupled receptor, class C, group 5, member D
LOC100506314	chr12	13132770	13137576	4807	uncharacterized LOC100506314
HEBP1	chr12	13127798	13153243	25446	heme binding protein 1
HTR7P1	chr12	13153375	13157764	4390	5-hydroxytryptamine (serotonin) receptor 7 pseudogene 1
AK125676	chr12	13153380	13192604	39225	
5S_rRNA	chr12	13199088	13199193	106	
KIAA1467	chr12	13197314	13236383	39070	KIAA1467
GSG1	chr12	13236470	13256630	20161	germ cell associated 1
EMP1	chr12	13349601	13369708	20108	epithelial membrane protein 1
C12orf36	chr12	13523604	13529679	6076	chromosome 12 open reading frame 36
7SK	chr12	13836475	13836820	346	RNA, 7SK small nuclear
GRIN2B	chr12	13714409	14133022	418614	glutamate receptor, ionotropic, N-methyl D-aspartate 2B
BC067269	chr12	14577938	14579759	1822	
ATF7IP	chr12	14518565	14655869	137305	activating transcription factor 7 interacting protein
PLBD1	chr12	14656596	14720791	64196	phospholipase B domain containing 1
PLBD1-AS1	chr12	14720665	14772689	52025	PLBD1 antisense RNA 1
GUCY2C	chr12	14765567	14849519	83953	guanylate cyclase 2C (heat stable enterotoxin receptor)
HIST4H4	chr12	14920932	14924065	3134	histone cluster 4, H4
H2AFJ	chr12	14927269	14930936	3668	H2A histone family, member J
WBP11	chr12	14939411	14956401	16991	WW domain binding protein 11
SMCO3	chr12	14957583	14967116	9534	single-pass membrane protein with coiled-coil domains 3
C12orf60	chr12	14956505	14976791	20287	chromosome 12 open reading frame 60
ART4	chr12	14982244	14996413	14170	ADP-ribosyltransferase 4 (Dombrock blood group)
MGP	chr12	15034114	15038853	4740	matrix Gla protein
ERP27	chr12	15066960	15091483	24524	endoplasmic reticulum protein 27
ARHGDIB	chr12	15094949	15114562	19614	Rho GDP dissociation inhibitor (GDI) beta
PDE6H	chr12	15125955	15134799	8845	phosphodiesterase 6H, cGMP-specific, cone, gamma
LINC01489	chr12	15154766	15159617	4852	long intergenic non-protein coding RNA 1489
RERG-AS1	chr12	15304856	15308215	3360	RERG antisense RNA 1
RERG	chr12	15260715	15374411	113697	RAS-like, estrogen-regulated, growth inhibitor

PTPRO	chr12	15475190	15751265	276076	protein tyrosine phosphatase, receptor type, O
EPS8	chr12	15773074	15942510	169437	epidermal growth factor receptor pathway substrate 8
STRAP	chr12	16035287	16056410	21124	serine/threonine kinase receptor associated protein
DERA	chr12	16064105	16190315	126211	deoxyribose-phosphate aldolase (putative)
SLC15A5	chr12	16341418	16430619	89202	solute carrier family 15, member 5
MGST1	chr12	16500075	16530123	30049	microsomal glutathione S-transferase 1
LMO3	chr12	16701305	16762758	61454	LIM domain only 3 (rhombotin-like 2)
SKP1P2	chr12	17141680	17143562	1883	S-phase kinase-associated protein 1 pseudogene 2
U6	chr12	17224331	17224439	109	RNA, U6 small nuclear 50, pseudogene
MIR3974	chr12	17826232	17826328	97	microRNA 3974
RERGL	chr12	18233802	18243127	9326	RERG/RAS-like
PIK3C2G	chr12	18414473	18801352	386880	phosphatidylinositol-4-phosphate 3-kinase, catalytic subunit type 2 gamma
PLCZ1	chr12	18836109	18890993	54885	phospholipase C, zeta 1
CAPZA3	chr12	18891044	18892122	1079	capping protein (actin filament) muscle Z-line, alpha 3
PLEKHA5	chr12	19282625	19529333	246709	pleckstrin homology domain containing, family A member 5
AEBP2	chr12	19592607	19675173	82567	AE binding protein 2
LOC100506393	chr12	20167618	20251802	84185	uncharacterized LOC100506393
PDE3A	chr12	20522178	20837041	314864	phosphodiesterase 3A, cGMP-inhibited
SLCO1C1	chr12	20848288	20906320	58033	solute carrier organic anion transporter family, member 1C1
SLCO1B3	chr12	20963637	21243040	279404	solute carrier organic anion transporter family, member 1B3
SLCO1B7	chr12	20968607	21243040	274434	solute carrier organic anion transporter family, member 1B7 (non-functional)
SLCO1B1	chr12	21284127	21392730	108604	solute carrier organic anion transporter family, member 1B1
SLCO1A2	chr12	21417533	21548371	130839	solute carrier organic anion transporter family, member 1A2
IAPP	chr12	21525801	21532914	7114	islet amyloid polypeptide
PYROXD1	chr12	21590537	21624182	33646	pyridine nucleotide-disulphide oxidoreductase domain 1
RECQL	chr12	21621843	21654603	32761	RecQ helicase-like
GOLT1B	chr12	21654698	21671337	16640	golgi transport 1B
C12orf39	chr12	21679255	21684210	4956	spexin hormone
SPX	chr12	21679255	21684210	4956	spexin hormone
GYS2	chr12	21689122	21757781	68660	glycogen synthase 2 (liver)
LDHB	chr12	21788274	21810789	22516	lactate dehydrogenase B
KCNJ8	chr12	21917888	21927755	9868	potassium inwardly-rectifying channel, subfamily J, member 8
ABCC9	chr12	21950323	22094336	144014	ATP-binding cassette, sub-family C (CFTR/MRP), member 9
CMAS	chr12	22199109	22218606	19498	cytidine monophosphate N-acetylneuraminic acid synthetase
ST8SIA1	chr12	22346324	22487648	141325	ST8 alpha-N-acetyl-neuraminide alpha-2,8-sialyltransferase 1
C2CD5	chr12	22601479	22697480	96002	C2 calcium-dependent domain containing 5
ETNK1	chr12	22778075	22843608	65534	ethanolamine kinase 1
Mir_720	chr12	22853863	22853963	101	
AK094733	chr12	23117505	23327057	209553	
LOC101928441	chr12	23328569	23344521	15953	uncharacterized LOC101928441
SOX5	chr12	23685230	24715383	1030154	SRY (sex determining region Y)-box 5
MIR920	chr12	24365354	24365429	76	microRNA 920
LOC101928471	chr12	24376204	24390899	14696	uncharacterized LOC101928471
LINC00477	chr12	24719897	24737102	17206	long intergenic non-protein coding RNA 477
BCAT1	chr12	24962957	25102393	139437	branched chain amino-acid transaminase 1, cytosolic
C12orf77	chr12	25146364	25150453	4090	chromosome 12 open reading frame 77
LRMP	chr12	25205180	25261269	56090	lymphoid-restricted membrane protein
CASC1	chr12	25261222	25348094	86873	cancer susceptibility candidate 1
LYRM5	chr12	25348149	25357949	9801	LYR motif containing 5
KRAS	chr12	25357722	25403865	46144	Kirsten rat sarcoma viral oncogene homolog
DD157417	chr12	25398274	25398297	24	
IFLTD1	chr12	25629015	25801496	172482	lamin tail domain containing 1
LMNTD1	chr12	25629015	25801496	172482	lamin tail domain containing 1
MIR4302	chr12	26026952	26027012	61	microRNA 4302
RASSF8-AS1	chr12	26097789	26112375	14587	RASSF8 atnisense RNA 1
RASSF8	chr12	26111963	26232825	120863	Ras association (RalGDS/AF-6) domain family (N-terminal) member 8
BHLHE41	chr12	26272958	26278003	5046	basic helix-loop-helix family, member e41
BC041929	chr12	26287896	26334217	46322	
SSPN	chr12	26348268	26405853	57586	sarcospan
ITPR2	chr12	26488284	26986131	497848	inositol 1,4,5-trisphosphate receptor, type 2
ASUN	chr12	27058111	27091254	33144	asunder spermatogenesis regulator
FGFR1OP2	chr12	27091304	27119581	28278	FGFR1 oncogene partner 2
TM7SF3	chr12	27124505	27167339	42835	transmembrane 7 superfamily member 3
MED21	chr12	27175454	27183606	8153	mediator complex subunit 21
C12orf71	chr12	27233989	27235455	1467	chromosome 12 open reading frame 71
STK38L	chr12	27397077	27478890	81814	serine/threonine kinase 38 like
ARNTL2	chr12	27485786	27578746	92961	aryl hydrocarbon receptor nuclear translocator-like 2
BC043511	chr12	27542721	27599558	56838	
ARNTL2-AS1	chr12	27542721	27599567	56847	ARNTL2 antisense RNA 1
SMCO2	chr12	27619742	27655118	35377	single-pass membrane protein with coiled-coil domains 2
PPFIBP1	chr12	27677044	27848497	171454	PTPRF interacting protein, binding protein 1 (liprin beta 1)
AK000807	chr12	27809602	27810048	447	
TRNA_Lys	chr12	27843305	27843378	74	
REP15	chr12	27849427	27850566	1140	RAB15 effector protein
MRPS35	chr12	27863705	27909237	45533	mitochondrial ribosomal protein S35
MANSC4	chr12	27915598	27924209	8612	MANSC domain containing 4
KLHL42	chr12	27933186	27955973	22788	kelch-like family member 42
PTHLH	chr12	28111016	28124916	13901	parathyroid hormone-like hormone
CCDC91	chr12	28332209	28703099	370891	coiled-coil domain containing 91
FAR2	chr12	29301935	29488549	186615	fatty acyl CoA reductase 2

LOC10050660€	chr12	29433348	29470781	37434	uncharacterized LOC100506606
AX746523	chr12	29433350	29470781	37432	
ERGIC2	chr12	29493578	29534143	40566	ERGIC and golgi 2
OVCH1-AS1	chr12	29542226	29640257	98032	OVCH1 antisense RNA 1
OVCH1	chr12	29580488	29650619	70132	ovochymase 1
TMTC1	chr12	29653745	29937692	283948	transmembrane and tetratricopeptide repeat containing 1
IPO8	chr12	30781914	30848929	67016	importin 8
CAPRIN2	chr12	30862485	30907448	44964	caprin family member 2
LINC00941	chr12	30948614	30955645	7032	long intergenic non-protein coding RNA 941
TSPAN11	chr12	31079361	31149537	70177	tetraspanin 11
DDX11-AS1	chr12	31173696	31226781	53086	DDX11 antisense RNA 1
DDX11	chr12	31226778	31257725	30948	DEAD/H (Asp-Glu-Ala-Asp/His) box helicase 11
DKFZp434C063	chr12	31264585	31274400	9816	
OVOS2	chr12	31267684	31359088	91405	ovostatin 2
hsa-miR-3194-3	chr12	31379257	31379277	21	
FAM60A	chr12	31433519	31479159	45641	family with sequence similarity 60, member A
FLJ13224	chr12	31477249	31478879	1631	uncharacterized LOC79857
BC039477	chr12	31516414	31522235	5822	
DENND5B	chr12	31535156	31743952	208797	DENN/MADD domain containing 5B
U5	chr12	31747026	31747140	115	
DENND5B-AS1	chr12	31742856	31768285	25430	DENND5B antisense RNA 1
METTL20	chr12	31800093	31822016	21924	methyltransferase like 20
AMN1	chr12	31824070	31882108	58039	antagonist of mitotic exit network 1 homolog (S. cerevisiae)
H3F3C	chr12	31944118	31945175	1058	H3 histone, family 3C
KIAA1551	chr12	32112352	32146043	33692	KIAA1551
RNU6-78P	chr12	32146772	32146802	31	RNA, U6 small nuclear 78, pseudogene
BICD1	chr12	32260184	32531141	270958	bicaudal D homolog 1 (Drosophila)
TCRA	chr14	22314718	22534581	219864	T cell receptor alpha constant
TRA@	chr14	22362569	22534581	172013	
TRAV20	chr14	22508842	22509396	555	T cell receptor alpha variable 20
AV30S1	chr14	22509128	22509405	278	
TCRA	chr14	22538905	22539467	563	T cell receptor alpha constant
TCRVA13	chr14	22539214	22539470	257	
AV2S1A1	chr14	22356036	22745911	389876	
TCRA	chr14	22554668	22555238	571	T cell receptor alpha constant
TCRAVN1	chr14	22580299	22580985	687	
TRA@	chr14	22591337	22592252	916	
TCRA	chr14	22600965	22600989	25	T cell receptor alpha constant
TCRA	chr14	22636292	22636879	588	T cell receptor alpha constant
U39094	chr14	22636757	22636881	125	
TCRA	chr14	22293655	23021097	727443	T cell receptor alpha constant
TCR-alpha	chr14	22336979	23000088	663110	
V alpha immuno	chr14	22675417	22676029	613	
AV25S1	chr14	22689762	22690367	606	
TRA	chr14	22458652	23016719	558068	T cell receptor alpha locus
TCR	chr14	22739820	22740442	623	
T-cell receptor a	chr14	22740275	22740445	171	
TRD	chr14	22465824	23016719	550896	T cell receptor delta locus
TRAC	chr14	22466122	23021097	554976	T cell receptor alpha constant
av27s1	chr14	22771875	22772438	564	
TCR- alpha V 3	chr14	22782884	22783343	460	
TCR-alpha	chr14	22788567	22789122	556	
hADV29S1	chr14	22631190	23000088	368899	T cell receptor alpha variable 29/delta variable 5 (gene/pseudogene)
TRA@	chr14	22615959	23021097	405139	
AV4S1	chr14	22670486	22981890	311405	
hADV36S1	chr14	22694611	23016719	322109	T cell receptor alpha variable 36/delta variable 7
hADV38S2	chr14	22748987	22962976	213990	T cell receptor alpha variable 38-2/delta variable 8
hDV102S1	chr14	22891471	22892033	563	T cell receptor delta variable 2
AK125397	chr14	22886527	22901726	15200	
AK093552	chr14	22849082	22951948	102867	
TCRDV2J2	chr14	22918106	22925700	7595	
TRDC	chr14	22919080	22934912	15833	T cell receptor delta constant
TCRDV2	chr14	22928088	22934912	6825	
hDV103S1	chr14	22938034	22938658	625	T cell receptor delta variable 3
X61074	chr14	22946618	22948187	1570	
ATP9B	chr18	76829396	77138282	308887	ATPase, class II, type 9B
RBFADN	chr18	77806899	77839206	32308	RBFA downstream neighbor (non-protein coding)
RBFA	chr18	77794345	77853788	59444	ribosome binding factor A (putative)
ADNP2	chr18	77866914	77898228	31315	ADNP homeobox 2
PARD6G-AS1	chr18	77905806	77936315	30510	PARD6G antisense RNA 1
PARD6G	chr18	77915116	78005397	90282	par-6 family cell polarity regulator gamma
IGLL1	chr22	23915312	23922495	7184	immunoglobulin lambda-like polypeptide 1
DQ586720	chr22	23952777	23952803	27	
C22orf43	chr22	23950638	23974508	23871	aspartate-rich 1
DRICH1	chr22	23950638	23974508	23871	aspartate-rich 1
GUSBP11	chr22	23980674	24059610	78937	glucuronidase, beta pseudogene 11
RGL4	chr22	24032960	24041363	8404	ral guanine nucleotide dissociation stimulator-like 4
ZNF70	chr22	24083771	24093279	9509	zinc finger protein 70
VPREB3	chr22	24094929	24096630	1702	pre-B lymphocyte 3
C22orf15	chr22	24105207	24108050	2844	chromosome 22 open reading frame 15

CHCHD10	chr22	24108020	24110159	2140	coiled-coil-helix-coiled-coil-helix domain containing 10
MMP11	chr22	24115035	24126503	11469	matrix metallopeptidase 11 (stromelysin 3)
CABIN1	chr22	24407764	24574596	166833	calcineurin binding protein 1
SUSD2	chr22	24577443	24585074	7632	sushi domain containing 2
GGT5	chr22	24615621	24641110	25490	gamma-glutamyltransferase 5
UNQ2565	chr22	24647952	24649256	1305	POM121-like
POM121L9P	chr22	24647588	24661492	13905	POM121 transmembrane nucleoporin-like 9, pseudogene
DQ576853	chr22	24658457	24658483	27	
SPECC1L	chr22	24666784	24838325	171542	sperm antigen with calponin homology and coiled-coil domains 1-like
SPECC1L-ADC	chr22	24666784	24838328	171545	SPECC1L-ADORA2A readthrough (NMD candidate)
ADORA2A	chr22	24813708	24838328	24621	adenosine A2a receptor
EU036692	chr22	24827818	24828694	877	
ADORA2A-AS1	chr22	24825177	24890783	65607	ADORA2A antisense RNA 1
UPB1	chr22	24863205	24922553	59349	ureidopropionase, beta
PIWIL3	chr22	25115000	25170687	55688	piwi-like RNA-mediated gene silencing 3
TOP1P2	chr22	25160467	25161986	1520	topoisomerase (DNA) I pseudogene 2
CRYBB2	chr22	25615611	25627836	12226	crystallin, beta B2
BC040576	chr22	25677208	25679061	1854	
IGLL3P	chr22	25714223	25716193	1971	immunoglobulin lambda-like polypeptide 3, pseudogene
LRP5L	chr22	25747384	25777544	30161	low density lipoprotein receptor-related protein 5-like
MIR6817	chr22	25851612	25851678	67	microRNA 6817
CRYBB2P1	chr22	25844053	25907585	63533	crystallin, beta B2 pseudogene 1
ADRBK2	chr22	25960860	26125258	164399	adrenergic, beta, receptor kinase 2
MYO18B	chr22	26138119	26427007	288889	myosin XVIIIB
SEZ6L	chr22	26565439	26779563	214125	seizure related 6 homolog (mouse)-like
ASPHD2	chr22	26825279	26840978	15700	aspartate beta-hydroxylase domain containing 2
HPS4	chr22	26846848	26879829	32982	Hermansky-Pudlak syndrome 4
SRRD	chr22	26879845	26887904	8060	SRR1 domain containing
TFIP11	chr22	26887893	26908437	20545	tuftelin interacting protein 11
MIR548J	chr22	26951177	26951289	113	microRNA 548j
TPST2	chr22	26921713	26986089	64377	tyrosylprotein sulfotransferase 2
CRYBB1	chr22	26995361	27013991	18631	crystallin, beta B1
CRYBA4	chr22	27017927	27026636	8710	crystallin, beta A4
MIAT	chr22	27053445	27072440	18996	myocardial infarction associated transcript (non-protein coding)
AK124820	chr22	27063655	27068617	4963	
AK026502	chr22	27068848	27114949	46102	
MIATNB	chr22	27068805	27176856	108052	MIAT neighbor (non-protein coding)
LINC01422	chr22	27299254	27316573	17320	long intergenic non-protein coding RNA 1422
AK055980	chr22	27444105	27456467	12363	
BC015159	chr22	27619257	27622760	3504	
AX748308	chr22	28276021	28277106	1086	
PITPNB	chr22	28247656	28315294	67639	phosphatidylinositol transfer protein, beta
MIR3199-1	chr22	28316512	28316600	89	microRNA 3199-1
MIR3199-2	chr22	28316513	28316599	87	microRNA 3199-2
TTC28-AS1	chr22	28315363	28404267	88905	TTC28 antisense RNA 1
TTC28	chr22	28374001	29075853	701853	tetratricopeptide repeat domain 28
MIR5739	chr22	28855856	28855936	81	microRNA 5739
CHEK2	chr22	29083730	29137822	54093	checkpoint kinase 2
ZNRF3	chr22	29279754	29453476	173723	zinc and ring finger 3
ZNRF3-AS1	chr22	29420986	29427464	6479	ZNRF3 antisense RNA 1
C22orf31	chr22	29454659	29457907	3249	chromosome 22 open reading frame 31
KREMEN1	chr22	29469065	29564321	95257	kringle containing transmembrane protein 1
EMID1	chr22	29601900	29655586	53687	EMI domain containing 1
RHBDD3	chr22	29655843	29663914	8072	rhomboid domain containing 3
EWSR1	chr22	29663997	29696515	32519	EWS RNA-binding protein 1
RFPL1S	chr22	29833003	29838118	5116	RFPL1 antisense RNA 1
RFPL1	chr22	29834571	29838444	3874	ret finger protein-like 1
NEFH	chr22	29876180	29887277	11098	neurofilament, heavy polypeptide
KIAA0845	chr22	29885264	29887277	2014	
THOC5	chr22	29904155	29949736	45582	THO complex 5
NIPSNAP1	chr22	29950797	29977326	26530	nipsnap homolog 1 (C. elegans)
NF2	chr22	29999544	30094589	95046	neurofibromin 2 (merlin)
CABP7	chr22	30116343	30127820	11478	calcium binding protein 7
ZMAT5	chr22	30126944	30163000	36057	zinc finger, matrin-type 5
MTMR3	chr22	30279157	30426857	147701	myotubularin related protein 3
MIR6818	chr22	30403037	30403102	66	microRNA 6818
HORMAD2-AS1	chr22	30404730	30476469	71740	HORMAD2 antisense RNA 1
HORMAD2	chr22	30476452	30573062	96611	HORMA domain containing 2
PES1	chr22	30972611	31003000	30390	pescadillo ribosomal biogenesis factor 1
TCN2	chr22	31003069	31023047	19979	transcobalamin II
SLC35E4	chr22	31031792	31065003	33212	solute carrier family 35, member E4
DUSP18	chr22	31048037	31063872	15836	dual specificity phosphatase 18
MIR3200	chr22	31127543	31127628	86	microRNA 3200
OSBP2	chr22	31089768	31303811	214044	oxysterol binding protein 2
MORC2-AS1	chr22	31318294	31322640	4347	MORC2 antisense RNA 1
MORC2	chr22	31322599	31364187	41589	MORC family CW-type zinc finger 2
TUG1	chr22	31365196	31375380	10185	taurine up-regulated 1 (non-protein coding)
SMTN	chr22	31477281	31500610	23330	smoothelin
AX747817	chr22	31497861	31500743	2883	
SELM	chr22	31500762	31503551	2790	selenoprotein M

INPP5J	chr22	31503960	31530683	26724	inositol polyphosphate-5-phosphatase J
PLA2G3	chr22	31530792	31536593	5802	phospholipase A2, group III
MIR3928	chr22	31556047	31556105	59	microRNA 3928
RNF185	chr22	31556137	31603005	46869	ring finger protein 185
BC069815	chr22	31601248	31601602	355	
Y_RNA	chr22	31626056	31626158	103	
LIMK2	chr22	31608249	31676066	67818	LIM domain kinase 2
Mir_1302	chr22	32452438	32452490	53	
SLC5A1	chr22	32439018	32509011	69994	solute carrier family 5 (sodium/glucose cotransporter), member 1
AP1B1P1	chr22	32517963	32529456	11494	adaptor-related protein complex 1, beta 1 subunit pseudogene 1
JB175027	chr22	32524336	32524357	22	
C22orf42	chr22	32545518	32555243	9726	chromosome 22 open reading frame 42
RFPL2	chr22	32586421	32600718	14298	ret finger protein-like 2
SLC5A4	chr22	32614462	32651318	36857	solute carrier family 5 (glucose activated ion channel), member 4
JB153905	chr22	32744122	32744144	23	
RFPL3	chr22	32750871	32757148	6278	ret finger protein-like 3
RFPL3S	chr22	32755892	32767251	11360	RFPL3 antisense
LOC339666	chr22	32772650	32780329	7680	uncharacterized LOC339666
RTCB	chr22	32783561	32808274	24714	RNA 2',3'-cyclic phosphate and 5'-OH ligase
BPIFC	chr22	32809833	32860433	50601	BPI fold containing family C
FBXO7	chr22	32870706	32894818	24113	F-box protein 7
AK123891	chr22	32896531	32898414	1884	
5S_rRNA	chr22	33031087	33031184	98	
SYN3	chr22	32908539	33454377	545839	synapsin III
TIMP3	chr22	33196801	33259028	62228	TIMP metallopeptidase inhibitor 3
MIR4764	chr22	33832567	33832655	89	microRNA 4764
LARGE	chr22	33669061	34318584	649524	like-glycosyltransferase
SNORA50	chr22	34100771	34100908	138	small nucleolar RNA, H/ACA box 76A
LARGE-AS1	chr22	34120971	34146803	25833	LARGE antisense RNA 1
ISX	chr22	35462129	35483380	21252	intestine-specific homeobox
HMGXB4	chr22	35653444	35691800	38357	HMG box domain containing 4
TOM1	chr22	35695267	35743987	48721	target of myb1 (chicken)
MIR3909	chr22	35731632	35731751	120	microRNA 3909
MIR6069	chr22	35732713	35732792	80	microRNA 6069
RASD2	chr22	35937351	35950045	12695	RASD family, member 2
MB	chr22	36002810	36019401	16592	myoglobin
LOC284912	chr22	36023037	36031181	8145	uncharacterized LOC284912
APOL6	chr22	36044423	36064456	20034	apolipoprotein L, 6
APOL5	chr22	36113918	36125529	11612	apolipoprotein L, 5
RBFOX2	chr22	36134782	36424585	289804	RNA binding protein, fox-1 homolog (C. elegans) 2
APOL3	chr22	36536370	36562225	25856	apolipoprotein L, 3
APOL4	chr22	36585175	36600879	15705	apolipoprotein L, 4
APOL2	chr22	36622254	36636000	13747	apolipoprotein L, 2
MFNG	chr22	37865100	37882478	17379	MFNG O-fucosylpeptide 3-beta-N-acetylglucosaminyltransferase
CARD10	chr22	37875382	37915378	39997	caspase recruitment domain family, member 10
GTPBP1	chr22	39101806	39129592	27787	GTP binding protein 1
SUN2	chr22	39130718	39190161	59444	Sad1 and UNC84 domain containing 2
DNAL4	chr22	39174512	39190161	15650	dynein, axonemal, light chain 4
NPTXR	chr22	39214455	39240017	25563	neuronal pentraxin receptor
CBX6	chr22	39260247	39268258	8012	chromobox homolog 6
CACNA1I	chr22	39966757	40085740	118984	calcium channel, voltage-dependent, T type, alpha 1I subunit
ENTHD1	chr22	40139048	40289794	150747	ENTH domain containing 1
GRAP2	chr22	40297085	40369346	72262	GRB2-related adaptor protein 2
FAM83F	chr22	40390952	40426043	35092	family with sequence similarity 83, member F
LOC10013089	chr22	40428335	40432581	4247	uncharacterized LOC100130899
TNRC6B	chr22	40440820	40731812	290993	trinucleotide repeat containing 6B
ADSL	chr22	40742503	40762575	20073	adenylosuccinate lyase
SGSM3	chr22	40766594	40806293	39700	small G protein signaling modulator 3
MKL1	chr22	40806284	41032723	226440	Megakaryoblastic leukemia 1
LOC10192725	chr22	40917803	40922711	4909	uncharacterized LOC101927257
LINC01315	chr22	42760405	42765214	4810	long intergenic non-protein coding RNA 1315
BC038245	chr22	42760534	42765180	4647	
NFAM1	chr22	42776413	42828401	51989	NFAT activating protein with ITAM motif 1
SERHL	chr22	42896584	42908566	11983	serine hydrolase-like
RRP7A	chr22	42904340	42915829	11490	ribosomal RNA processing 7 homolog A (S. cerevisiae)
SERHL2	chr22	42949867	42970388	20522	serine hydrolase-like 2
RRP7B	chr22	42951228	42978017	26790	ribosomal RNA processing 7 homolog B (S. cerevisiae)
POLDIP3	chr22	42979726	43010968	31243	polymerase (DNA-directed), delta interacting protein 3
CS330190	chr22	43172869	43172890	22	
DQ595055	chr22	43182195	43182228	34	
ARFGAP3	chr22	43192531	43253408	60878	ADP-ribosylation factor GTPase activating protein 3
PACSIN2	chr22	43265771	43411184	145414	protein kinase C and casein kinase substrate in neurons 2
EFCAB6	chr22	43924623	44208217	283595	EF-hand calcium binding domain 6
AX747137	chr22	44220386	44222913	2528	
SULT4A1	chr22	44220386	44258378	37993	sulfotransferase family 4A, member 1
PNPLA5	chr22	44275557	44287893	12337	patatin-like phospholipase domain containing 5
PNPLA3	chr22	44319618	44360433	40816	patatin-like phospholipase domain containing 3
LOC10192752	chr22	44839206	44840668	1463	uncharacterized LOC101927526
LDOC1L	chr22	44888449	44894005	5557	leucine zipper, down-regulated in cancer 1-like
LINC00207	chr22	44965219	44968329	3111	long intergenic non-protein coding RNA 207

LINC00229	chr22	45002207	45021299	19093	long intergenic non-protein coding RNA 229
PRR5	chr22	45064426	45133561	69136	proline rich 5 (renal)
PRR5-ARHGAF	chr22	45098077	45258664	160588	PRR5-ARHGAP8 readthrough
ARHGAP8	chr22	45148437	45258664	110228	Rho GTPase activating protein 8
PHF21B	chr22	45277042	45405809	128768	PHD finger protein 21B
NUP50-AS1	chr22	45529638	45559662	30025	NUP50 antisense RNA 1 (head to head)
LOC100506714	chr22	45529638	45559662	30025	NUP50 antisense RNA 1 (head to head)
NUP50	chr22	45559725	45583890	24166	nucleoporin 50kDa
ATXN10	chr22	46067677	46241187	173511	ataxin 10
MIR4762	chr22	46156403	46156478	76	microRNA 4762
MIRLET7BHG	chr22	46449725	46509808	60084	MIRLET7B host gene (non-protein coding)
MIRLET7B	chr22	46509565	46509648	84	microRNA let-7b
PPARA	chr22	46546498	46639653	93156	peroxisome proliferator-activated receptor alpha
LOC101927722	chr22	47857047	47882860	25814	uncharacterized LOC101927722
BC037972	chr22	47857047	47882860	25814	
LINC00898	chr22	48016791	48027318	10528	long intergenic non-protein coding RNA 898
BC039485	chr22	48027451	48038545	11095	
LOC284930	chr22	48027422	48251349	223928	uncharacterized LOC284930
AK093107	chr22	48027451	48251349	223899	
MIR3201	chr22	48670175	48670227	53	microRNA 3201
LOC284933	chr22	48934711	48943199	8489	uncharacterized LOC284933
FAM19A5	chr22	48885271	49147747	262477	family with sequence similarity 19 (chemokine (C-C motif)-like), member A5
MIR4535	chr22	49176106	49176165	60	microRNA 4535
LINC01310	chr22	49262581	49294198	31618	long intergenic non-protein coding RNA 1310
LOC10012894€	chr22	49262581	49294198	31618	long intergenic non-protein coding RNA 1310
C22orf34	chr22	49808173	50051190	243018	chromosome 22 open reading frame 34
BC033837	chr22	49808173	50051190	243018	
DMD	chrX	31137344	33357726	2220383	dystrophin
JA783513	chrX	32827657	32827688	32	
JA783507	chrX	32834568	32834591	24	
JA202257	chrX	32834661	32834692	32	
JA783500	chrX	32862888	32862913	26	
JA783498	chrX	32862937	32862967	31	
JA783489	chrX	32867872	32867918	47	
JA783482	chrX	33038257	33038311	55	
