# Supplementary material for: First insight into the somatic mutation burden of neurofibromatosis type 2-associated grade I and grade II meningiomas: a case report comprehensive genomic study of two cranial meningiomas with vastly different clinical presentation
Source: BMC Cancer. 2017 Feb 13;17:127. doi: 10.1186/s12885-017-3127-6 (PMC5307647; doi:10.1186/s12885-017-3127-6)
Supplement: Additional file 4: — Cancer genes affected by losses or gains in the grade II meningioma. (PDF 62 kb) [file 12885_2017_3127_MOESM4_ESM.pdf]

Additional File 4. Cancer genes affected by losses or gains in the grade II meningioma.

Gene Symbol	Name	Chromosome	Chr Band	Start	End	Length	Cancer Somatic Mut	Cancer Germline Mut	Tumour Types (Somatic Mutations)
PRDM16	PR domain containing 16	chr1	1p36.23-p33	2985741	3355185	369445	yes		MDS, AML
CAMTA1	calmodulin binding transcription activator 1	chr1	1p36.31-p36.23	6845383	7829766	984384	yes		epithelioid haemangioendothelioma
PAX7	paired box gene 7	chr1	1p36.2-p36.12	18957499	19075360	117862	yes		alveolar rhabdomyosarcoma
LCK	lymphocyte-specific protein tyrosine kinase	chr1	1p35-p34.3	32716839	32751768	34930	yes		T-ALL
SFPQ	splicing factor proline/glutamine rich(polypyrimidine tract bindi	chr1	1p34.3	35649200	35658743	9544	yes		papillary renal
CSF3R	colony stimulating factor 3 receptor (granulocyte)	chr1	1p35-p34.3	36931643	36948915	17273	yes		aCML, chronic neutrophilic leukaemia
MYCL1	v-myc myelocytomatosis viral oncogene homolog 1, lung carc	chr1	1p34.3	40361095	40367687	6593	yes		small cell lung carcinoma
TAL1	T-cell acute lymphocytic leukemia 1 (SCL)	chr1	1p32	47681961	47698007	16047	yes		lymphoblastic leukaemia/biphasic
CDKN2C	cyclin-dependent kinase inhibitor 2C (p18, inhibits CDK4)	chr1	1p32	51434366	51440309	5944	yes		glioma, MM
EPS15	epidermal growth factor receptor pathway substrate 15 (AF1p	chr1	1p32	51819934	51984995	165062	yes		ALL
JUN	jun oncogene	chr1	1p32-p31	59246462	59249785	3324	yes		sarcoma
JAK1	Janus kinase 1	chr1	1p32.3-p31.3	65298905	65432619	133715	yes		ALL
BCL10	B-cell CLL/lymphoma 10	chr1	1p22	85731459	85742587	11129	yes		MALT
RBM15	RNA binding motif protein 15	chr1	1p13	110881944	110889303	7360	yes		acute megakaryocytic leukaemia
TRIM33	tripartite motif-containing 33 (PTC7,TIF1G)	chr1	1p13	114935398	115053781	118384	yes		papillary thyroid
NRAS	neuroblastoma RAS viral (v-ras) oncogene homolog	chr1	1p13.2	115247084	115259515	12432	yes		melanoma, MM, AML, thyroid
ARNT	aryl hydrocarbon receptor nuclear translocator	chr1	1q21	150782180	150849244	67065	yes		AML
TPM3	tropomyosin 3	chr1	1q22-q23	154127779	154164611	36833	yes		papillary thyroid, ALCL, NSCLC
MUC1	mucin 1, transmembrane	chr1	1q21	155158299	155162706	4408	yes		B-NHL
PRCC	papillary renal cell carcinoma (translocation-associated)	chr1	1q21.1	156737273	156770609	33337	yes		papillary renal
NTRK1	neurotrophic tyrosine kinase, receptor, type 1	chr1	1q21-q22	156785541	156851642	66102	yes		papillary thyroid
SDHC	succinate dehydrogenase complex, subunit C, integral memb	chr1	1q21	161284165	161334535	50371		yes	
FCGR2B	Fc fragment of IgG, low affinity IIb, receptor for (CD32)	chr1	1q23	161632904	161648444	15541	yes		ALL
PBX1	pre-B-cell leukemia transcription factor 1	chr1	1q23	164528596	164854300	325705	yes		pre B-ALL, myoepithelioma
ABL2	v-abl Abelson murine leukemia viral oncogene homolog 2	chr1	1q24-q25	179068461	179198819	130359	yes		AML
TPR	translocated promoter region	chr1	1q25	186280785	186344864	64080	yes		papillary thyroid
PTPRC	protein tyrosine phosphatase, receptor type, C	chr1	1q31-q32	198608097	198726605	118509	yes		T-ALL
MDM4	Mdm4 p53 binding protein homolog	chr1	1q32	204485506	204677661	192156	yes		glioblastoma, bladder, retinoblastoma
ELK4	ELK4, ETS-domain protein (SRF accessory protein 1)	chr1	1q32	205566694	205649630	82937	yes		prostate
SLC45A3	solute carrier family 45, member 3	chr1	1q32	205592803	205649630	56828	yes		prostate
H3F3A	H3 histone, family 3A	chr1	1q42.12	226250407	226259703	9297	yes		glioma
PRDM1	PR domain containing 1, with ZNF domain	chr6	6q21	106534194	106557814	23621	yes		DLBCL
ROS1	v-ros UR2 sarcoma virus oncogene homolog 1 (avian)	chr6	6q22	117609529	117747018	137490	yes		glioblastoma, NSCLC
GOPC	golgi associated PDZ and coiled-coil motif containing	chr6	6q21	117639350	117923705	284356	yes		glioblastoma
STL	Six-twelve leukemia gene	chr6	6q23	125229391	125284173	54783	yes		B-ALL
MYB	v-myb myeloblastosis viral oncogene homolog	chr6	6q22-23	135502452	135540311	37860	yes		adenoid cystic carcinoma
TNFAIP3	tumor necrosis factor, alpha-induced protein 3	chr6	6q23	138188324	138204451	16128	yes		marginal zone B-cell lymphomas, Hodgkin lymphoma, PMBL
ECT2L	epithelial cell transforming sequence 2 oncogene-like	chr6	6q24.1	139117247	139225207	107961	yes		ETP ALL
EZR	ezrin	chr6	6q25.3	159186772	159240456	53685	yes		NSCLC
FGFR1OP	FGFR1 oncogene partner (FOP)	chr6	6q27	167412804	167455906	43103	yes		MPD, NHL
MLLT4	myeloid/lymphoid or mixed-lineage leukemia (trithorax homolo	chr6	6q27	168227670	168372700	145031	yes		AL
HRAS	v-Ha-ras Harvey rat sarcoma viral oncogene homolog	chr11	11p15.5	532241	535550	3310	yes	yes	infrequent sarcomas, rare other tumour types
NUP98	nucleoporin 98kDa	chr11	11p15	3696239	3819022	122784	yes		AML
LMO1	LIM domain only 1 (rhombotin 1) (RBTN1)	chr11	11p15	8245850	8290182	44333	yes	yes	T-ALL, neuroblastoma
KDM5A	lysine (K)-specific demethylase 5A, JARID1A	chr12	12p11	389222	498621	109400	yes		AML
CCND2	cyclin D2	chr12	12p13	4382901	4414522	31622	yes		NHL,CLL
ZNF384	zinc finger protein 384 (CIZ/NMP4)	chr12	12p13	6775642	6798738	23097	yes		ALL
ETV6	ets variant gene 6 (TEL oncogene)	chr12	12p13	11802787	12268261	465475	yes		congenital fibrosarcoma, multiple leukaemia and lymphoma, secretory breast, MDS, ALL
KRAS	v-Ki-ras2 Kirsten rat sarcoma 2 viral oncogene homolog	chr12	12p12.1	25357722	25403865	46144	yes		pancreatic, colorectal, lung, thyroid, AML, other tumour types
TRA@	T cell receptor alpha locus	chr14	14q11.2	22362569	22534581	172013	yes		T-ALL
CHEK2	CHK2 checkpoint homolog (S. pombe)	chr22	22q12.1	29083730	29137822	54093		yes	
EWSR1	Ewing sarcoma breakpoint region 1 (EWS)	chr22	22q12	29663997	29696515	32519	yes		Ewing sarcoma, desmoplastic small round cell tumour , ALL, clear cell sarcoma, sarcoma, myoepithelioma
NF2	neurofibromatosis type 2 gene	chr22	22q12.2	29999544	30094589	95046	yes	yes	meningioma, acoustic neuroma, renal
MKL1	megakaryoblastic leukemia (translocation) 1	chr22	22q13	40806284	41032723	226440	yes		acute megakaryocytic leukaemia
