# Supplementary material for: First insight into the somatic mutation burden of neurofibromatosis type 2-associated grade I and grade II meningiomas: a case report comprehensive genomic study of two cranial meningiomas with vastly different clinical presentation
Source: BMC Cancer. 2017 Feb 13;17:127. doi: 10.1186/s12885-017-3127-6 (PMC5307647; doi:10.1186/s12885-017-3127-6)
Supplement: Additional file 5: — Mutations selected for Sanger verification. (PDF 115 kb) [file 12885_2017_3127_MOESM5_ESM.pdf]

Additional File 5. Mutations selected for Sanger verification.

Gene Name	Chromosome	Position	Reference allele	Alternative allele	Type of mutation	ConsScore	PolyPhen	SIFT	CADD-Score	SampleID
IDH2	15	90628543	A	C	SNV	7	possibly_damaging	deleterious	11.61	Grade2-1, Grade2-2
ADAMTSL3	15	84657544	TGGAAAGT	T	DEL	7	NA	NA	17.6	Grade2-1, Grade2-2, Grade2-3, Grade2-4
SMYD1	2	88387407	T	G	SNV	7	benign	deleterious	18.33	Grade2-2
EPHB3	3	184297360	G	T	SNV	7	probably_damaging	deleterious	19.15	Grade1
SLC35B1	17	47783669	A	C	SNV	7	benign	tolerated	21.9	Grade2-2
ECEL1	2	233348878	A	G	SNV	7	probably_damaging	tolerated	24.1	Grade2-4
BRD8	5	137503737	C	A	SNV	7	probably_damaging	tolerated	24.2	Grade1
SASH1	6	148792578	T	A	SNV	7	probably_damaging	deleterious	25	Grade2-2
IRF2	4	185340674	A	C	SNV	7	benign	deleterious	25.2	Grade2-1
CAPN5	11	76804840	G	A	SNV	7	possibly_damaging	tolerated	25.9	Grade2-1, Grade2-2, Grade2-3, Grade2-4
SLC4A3	2	220504339	C	CA	INS	7	NA	NA	37	Grade2-1
